# An integrated approach towards the development of novel antifungal agents containing thiadiazole: synthesis and a combined similarity search, homology modelling, molecular dynamics and molecular docking study

**DOI:** 10.1186/s13065-018-0485-3

**Published:** 2018-11-23

**Authors:** Mustafa Er, Abdulati Miftah Abounakhla, Hakan Tahtaci, Ali Hasin Bawah, Süleyman Selim Çınaroğlu, Abdurrahman Onaran, Abdulilah Ece

**Affiliations:** 1 Department of Chemistry, Faculty of Science, Karabuk University, 78050 Karabuk, Turkey; 2 Department of Medical Biotechnology, Institute of Health Sciences, Acıbadem Mehmet Ali Aydınlar University, 34752 İstanbul, Turkey; 3 Department of Plant Protection, Faculty of Agriculture, Gaziosmanpasa University, 60250 Tokat, Turkey; 4 Department of Pharmaceutical Chemistry, Faculty of Pharmacy, Biruni University, 34010 Istanbul, Turkey

**Keywords:** 2-Amino-1,3,4-thiadiazole, Acylation, Antifungal, Homology modelling, Molecular dynamics, Molecular docking

## Abstract

**Background:**

This study aims to synthesise and characterise novel compounds containing 2-amino-1,3,4-thiadiazole and their acyl derivatives and to investigate antifungal activities. Similarity search, molecular dynamics and molecular docking were also studied to find out a potential target and enlighten the inhibition mechanism.

**Results:**

As a first step, 2-amino-1,3,4-thiadiazole derivatives (compounds **3** and **4**) were synthesised with high yields (81 and 84%). The target compounds (**6a**–**n** and **7a**–**n**) were then synthesised with moderate to high yields (56–87%) by reacting **3** and **4** with various acyl chloride derivatives (**5a**–**n**). The synthesized compounds were characterized using the IR, ^1^H-NMR, ^13^C-NMR, Mass, X-ray (compound **7n**) and elemental analysis techniques. Later, the in vitro antifungal activities of the synthesised compounds were determined. The inhibition zones exhibited by the compounds against the tested fungi, their minimum fungicidal activities, minimum inhibitory concentration and the lethal dose values (LD_50_) were determined. The compounds exhibited moderate to high levels of activity against all tested pathogens. Finally, in silico modelling was used to enlighten inhibition mechanism using ligand and structure-based methods. As an initial step, similarity search was carried out and the resulting proteins that belong to *Homo sapiens* were used as reference in sequence similarity search to find the corresponding amino acid sequences in target organisms. Homology modelling was used to construct the protein structure. The stabilised protein structure obtained from molecular dynamics simulation was used in molecular docking.

**Conclusion:**

The overall results presented here might be a good starting point for the identification of novel and more active compounds as antifungal agents.
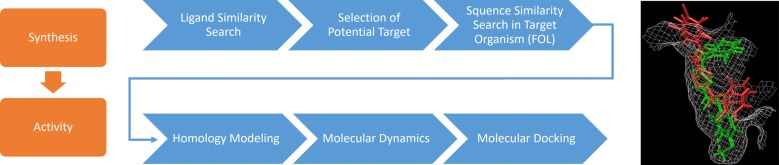

**Electronic supplementary material:**

The online version of this article (10.1186/s13065-018-0485-3) contains supplementary material, which is available to authorized users.

## Background

Due to widespread of infectious diseases killing millions of people, the need for new active, safer and more potential antimicrobial agents has increased dramatically. Accordingly, researchers focus on synthesising novel compounds and their derivatives having different physiochemical properties which promises high activities with no or fewer side effects. Heterocyclic compounds are widespread in nature and are used in many fields. It has been known for many years that heterocyclic compounds, especially those containing nitrogen and sulphur atoms, have a variety of biological activities [1–3].

Thiadiazole is a five-membered heterocyclic ring system which contains two nitrogen and one sulphur atom with the molecular formula of C_2_H_2_N_2_S. 1,3,4-Thiadiazole and its derivatives have become the focus of attention in drug, agriculture and material chemistry due to their high activity in 2′ and 5′ positions in substitution reactions [4, 5].

The two-electron donor nitrogen system (–N=C–S) and hydrogen-binding domain allow for great structural stability and is known to be the component responsible for biological activity [6, 7].

1,3,4-Thiadiazole and its derivatives have an important place among compounds with hetero rings containing nitrogen and sulphur atoms and have been extensively used in pharmaceutics due to their biological activity such as antifungal, antibacterial, antioxidant, anti-inflammatory, anticonvulsant, antituberculosis, and antiproliferative activities [8–20].

The drug design begins with the synthesis of a compound that exhibits a promising biological profile (lead compound), then the activity profile is optimised and finally ends with chemical synthesis of this final compound (drug candidate).

Computer Aided Drug Design helps to design novel and active compounds which also have fewer side effects. In that respect, in silico molecular modelling has been playing an increasingly important role in the development and synthesis of new drug substances and in understanding the basis of drug-target protein interactions [21–23].

In the light of the literature survey, the purpose of this study is to synthesise a number of compounds with different substituted groups containing 1,3,4-thiadiazoles ring and their acyl derivatives, to investigate their antifungal activities and finally to discuss the inhibition mechanism by means of computational tools.

## Results and discussion

### Chemistry

In the first part of the study, the thiadiazole compounds (**3** and **4**) were synthesised from the reaction of the compounds **1** or **2** (purchased) with the thiosemicarbazide in trifluoroacetic acid (TFA) at 60 °C. The compounds **3** and **4** were obtained as specified in the literature [24, 25].

**Scheme 1:**
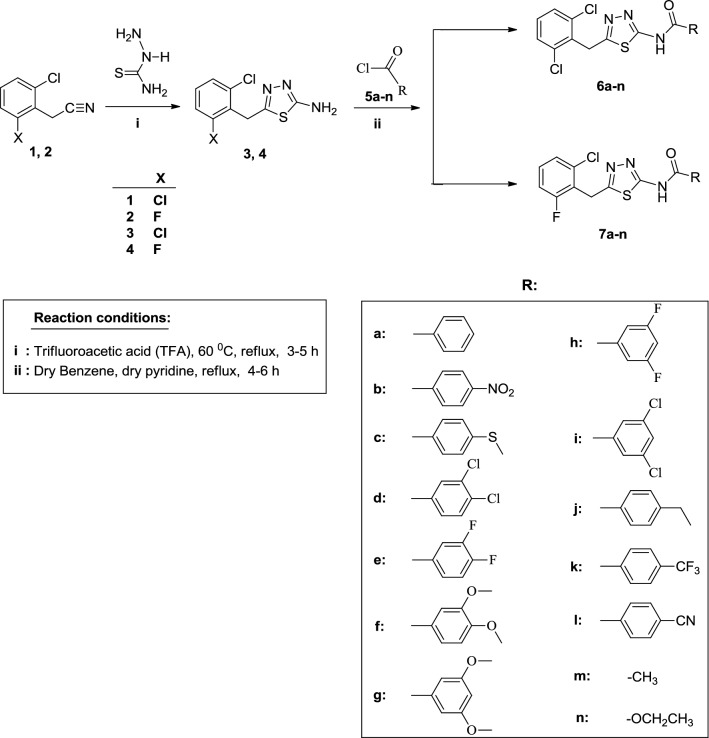
Synthetic route for the synthesis of the target compounds (**6a**–**n**, **7a**–**n**)

**Scheme 2:**
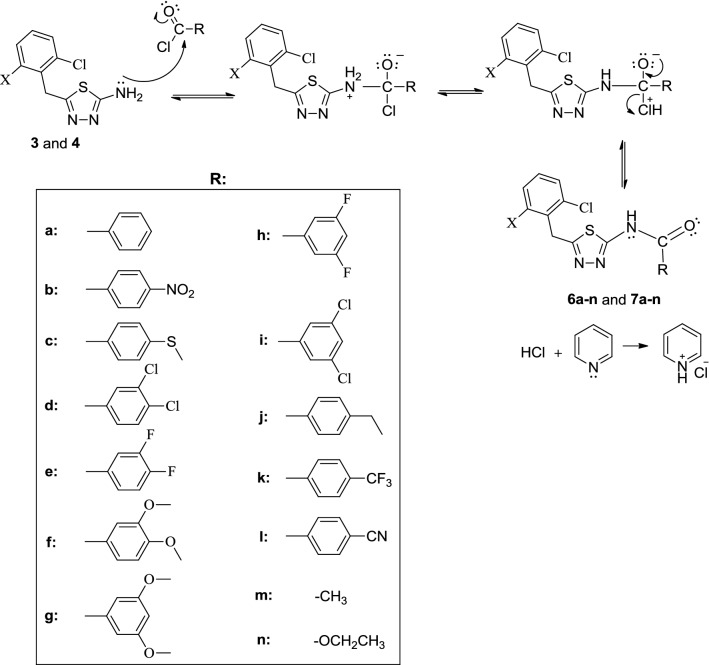
The formation mechanism for the target compounds (**6a**–**n** and **7a**–**n**)

The acyl derivatives of thiadiazole, which are the target compounds of the study, were obtained from the reactions of acyl derivatives (**5a**–**n**) with the compounds **3** and **4**. All the synthesised 28 compounds (**6a**–**n** and **7a**–**n**) were obtained in moderate to good yields (56–87%). The synthetic route employed to synthesise these compounds is given in Scheme 1 and the formation mechanism is shown in Scheme 2.

As can be seen from the reaction mechanism in Scheme 2, the main reaction proceeds through a typical nucleophilic acyl substitution reaction.

The structure of the compounds obtained were elucidated using the FT-IR, ^1^H NMR, ^13^C NMR, elemental analysis and mass spectroscopy techniques. The results are given in detail in “Experimental” section, and the relevant spectra are given in Additional file 1. In addition, the structure of the compound **7n**, obtained as a single crystal, was explained with X-ray spectroscopy.

The crystal structure of the compound **7n** and all X-ray data are provided in Additional file 1.

The target compounds in our study (**6a**–**n** and **7a**–**n**) were synthesised in moderate to high yields (56–87%) from the reaction of the acyl chloride derivatives (**5a**–**n**) with the 2-amino-1,3,4-thiadiazole derivatives (**3** and **4**) in the presence of dry benzene.

In the IR spectra of the compounds **6a**–**n** and **7a**–**n**, the symmetric and asymmetric absorption bands corresponding to –NH_2_ group (3261–3098 cm^−1^) disappear and instead, the –NH absorption bands at 3186–3092 cm^−1^ are observed which are the most significant evidences that the compounds were acylated.

Another significant evidence is the C=O absorption band peaks seen at 1720–1624 cm^−1^. The appearance of the –NH and C=O absorption bands in the IR spectra is another indication that the compounds (**6a**–**n** and **7a**–**n**) were acylated. Other spectrum data of the compounds are presented in detail in “Experimental” section.

Also, when the ^1^H NMR spectrums of these compounds are examined, the disappearance of the –NH_2_ proton signals observed at 7.69 and 7.08 ppm for the compounds **3** and **4** and appearance of –NH signals as a singlet which shift at 13.40–12.09 ppm due to the electron withdrawing property of the carbonyl group, are the most significant evidence that these compounds (**6a**–**n** and **7a**–**n**) were acylated. This data is consistent with findings in the literature [24, 25]. Other ^1^H NMR spectrum data for the compounds are presented in “Experimental” section, and the relevant spectra are given in Additional file 1.

Similarly, when we examine the ^13^C NMR spectra of the target compounds (**6a**–**n** and **7a**–**n**), the appearance of the C=O carbonyl group peaks at 169.03–162.49 ppm also supports that the amino group in the thiadiazole ring was acylated. The C-2 carbon signals corresponding to the thiadiazole ring in the compounds **6a**–**n** and **7a**–**n** were observed in the range 161.12–150.26 ppm, and the peaks corresponding to the C-5 carbon were observed between 169.01 and 161.78 ppm. Other ^13^C NMR spectrum data of the compounds are presented in detail in “Experimental” section.

In addition, the mass spectra of all the synthesised compounds were obtained and the products were also confirmed with the molecular ion peaks.

### In vitro antimicrobial activity studies

The activity values of the compounds against the tested fungus species (inhibition zones and percentage inhibition values) are presented in Tables 1 and 2. At the used doses of the compounds (500 and 1000 µg/ml), varying levels of activity were observed for each fungus species. For all compounds and doses used, the most sensitive fungus species was found to be the *Monillia fructigena* pathogen. This is followed by *Fusarium oxysporum f.* sp. *lycopersici* (FOL) and *Alternaria solani* pathogens. For thiram, which was used for positive control purposes, a 25 mm inhibition zone was observed for all pathogens, and it inhibited their development at 100%. DMSO, which was used in negative control, did not affect the development of pathogens. According to the results obtained, the smallest inhibition zones at 1000 µg/ml for FOL was found in compound **3** (12.35 mm), and the greatest was in compound **7c** (19.51 mm). In case of *M. fructigena*, the smallest inhibition zones was found in compound **4** (14.25 mm), and the greatest was in compound **7g** (21.23 mm); the smallest for *A. solani* was in compound **6l** (10.73 mm), and the greatest in compound **7n** with 18.19 mm. In addition, the percent inhibition values that the compounds exhibit against the pathogens were between 49 and 77% at the 1000 µg/ml dose for FOL, between 61 and 85% for *M. fructigena*, and between 43 and 73% for *A. solani* (Table 2). It is clear that by increasing the doses used, 100% inhibition rates would be observed. The LD_50_, minimum fungicidal activity (MFC) and minimum inhibitory concentration (MIC) values of the compounds against the fungi were calculated (Table 3). Accordingly, the LD_50_ values were calculated to be between 350 and 797 µg/ml for FOL; between 312 and 679 µg/ml for *M. fructigena*, and between 414 and 1392 µg/ml for *A. solani*. Despite the overall variation according to the fungus type, the MFC values varied between > 250 and > 1000 µg/ml, and the MIC values varied between < 31.25 and 500. According to the results, it was found that the compounds exhibited high to moderate levels of activity against the tested organisms.

**Table 1:** Inhibition zones of compounds against plant pathogens

Compounds	Mean zone of inhibition (mm)^a^					
	*Fusarium oxysporum f.* sp. *lycopersici*		*Monilia fructigena*		*Alternaria solani*	
	Doses (µg/ml)		Doses (µg/ml)		Doses (µg/ml)	
	500	1000	500	1000	500	1000
**C−**	0 ± 0.00		0 ± 0.00		0 ± 0.00	
**3**	10.12 ± 0.91	12.35 ± 0.52	14.16 ± 0.80	18.19 ± 0.86	12.45 ± 0.49	14.81 ± 0.56
**4**	14.67 ± 0.93	17.81 ± 1.02	11.19 ± 0.59	14.25 ± 0.54	9.35 ± 0.33	13.18 ± 1.25
**6a**	13.19 ± 0.67	17.08 ± 0.72	13.40 ± 0.23	15.88 ± 0.41	10.76 ± 0.29	13.31 ± 0.84
**6b**	10.92 ± 0.22	15.68 ± 0.97	16.87 ± 0.32	20.08 ± 0.40	10.40 ± 0.42	14.61 ± 0.37
**6c**	13.83 ± 0.30	17.82 ± 0.09	16.31 ± 1.36	18.34 ± 0.34	10.70 ± 0.83	14.73 ± 0.94
**6d**	11.27 ± 0.53	15.33 ± 0.36	14.44 ± 3.38	18.09 ± 1.67	9.18 ± 0.59	11.73 ± 0.34
**6e**	11.11 ± 0.61	15.14 ± 1.02	12.20 ± 0.74	17.17 ± 1.20	14.27 ± 3.18	17.15 ± 1.68
**6f**	13.95 ± 0.58	17.30 ± 0.73	14.45 ± 0.60	18.65 ± 0.51	9.74 ± 0.63	15.21 ± 1.15
**6g**	15.51 ± 0.28	18.53 ± 0.39	16.08 ± 0.19	18.30 ± 0.16	13.44 ± 1.00	16.92 ± 0.12
**6h**	15.25 ± 0.30	19.14 ± 0.40	11.59 ± 0.58	15.13 ± 1.68	9.57 ± 0.30	11.94 ± 0.35
**6i**	10.33 ± 1.29	13.88 ± 1.08	12.37 ± 3.66	17.40 ± 1.21	9.96 ± 0.33	12.56 ± 0.34
**6j**	13.31 ± 0.87	18.80 ± 0.74	15.28 ± 0.85	17.93 ± 0.50	11.37 ± 1.38	15.27 ± 1.29
**6k**	12.47 ± 0.78	16.67 ± 0.94	19.95 ± 1.49	20.52 ± 1.24	11.16 ± 0.20	15.19 ± 0.90
**6l**	13.25 ± 1.59	17.52 ± 0.45	11.44 ± 0.31	15.84 ± 0.39	7.31 ± 0.03	10.73 ± 0.08
**6m**	13.69 ± 1.28	18.69 ± 1.28	11.37 ± 1.22	16.59 ± 1.15	11.10 ± 0.36	14.61 ± 0.56
**6n**	14.36 ± 0.44	17.76 ± 0.16	12.71 ± 0.52	18.23 ± 0.58	12.05 ± 0.29	15.96 ± 0.16
**7a**	13.48 ± 1.07	18.51 ± 1.71	14.31 ± 0.33	17.99 ± 0.29	11.29 ± 0.25	14.01 ± 1.11
**7b**	13.40 ± 1.39	17.98 ± 0.58	16.83 ± 1.89	20.04 ± 0.95	10.67 ± 0.48	13.73 ± 0.89
**7c**	15.71 ± 1.25	19.51 ± 0.78	15.34 ± 0.27	17.64 ± 0.38	10.18 ± 0.59	14.10 ± 1.64
**7d**	11.39 ± 0.04	14.80 ± 0.45	14.95 ± 0.32	17.72 ± 0.23	10.69 ± 0.64	12.57 ± 0.63
**7e**	13.48 ± 0.30	17.33 ± 0.52	10.73 ± 0.51	16.83 ± 0.36	10.90 ± 0.32	16.24 ± 0.26
**7f**	13.31 ± 0.31	16.56 ± 0.61	12.42 ± 1.43	16.54 ± 0.79	8.02 ± 0.60	10.88 ± 0.37
**7g**	12.22 ± 0.29	15.91 ± 0.63	11.90 ± 0.71	21.23 ± 0.97	12.19 ± 0.52	15.88 ± 1.13
**7h**	14.68 ± 0.94	18.13 ± 0.46	11.65 ± 0.89	17.34 ± 0.78	7.72 ± 0.44	10.87 ± 0.44
**7i**	12.57 ± 0.41	15.81 ± 0.51	14.84 ± 0.23	17.67 ± 0.11	9.04 ± 0.46	11.01 ± 0.55
**7j**	11.19 ± 0.95	16.40 ± 0.55	11.77 ± 0.86	20.16 ± 1.81	9.94 ± 0.26	15.38 ± 0.05
**7k**	11.97 ± 1.30	15.90 ± 1.00	12.61 ± 2.09	16.66 ± 0.94	8.92 ± 1.22	11.38 ± 0.49
**7l**	13.55 ± 0.37	16.38 ± 0.31	9.36 ± 0.92	17.36 ± 0.24	9.90 ± 0.21	12.71 ± 0.79
**7m**	13.59 ± 1.88	16.82 ± 0.14	10.17 ± 1.15	16.06 ± 1.46	6.86 ± 0.63	11.54 ± 0.41
**7n**	13.65 ± 0.69	17.54 ± 0.52	17.31 ± 1.02	18.74 ± 0.31	12.50 ± 0.45	18.19 ± 0.84
**C+**	25.00 ± 1.32		25.00 ± 0.98		25.00 ± 0.53	

Inhibition zone (IZ) ± standard deviation (SD); C+, positive control (Thiram); C−, negative control (DMSO)

^a^Mean of three assays

**Table 2:** Percentage inhibition of compounds against test fungi (%)

Compounds	FOL		MF		AS	
	Doses (µg/ml)		Doses (µg/ml)		Doses (µg/ml)	
	500	1000	500	500	1000	500
**C−**	–	–	–	–	–	–
**3**	40	49	57	73	50	59
**4**	59	71	45	57	37	53
**6a**	53	68	54	64	43	53
**6b**	44	63	67	80	42	58
**6c**	55	71	65	73	43	59
**6d**	45	61	58	72	37	47
**6e**	44	61	49	69	57	69
**6f**	56	69	58	75	39	61
**6g**	62	74	64	73	54	68
**6h**	61	77	46	61	38	48
**6i**	41	56	49	70	40	50
**6j**	53	75	61	72	45	61
**6k**	50	67	80	82	45	61
**6l**	53	70	46	63	29	43
**6m**	55	75	45	66	44	58
**6n**	57	71	51	73	48	64
**7a**	54	74	57	72	45	56
**7b**	54	72	67	80	43	55
**7c**	63	78	61	71	41	56
**7d**	46	59	60	71	43	50
**7e**	54	69	43	67	44	65
**7f**	53	66	50	66	32	44
**7g**	49	64	48	85	49	64
**7h**	59	73	47	69	31	43
**7i**	50	63	59	71	36	44
**7j**	45	66	47	81	40	62
**7k**	48	64	50	67	36	46
**7l**	54	66	37	69	40	51
**7m**	54	67	41	64	27	46
**7n**	55	70	69	75	50	73
**C+**	100	100	100	100	100	100

(−), no percentage inhibition; FOL, *Fusarium oxysporum f.* sp. *lycopersici*; MF, *Monilia fructigena*; AS, *Alternaria solani*

**Table 3:** Antifungal activity values (LD_50_, MFC and MIC) of compounds against test fungi

Compounds	(LD_50_/MFC/MIC µg/ml)		
	FOL	MF	AS
**3**	797/> 1000/> 125	483/> 500/< 31.25	612/> 1000/62.5
**4**	412/> 500/62.5	638/> 1000/> 250	771/> 1000/62.5
**6a**	534/> 1000/125	564/> 1000/250	737/> 1000/125
**6b**	568/> 1000/> 250	366/> 250/< 31.25	634/> 1000/62.5
**6c**	505/> 500/62.5	457/> 500/31.25	658/> 1000/62.5
**6d**	642/> 1000/250	476/> 500/< 31.25	961/> 1000/> 250
**6e**	603/> 1000/250	473/> 500/< 31.25	414/> 500/< 31.25
**6f**	508/> 500/250	468/> 500/< 31.25	673/> 1000/125
**6g**	474/> 500/125	470/> 500/< 31.25	546/> 500/125
**6h**	490/> 500/125	679/> 1000/250	929/> 1000/> 250
**6i**	750/> 1000/< 500	563/> 1000/125	830/> 1000/> 250
**6j**	520/> 500/62.5	502/1000/125	656/> 1000/125
**6k**	499/> 500/62.5	312/> 250/< 31.25	597/> 1000/125
**6l**	441/> 500/125	539/> 1000/< 125	1392/> 1000/< 500
**6m**	406/> 500/> 31.25	512/> 500/62.5	614/> 1000/125
**6n**	404/> 500/> 31.25	421/> 500/31.25	509/1000/62.5
**7a**	406/> 500/62.5	412/> 500/31.25	638/> 1000/62.5
**7b**	432/> 500/31.25	320/> 250/< 31.25	691/> 1000/> 31.25
**7c**	350/> 250/< 31.25	395/<500/< 31.25	679/> 1000/> 31.25
**7d**	597/> 1000/125	394/> 500/< 31.25	788/> 1000/< 62.5
**7e**	434/> 500/250	519/> 500/125	546/> 500/> 31.25
**7f**	453/> 500/31.25	485/> 500/125	1308/> 1000/< 500
**7g**	509/> 500/250	371/> 500/< 31.25	519/> 500/31.25
**7h**	398/> 250/< 31.25	480/> 500/125	1361/> 1000/< 500
**7i**	522/> 500/> 31.25	406/> 500/31.25	1107/> 1000/250
**7j**	564/> 1000/125	405/> 500/31.25	637/> 1000/62.5
**7k**	519/> 500/125	488/> 500/> 125	1091/> 1000/250
**7l**	453/> 500/62.5	546/> 1000/62.5	809/> 1000/125
**7m**	443/> 500/> 31.25	572/> 1000/62.5	1313/> 1000/250
**7n**	418/> 500/> 31.25	343/> 500/< 31.25	486/> 500/61.25
**C+**	596/< 3000/> 31.25	717 < 3000/< 62.5	565 <3000/< 31.25

LD_50_, the amount of a substance, which causes the death of 50% (one half) of test fungi; MFC, minimum fungicidal concentration; MIC, minimum inhibitory concentration; C+, positive control (Thiram 80%)

### Computational studies

#### Identification of target protein

Molecular docking is a value added tool in computer aided drug design. It helps us to understand inhibition mechanism of a drug or drug candidate against its target. Ligand similarity search is one of the techniques used for target prediction. This method compares structures of the studied compounds to the compounds with known targets in the databases. For the cases where experimental crystal structures are not available, homology modelling is used to build protein structure based on a template. Optimization or refinement of protein structures are done through molecular dynamics (MD) simulations.

Here we followed a multi-stage computational strategy in order to find a potential target. Initially, the most active two structures based on their LD_50_ values for each fungus (*Alternaria solani*; **6e**, **7n**. *Monilia fructigena*; **6k**, **7b**. *Fusarium oxysporum f.* sp. *lycopersici*; **7c**, **7h**) were selected and used for the similarity search. A number of similar compounds corresponding to our structures was retrieved from NCBI’s PubChem database. The resulting structures and their targets are listed in Table 4.

**Table 4:** Similar structures obtained by similarity search through PubChem database (Tanimoto threshold ≥ 90%)

Structure (PubChem CID)	Target gene (Uniprot ID)	Organism	Activity (µM)	BioAssay AID
1103113	SRC (P12931)	*Homo sapiens*	1.3	485313
2873325	NS3 Helicase (P29846)	*Hepatitis C Virus*	Active	720488
1405531	Grm8 (P70579)	*Rattus norvegicus*	Active	488969
2363161	SRC (P12931)	*Homo sapiens*	0.9	317425
2363161	ABL1 (P00519)	*Homo sapiens*	0.406	317426
2192320	SRC (P12931)	*Homo sapiens*	0.718	317425
2192320	ABL1 (P00519)	*Homo sapiens*	0.272	317426
4380426	NPC1 (O15118)	*Homo sapiens*	3.1623	485313
4380426	RAB9A (P51151)	*Homo sapiens*	3.9811	485297
4380426	Smarca4 (Q3TKT4)	*Mus musculus*	Active	602393
4380426	MDM4 (O15151)	*Homo sapiens*	Active	485346
4380426	MDM2 (Q00987)	*Homo sapiens*	Active	485346
3787336	PTBP1 (P26599)	*Homo sapiens*	Active	2417
3787336	Cysteine protease (A6XG55)	*Trypanosoma cruzi*	25.1189	1476
878365	SENP6 (Q9GZR1)	*Homo sapiens*	Active	2599
878365	SENP7 (Q9BQF6)	*Homo sapiens*	16.8	488904
878365	CASP3 (P42574)	*Homo sapiens*	18.9	488901
3669135	Rorc (P51450)	*Mus musculus*	17.7828	2551
3669135	SENP7 (Q9BQF6)	*Homo sapiens*	Active	434973
3669135	PAFAH1B2 (P68402)	*Homo sapiens*	Active	492953
3669135	MC4R (P32245)	*Homo sapiens*	Active	540308
753766	FAK1 (Q05397)	*Homo sapiens*	11.87	657571
4420955	FAK1 (Q05397)	*Homo sapiens*	12.68	657571

**Fig. 1:**
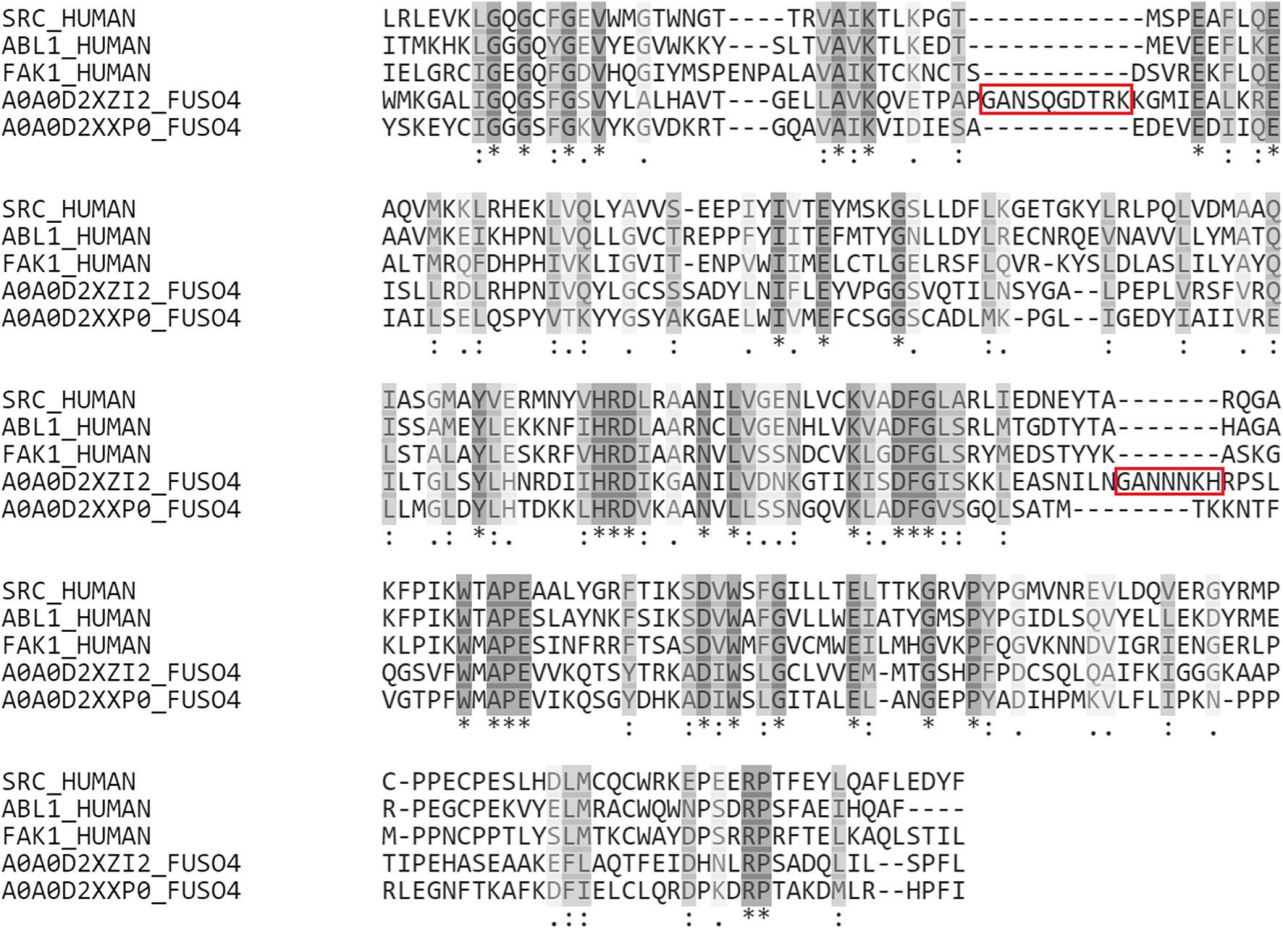
Multiple sequence alignment results of protein kinase domain of SRC, ABL1, FAK1, and FOL’s proteins

Proteins belonging to the most active compounds in Table 4 were selected for the BLAST search (SRC and ABL1). These proteins are members of non-receptor protein tyrosine kinases family. Besides, FAK1 in Table 4 is also a member of this protein family. On the other hand, some of protein kinases have been shown to be antifungal targets [26, 27]. As seen in Fig. 1, a highly conserved kinase domain is present in those three proteins. This region contains a ligand binding site targeted to design anticancer drugs in human, and many protein structures of this domain are available in the Protein Data Bank (PDB) [28–30]. Thus, these proteins (SRC, ABL1 and FAK1) were selected for further modelling of the target of our compounds.

As a first step, the amino acid sequences of SRC, ABL1 and FAK1 were retrieved from the Universal Protein Resource (UniProt) and then submitted to the BLAST search to find similar protein sequences present in our target organisms [31–33]. Although the BLAST is available for only two species, *Fusarium oxysporum f.* sp. *lycopersici* (FOL) and *Alternaria solani* (AS), the size of proteome information for AS is not adequate. Thus, the BLAST search was performed for only FOL to identify similar protein sequences (Table 5).

**Table 5:** Alignment results from the BLAST search

Query (UniProt ID)	Subject (UniProt ID)	%Identity	Alignment length	Mismatches	Gaps	E-value	Score
SRC (P12931)	STE/STE11 (A0A0D2XZI2)	28.030	264	161	8	3.14e−23	104
ABL1 (P00519)	STE/STE20/YSK (A0A0D2XXP0)	28.571	245	166	6	3.52e−26	115
FAK1 (Q05397)	STE/STE20/YSK (A0A0D2XXP0)	28.571	336	193	11	1.81e−28	123

Finally, two different proteins were identified (Table 5). A remarkable alignment with 33 identical and 61 similar positions was obtained at the protein kinase domain (Fig. 1). After the comparison of those two FOL’s proteins with human proteins, A0A0D2XXP0 was found to be more similar to human proteins than A0A0D2XZI2. Besides, additional insertion sites were observed in the A0A0D2XZI2 which can cause a different conformational change at the tertiary structure, and also affect the ligand binding site (Fig. 1). Hence, A0A0D2XXP0 was selected as the potential target protein of our chemical structures.

#### Homology modelling

The 3D structure of STE/STE20/YSK protein kinase is currently not available in the Protein Data Bank (PDB). In such cases, homology modelling has been found as an effective method for 3D structure prediction of proteins. Therefore, homology modelling was performed through the Automated Comparative Protein Modelling Server (SWISS-MODEL) [34]. The STE/STE20/YSK protein kinase sequence was retrieved from Uniprot (Uniprot ID: A0A0D2XXP0). A sequence similarity search against other sequences with available structural information in PBD was applied to determine the template structure. A high resolution (1.6 Å) crystal structure of Serine/threonine-protein kinase 24 (MST3) (PDB ID: 4U8Z) was selected as template which shows 66.93% sequence identity (GMQE: 0.80) with the target.

**Fig. 2:**
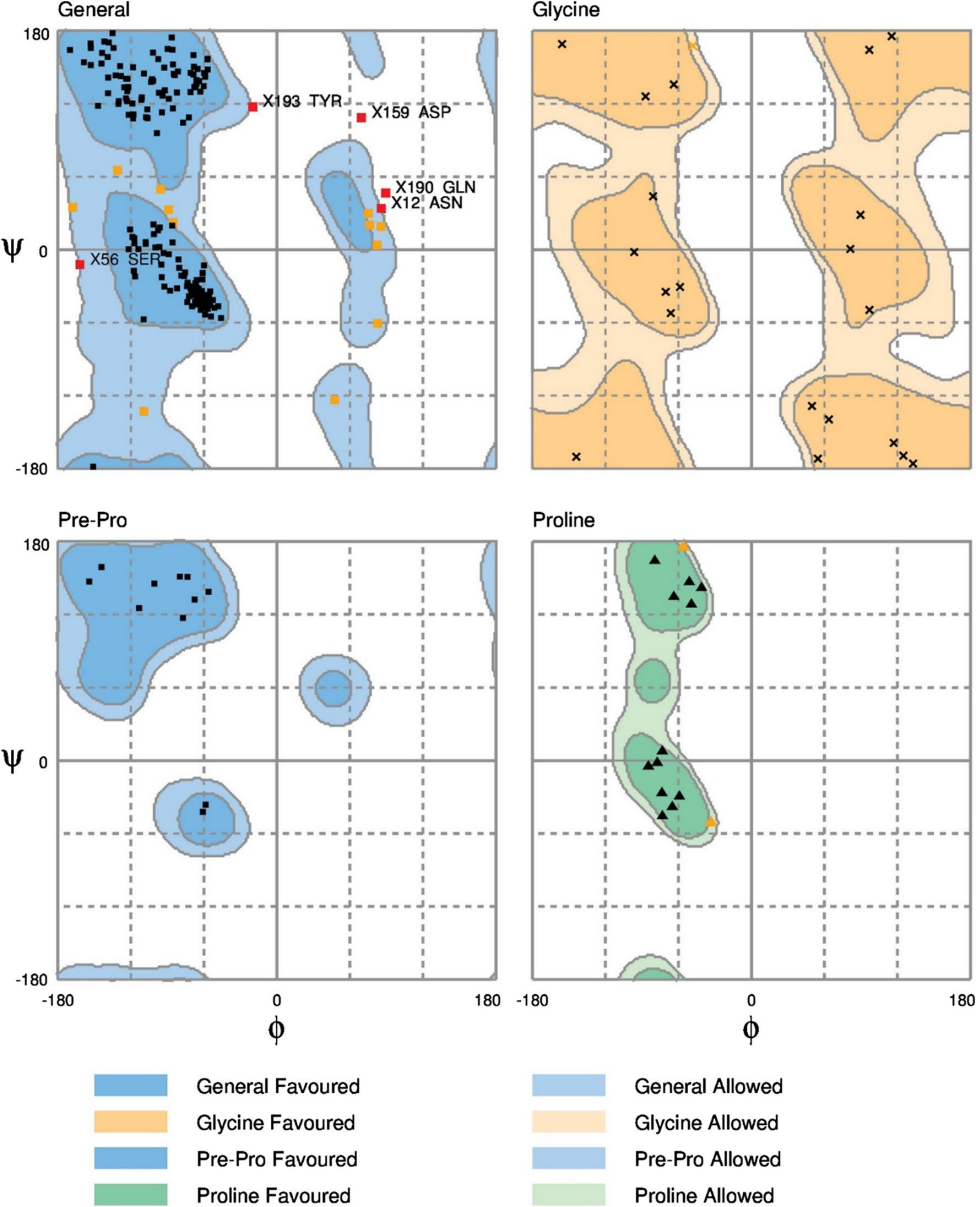
The Ramachandran’s plot of the STE/STE20/YSK protein kinase model computed with the RAMPAGE server

**Fig. 3:**
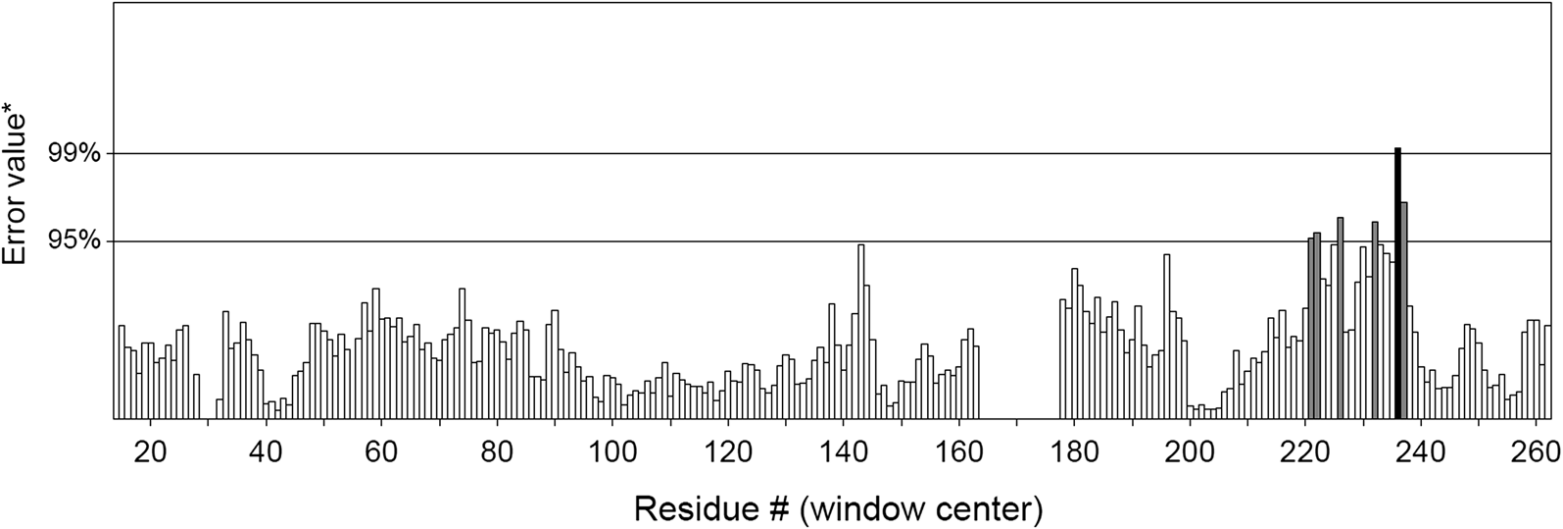
ERRAT plot for measurement of the structural errors

The backbone of the model was validated using Ramachandran plot obtained through RAMPAGE server [35]. The Ramachandran plot for our model structure indicated that 92.1% of the residues were located in the most favourable region, 5.9% of the residues were in the allowed regions, and 2.0% of the residues were in the outlier regions. This suggests that the STE/STE20/YSK protein kinase model is of good stereo chemical quality (Fig. 2). The measurement of the structural error at each amino acid residue in the 3D structural model was measured by the ERRAT plot [36]. The overall quality factor of the model was computed as 97.38% (Fig. 3).

#### Molecular dynamic simulation

**Fig. 4:**
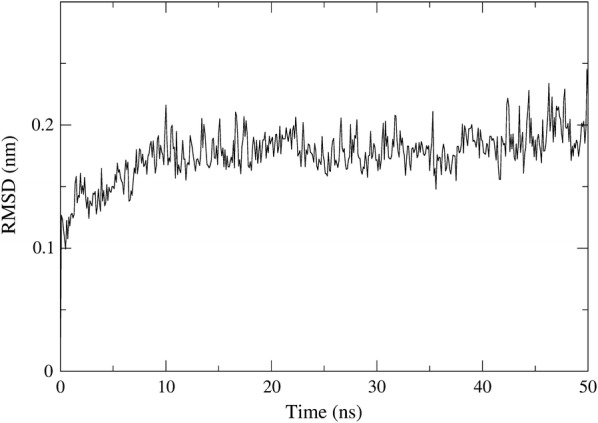
RMSD values of protein backbone of STE/STE20/YSK protein kinase homology model during 50 ns MD simulation

The validated protein model was used in the molecular dynamics simulations. Root-mean-square-deviation (RMSD) and radius of gyration (Rg) were used to check the stability of protein. The RMSD is a crucial parameter to analyse the stability of MD trajectories. To check the stability of protein during the simulation, RMSD of the protein backbone atoms were plotted as a function of time (Fig. 4). The analysis of the RMSD values indicates that the equilibration was reached after 7 ns simulation time.

The radius of gyration, Rg, was also carried out to give us insight into the overall dimensions of the protein. Hence this analysis gives us the overall dimensions of the protein. The calculated Rg values over the simulation time scale for the STE/STE20/YSK protein are shown in Additional file 1. The Rg values were stabilized at about 5 ns, indicating that the MD simulation achieved equilibrium after 5 ns. The structure obtained at the end of the simulation was taken as the stabilized structure for further studies in molecular docking.

#### Molecular docking

Prior to molecular docking of the synthesized thiadiazole derivatives to STE/STE20/YSK protein kinase using our homology-modelled protein, the reliability and accuracy of LeDock was analysed using crystal structures of MST3 which has the best sequence identity with STE/STE20/YSK protein kinase. Accordingly, 17 PDB structures of MST3 were downloaded from PDB database. Then, crystal binding poses, and binding affinities of native ligands were predicted using LeDock in triplicate. LeDock scores and RMSD values are listed in Table 6. According to the results, LeDock displayed 0.74 ± 0.02 pearson correlation (score vs IC_50_) and predicted the experimental binding mode with 78.43 ± 3.40% (RMSD) success rate. These results suggested that LeDock can be used as a reliable docking tool for the STE/STE20/YSK protein kinase.

**Table 6:** Assessment of LeDock using 17 crystal structures of MST3 which have the best sequence identity with our target

PDB ID	Ligand	PUBCHEM CID	IC_50_ (µM)	LeDock (kcal/mol)	RMSD (Å)
4QMN	Bosutinib	5328940	0.003	− 9.36 ± 0.03	2.20 ± 0.38
4QMY	Staurosporine	44259	0.004	− 8.86 ± 0.06	0.86 ± 0.01
4QMT	Hesperadin	10142586	0.01	− 9.86 ± 0.04	1.20 ± 0.17
4W8E	PF-06645342	91623338	0.0121	− 8.39 ± 0.03	1.79 ± 0.18
4QMP	CDK1/2_Inhibitor_III	5330812	0.014	− 9.28 ± 0.03	1.76 ± 0.12
4QMO	PKR_Inhibitor_C16	67016828	0.019	− 7.64 ± 0.02	0.23 ± 0.04
4QMV	PF-03814735	51346455	0.023	− 9.53 ± 0.07	0.67 ± 0.02
4QMW	PP-121	24905142	0.086	− 8.34 ± 0.01	0.42 ± 0.06
4QO9	Danusertib	11442891	0.16	− 7.86 ± 0.01	0.41 ± 0.07
4U8Z	PF-06447475	72706840	0.178	− 7.62 ± 0.02	0.33 ± 0.03
4QMZ	Sunitinib	5329102	0.21	− 8.54 ± 0.05	2.49 ± 0.05
4QMQ	CP-673451	10158940	0.26	− 8.98 ± 0.02	0.95 ± 0.04
4QMM	AT-9283	11696609	0.46	− 8.53 ± 0.05	1.22 ± 0.25
4QMU	JNJ-7706621	5330790	1.3	− 8.08 ± 0.02	1.20 ± 0.77
4QMS	Dasatinib	3062316	7.4	− 8.83 ± 0.41	3.86 ± 1.63
4QMX	Saracatinib	10302451	11	− 7.50 ± 0.05	1.23 ± 0.11
4QNA	Tp-Fragment	6806574	23	− 5.50 ± 0.06	5.58 ± 0.07
				0.74 ± 0.02*	78.43 ± 3.40**

* Pearson correlation (*r*) value calculated using the docking scores and IC_50_ values

** Percent of success rate for pose prediction within the best-scored pose

Finally, we performed molecular docking to our studied compounds using stabilized structure of STE/STE20/YSK protein kinase model. Each docking was carried out in triplicate. The results are listed in Table 7. Ligands are ranked according to the LD_50_ values.

**Table 7:** Docking scores, LD_50_ values and calculated molecular properties of the studied compounds

Comp.	LD_50_	LeDock (kcal/mol)	SASA^b^	QPlogPo/w^c^	QPlogBB^d^	QPPMDCK^e^	%HOA^f^	PSA^g^	RoF^h^
**7c**	350.00	− 7.2 ± 0.06	639.88	4.27	− 0.45	3068.46	100.00	65.45	+
**7h**	398.00	− 6.04 ± 0.05	601.82	4.15	− 0.25	5391.60	100.00	65.63	+
**6n**	404.00	− 6.15 ± 0.03	558.23	3.08	− 0.58	1419.32	95.35	76.58	+
**6m**	406.00	− 5.64 ± 0.02	506.57	2.56	− 0.36	1896.76	94.11	68.84	+
**7a**	406.00	− 5.77 ± 0.06	596.58	3.68	− 0.46	1942.04	100.00	67.88	+
**4**	412.00	− 5 ± 0.02	419.66	2.08	− 0.30	1654.09	90.79	55.23	+
**7n**	418.00	− 6.01 ± 0.10	548.66	2.93	− 0.66	1012.14	93.95	77.23	+
**7b**	432.00	− 6.59 ± 0.07	628.20	2.68	− 1.74	150.18	79.02	112.17	+
**7e**	434.00	− 6.32 ± 0.03	609.68	4.24	− 0.23	6111.44	100.00	66.82	+
**6l**	441.00	− 6.72 ± 0.04	633.75	3.09	− 1.29	378.36	86.03	92.20	+
**7m**	443.00	− 5.82 ± 0.02	503.14	2.58	− 0.24	2684.89	96.42	67.17	+
**7f** ^**a**^	453.00	− 6.38 ± 0.06	NA	NA	NA	NA	NA	NA	NA
**7l**	453.00	− 6.66 ± 0.08	626.86	3.00	− 1.23	395.77	87.00	93.13	+
**6g**	474.00	− 6.42 ± 0.02	670.74	4.19	− 0.50	2889.36	100.00	83.43	+
**6h**	490.00	− 6.02 ± 0.07	610.56	4.25	− 0.17	5544.37	100.00	67.79	+
**6k**	499.00	− 6.69 ± 0.11	696.71	5.10	− 0.50	6026.31	94.22	66.34	−
**6c**	505.00	− 6.9 ± 0.07	648.58	4.44	− 0.44	3600.07	100.00	66.28	+
**6f**	508.00	− 6.47 ± 0.07	680.55	4.40	− 0.39	4240.46	100.00	82.20	+
**7g**	509.00	− 6.37 ± 0.08	664.51	3.92	− 0.70	1552.70	100.00	83.28	+
**7k**	519.00	− 7.17 ± 0.17	639.11	4.70	− 0.24	7186.32	100.00	65.83	+
**6j**	520.00	− 6.34 ± 0.09	644.03	4.61	− 0.38	3203.37	100.00	66.79	+
**7i**	522.00	− 6.18 ± 0.04	629.94	4.56	− 0.10	8066.57	100.00	68.85	+
**6a**	534.00	− 6.26 ± 0.06	590.06	4.01	− 0.28	3046.60	100.00	66.24	+
**7j**	564.00	− 6.32 ± 0.06	654.68	4.51	− 0.51	2340.98	100.00	67.50	+
**6b**	568.00	− 6.36 ± 0.06	640.43	3.03	− 1.57	273.80	82.57	114.02	+
**7d**	597.00	− 6.19 ± 0.07	632.05	4.56	− 0.14	7085.16	100.00	69.02	+
**6e**	603.00	− 6.69 ± 0.04	613.97	4.35	− 0.24	6419.14	100.00	66.02	+
**6d**	642.00	− 6.27 ± 0.10	640.40	4.71	− 0.15	7874.53	100.00	68.76	+
**6i**	750.00	− 6.63 ± 0.10	645.84	5.00	− 0.06	10,000.00	100.00	68.25	+
**3**	797.00	− 5.05 ± 0.09	431.02	2.24	− 0.28	1930.57	91.43	55.58	+
Pearson *r* 0.63 ± 0.03									

^a^ADMET values could not be calculated due to the steric clashes between methoxy substituents

^b^Total solvent accessible surface area (SASA) in square angstroms using a probe with a 1.4 Å radius (recommended value: 300.0–1000.0)

^c^Logarithm of the partition coefficient of the compound between *n*-octanol and water (recommended value < 5)

^d^Predicted brain/blood partition coefficient (recommended value: − 3.0 to 1.2)

^e^Predicted apparent MDCK cell permeability in nm/sec (< 25 poor, > 500 great)

^f^Percentage of human oral absorption (< 25% is weak and > 80% is strong)

^g^Polar surface area (recommended value ≤ 140 Å^2^) [40]

^h^Violations to the Lipinski’s rule of five [41]

The Pearson correlation was also calculated. Although the scoring functions of the docking software available at the market have low success rate in discriminating between active and inactive compounds [24, 37–39] the Pearson correlation was found to be 0.63 ± 0.03 which shows a good agreement between experimental LD50 values and calculated docking scores.

**Fig. 5:**
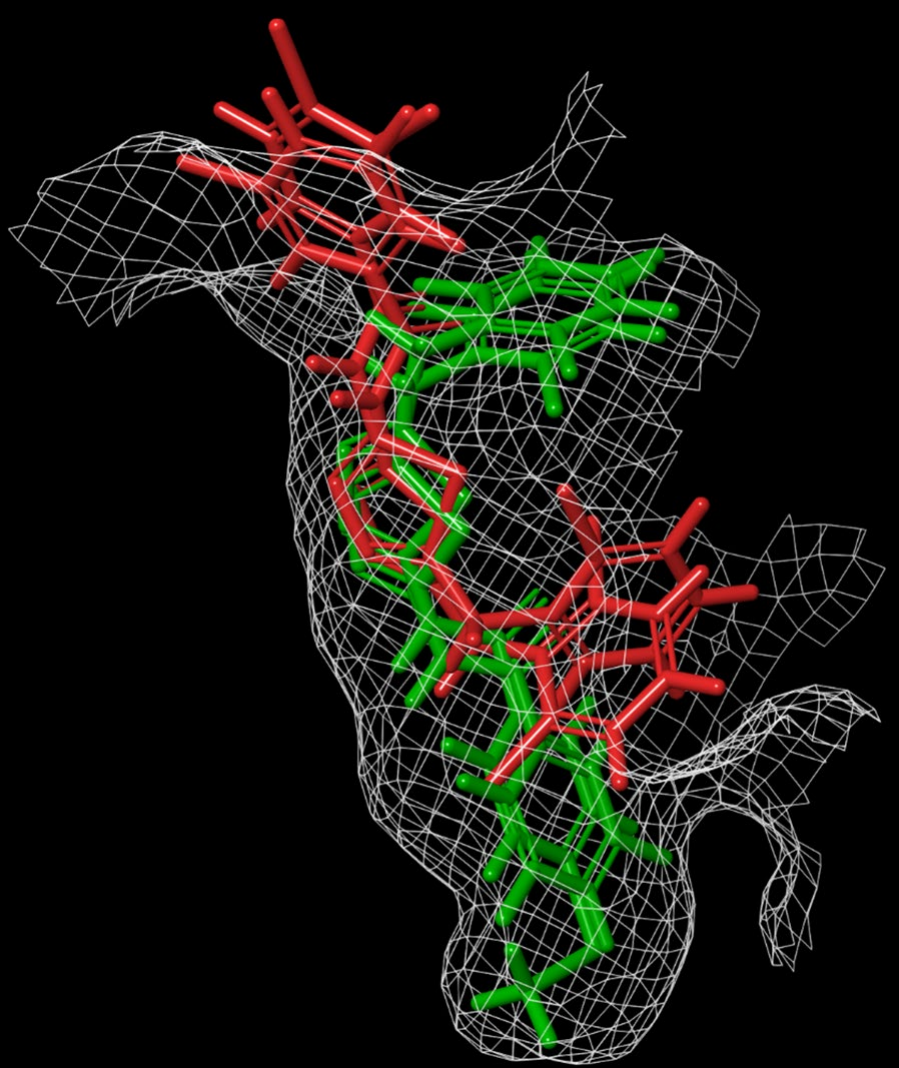
Alignment of the binding conformations of the compounds **7c**, **7h** (green) and **6d**, **6i** (red). (Figure generated using Schrodinger’s Maestro module) [42]

In an effort to investigate the differences between the binding modes of the active and non-active compounds, we aligned the most two active compounds (**7c** and **7h**) and also the least two activate compounds (**6d** and **6i**) which share similar scaffolds. As can clearly be seen in Fig. 5, the most active compounds (**7c** and **7h**) adopt similar binding orientations and burry deep into the binding pocket. The conformational changes in the least active compounds (**6d** and **6i**) prevent them to fit into the binding site which we believe results in poor binding interaction with the amino acid residues of the STE/STE20/YSK protein kinase of FOL.

Finally, as can be seen from Table 7, practically all compounds obey Lipinski’s rule of five and all have drug-like pharmacokinetic profile.

## Conclusion

In the present study, 2-amino-1,3,4-thiadiazole and its acyl derivatives were synthesised with moderate to high yields using simple and applicable methods. The structures of all synthesized compounds were characterised by various spectroscopic methods such as IR, ^1^H NMR, ^13^C NMR, MS.

The in vitro antifungal activity of the synthesised compounds was also evaluated against plant pathogens which revealed promising activities against all tested pathogens.

The combination of several computational tools such as similarity search, homology modelling, molecular dynamics and molecular docking helped in finding a potential target, constructing its 3D model and finally enlighten a possible inhibition mechanism.

In the light of in vitro and in silico results, the studied compounds promise as antifungal candidates worthy of further development in the future.

## Experimental

### Materials and methods

The ^1^H NMR and ^13^C NMR spectra of the compounds were recorded in DMSO-d_6_ using an Agilent NMR VNMRS spectrometer at 400 MHz and 100 MHz, respectively. TMS was used as an internal standard. The IR spectra were measured in ATR using a Perkin Elmer FT-IR Spectrometer Frontier. The mass spectra were measured with a Thermo TSQ Quantum Access Max LC–MS/MS spectrometer. The elemental analysis of the compounds was performed using a LECO 932 CHNS device and the results were within ± 0.4% of the theoretical values. Melting points were recorded on a Thermo Scientific IA9000 series apparatus and were uncorrected. All of the chemicals were obtained from Sigma-Aldrich Chemicals.

### General synthesis of 2-amino-1,3,4-thiadiazole derivatives (**3**,**4**)

In a round-bottomed flask, compounds **1** or **2** (0.075 mol) and thiosemicarbazide (0.100 mol) in trifluoroacetic acid (5 ml) at 60 °C were stirred for 3–5 h. After completion of the reaction, the reaction mixture was poured into 250 ml ice-water mixture and neutralized with diluted ammonia. The solution was filtered, and solid substance was obtained. The solid substance was washed with water, ethyl alcohol, and diethyl ether, respectively. The solid was recrystallized from the appropriate solvent. The pure substance is dried with P_2_O_5_ vacuum oven. Finally, the structures of the synthesized compounds were elucidated with FT-IR, ^1^H NMR, ^13^C NMR, mass spectroscopy, and elemental analysis. The spectral data and the physical properties of the products are listed below.

#### 5-(2,6-Dichlorobenzyl)-1,3,4-thiadiazol-2-amine (**3**)

White solid, yield: 15.79 g (81%), m.p. 219–220 °C (DMF-EtOH, 1:5). IR (ATR, cm^−1^): 3258–3098 (–NH_2_), 3080 (Ar–CH), 2976 (Aliphatic CH), 1582 (C=N). ^1^H NMR (400 MHz, DMSO-d_6_) δ (ppm): 4.40 (s, 2H, –CH_2_), Arom-H [7.49 (d, *J* = 7.8 Hz, 2H), 7.36 (t, *J* = 7.8 Hz, 1H)], 7.69 (bs, 2H, NH_2_). ^13^C NMR (400 MHz, DMSO-d_6_, δ ppm): 32.16 (–CH_2_), Arom-C [129.09 (CH), 130.49 (CH), 133.40 (C), 135.44 (C)], Thiadiazole-C [154.37 (C), 169.39 (C)]. *Anal.* Calcd. for C_9_H_7_Cl_2_N_3_S: C, 41.55; H, 2.71; N, 16.15. Found: C, 41.43; H, 2.76; N, 15.99. MS: *m/z* 260 (M^+^, 74); 261 (M + 1, 51).

#### 5-(2-Chloro-6-fluorobenzyl)-1,3,4-thiadiazole-2-amine (**4**)

White solid, yield: 15.35 g (84%), m.p. 212–213 °C (DMF-EtOH, 1:6). IR (ATR, cm^−1^): 3261–3106 (–NH_2_), 3067 (Ar–CH), 2968 (Aliphatic CH), 1597 (C=N). ^1^H NMR (400 MHz, DMSO-d_6_) δ (ppm): 4.25 (s, 2H, –CH_2_), Arom-H [7.35 (d, *J* = 12.0 Hz, 2H), 7.25 (t, *J* = 7.8 Hz, 1H)], 7.08 (bs, 2H, NH_2_). ^13^C NMR (400 MHz, DMSO-d_6_, δ ppm): 27.34 (–CH_2_), Arom-C [115.12 (CH), 123.98 (CH), 125.98 (C), 130.44 (C), 134.80 (CH), 160.02 (C)], Thiadiazole-C [154.52 (C), 169.21 (C)]. *Anal.* Calcd. for C_9_H_7_ClFN_3_S: C, 44.36; H, 2.90; N, 17.24. Found: C, 44.43; H, 2.87; N, 17.17. MS: *m/z* 243.73 (M^+^, 100).

#### General acylation reactions of 2-amino-1,3,4-thiadiazole derivatives (**6a**–**n**, **7a**–**n**)

In a two-necked flask, compounds **3** or **4** (0.004 mol) were solved in dry benzene (40 ml) and added pyridine (1 ml) to this solution. Acyl chloride derivatives (**5a**–**n**) (0.004 mol) were added drop-wise to this solution at room temperature with the assistance of a dropping funnel. The mixture was then refluxed and stirred for 4–6 h. The progress of the reaction was monitored by TLC at appropriate time intervals. After completion of the reaction, the solution was filtered, and the solid matter was obtained. It was washed with deionized water, ethanol and diethyl ether, respectively. The solid matter was recrystallized from the appropriate solvent. All physical properties and spectral data derived from the obtained products are given below.

#### *N*-(5-(2,6-Dichlorobenzyl)-1,3,4-thiadiazol-2-yl)benzamide (**6a**)

White solid, yield: 1.14 g (78%), m.p. 279–280 °C (DMF-EtOH, 1:1). IR (ATR, cm^−1^): 3169 (–NH–), 3061 (Ar–CH), 2983 (Aliphatic CH), 1667 (C=O), 1582 (C=N). ^1^H NMR (400 MHz, DMSO-d_6_) δ (ppm): 4.62 (s, 2H, –CH_2_), Arom-H [8.06 (d, *J* = 6.4 Hz, 2H), 7.61 (d, *J* = 6.0 Hz, 1H), 7.54 (bs, 2H), 7.52 (bs, 2H), 7.39 (t, *J* = 7.6 Hz, 1H)], 12.99 (s, 1H, NH). ^13^C NMR (400 MHz, DMSO-d_6_, δ ppm): 31.80 (–CH_2_), Arom-C [128.80 (CH), 129.08 (CH), 129.22 (CH), 130.64 (CH), 131.90 (CH), 133.41 (C), 134.64 (C), 135.52 (C)], Thiadiazole-C [159.86 (C), 160.84 (C)], 165.53 C=O. *Anal.* Calcd. for C_16_H_11_Cl_2_N_3_OS: C, 52.76; H, 3.04; N, 11.54. Found: C, 52.68; H, 3.00; N, 11.41. MS: *m/z*: 363.68 (M-1, 100), 365.85 (M + 1, 84).

#### *N*-(5-(2,6-Dichlorobenzyl)-1,3,4-thiadiazol-2-yl)-4-nitrobenzamide (**6b**)

White solid, yield: 1.21 g (74%), m.p. 320–321 °C (DMF-EtOH, 1:15). IR (ATR, cm^−1^): 3133 (–NH–), 3044 (Ar–CH), 2930 (Aliphatic CH), 1677 (C=O), 1596 (C=N). ^1^H NMR (400 MHz, DMSO-d_6_) δ (ppm): 4.62 (s, 2H, –CH_2_), Arom-H [8.29 (d, 4H), 7.53 (bs, 2H), 7.39 (bs, 1H)], 13.40 (s, 1H, NH). ^13^C NMR (400 MHz, DMSO-d_6_, δ ppm): 31.80 (–CH_2_), Arom-C [124.09 (CH), 126.64 (CH), 129.23 (CH), 130.41 (CH), 133.57 (C), 135.51 (C), 139.82 (C), 150.33 (C)], Thiadiazole-C [159.88 (C), 160.81 (C)], 165.40 C=O. *Anal.* Calcd. for C_16_H_10_Cl_2_N_4_O_3_S: C, 46.96; H, 2.46; N, 13.69. Found: C, 46.88; H, 2.59; N, 13.562. MS: *m/z*: 408.93 (M^+^, 100), 410.96 (M + 1, 93).

#### *N*-(5-(2,6-Dichlorobenzyl)-1,3,4-thiadiazol-2-yl)-4-(methylthio)benzamide (**6c**)

White solid, yield: 1.13 g (69%), m.p. 282–283 °C (DMF-EtOH, 1:1). IR (ATR, cm^−1^): 3132 (–NH–), 3035 (Ar–CH), 2921 (Aliphatic CH), 1674 (C=O), 1595 (C=N). ^1^H NMR (400 MHz, DMSO-d_6_) δ (ppm): 2.48 (t, 3H, –CH_3_), 4.61 (s, 2H, –CH_2_), Arom-H [8.00 (d, *J* = 8.0 Hz, 2H), 7.54 (d, *J* = 8.4 Hz, 2H), 7.37 (m, 3H)], 12.90 (s, 1H, NH). ^13^C NMR (400 MHz, DMSO-d_6_, δ ppm): 14.95 (CH_3_), 31.79 (–CH_2_), Arom-C [125.32 (CH), 127.54 (CH), 129.22 (CH), 130.64 (CH), 133.66 (C), 135.52 (C), 139.63 (C), 145.70 (C)], Thiadiazole-C [160.46 (C), 163.66 (C)], 165.63 C=O. *Anal.* Calcd. for C_17_H_13_Cl_2_N_3_OS_2_: C, 49.76; H, 3.19; N, 10.24. Found: C, 49.68; H, 3.06; N, 10.12. MS (ESI–*m/z*): 411.38 (M + 1, 76), 413.34 (M + 2, 48).

#### *N*-(5-(2,6-Dichlorobenzyl)-1,3,4-thiadiazol-2-yl)-3,4-dichlorobenzamide (**6d**)

White solid, yield: 1.25 g (73%), m.p. 295–296 °C (DMF-EtOH, 1:15). IR (ATR, cm^−1^): 3142 (–NH–), 3084 (Ar–CH), 2967 (Aliphatic CH), 1682 (C=O), 1560 (C=N). ^1^H NMR (400 MHz, DMSO-d_6_) δ (ppm): 4.62 (s, 2H, –CH_2_), Arom-H [8.30 (s, 1H), 7.99 (d, *J* = 8.0 Hz, 1H), 7.80 (d, *J* = 8.4 Hz, 1H), 7.53 (d, *J* = 8.0 Hz, 2H), 7.38 (t, *J* = 8.0, 7.2 Hz, 1H)], 13.15 (s, 1H, NH). ^13^C NMR (400 MHz, DMSO-d_6_, δ ppm): 31.84 (–CH_2_), Arom-C [129.05 (CH), 129.22 (CH), 129.97 (CH), 130.68 (CH), 130.76 (CH), 131.42 (C), 131.98 (C), 133.54 (C), 135.51 (C), 136.16 (C)], Thiadiazole-C [159.85 (C), 160.96 (C)], 166.16 C=O. *Anal.* Calcd. for C_16_H_9_Cl_4_N_3_OS: C, 44.37; H, 2.09; N, 9.70. Found: C, 44.23; H, 2.01; N, 9.57. MS (ESI–*m/z*): 433.71 (M + 1, 100).

#### *N*-(5-(2,6-Dichlorobenzyl)-1,3,4-thiadiazol-2-yl)-3,4-difluorobenzamide (**6e**)

White solid, yield: 1.30 g (80%), m.p. 304–305 °C (DMF-EtOH, 1:15). IR (ATR, cm^−1^): 3142 (–NH–), 3087 (Ar–CH), 2941 (Aliphatic CH), 1663 (C=O), 1563 (C=N). ^1^H NMR (400 MHz, DMSO-d_6_) δ (ppm): 4.61 (s, 2H, –CH_2_), Arom-H [8.13 (t, 1H), 7.95 (s, 1H), 7.60 (q, 1H), 7.53 (d, *J* = 8.4 Hz, 2H), 7.38 (t, *J* = 8.0, 8.0 Hz, 1H)], 13.09 (s, 1H, NH). ^13^C NMR (400 MHz, DMSO-d_6_, δ ppm): 31.82 (–CH_2_), Arom-C [118.32 (CH), 126.65 (CH), 129.21 (CH), 130.65 (CH), 133.54 (CH), 135.51 (C), 148.33 (C), 150.91 (C), 151.47 (C), 154.11 (C)], Thiadiazole-C [159.80 (C), 161.90 (C)], 163.80 C=O. *Anal.* Calcd. for C_16_H_9_Cl_2_F_2_N_3_OS: C, 48.02; H, 2.27; N, 10.50. Found: C, 48.11; H, 2.12; N, 10.36. MS (ESI–*m/z*): 400.00 (M^+^, 94), 402.26 (M + 2, 54).

#### *N*-(5-(2,6-Dichlorobenzyl)-1,3,4-thiadiazol-2-yl)-3,4-dimethoxybenzamide (**6f**)

White solid, yield: 1.07 g (64%), m.p. 252–253 °C (DMF-EtOH, 1:1). IR (ATR, cm^−1^): 3155 (–NH–), 3047 (Ar–CH), 2941 (Aliphatic CH), 1661 (C=N), 1587 (C=N). ^1^H NMR (400 MHz, DMSO-d_6_) δ (ppm): 3.82 (s, 6H, –OCH_3_), 4.60 (s, 2H, –CH_2_), Arom-H [7.71 (s, 2H), 7.52 (s, 2H), 7.37 (s, 1H), 7.08 (s, 1H)], 12.82 (s, 1H, NH). ^13^C NMR (400 MHz, DMSO-d_6_, δ ppm): 31.76 (–CH_2_), 56.10 (–OCH_3_), 56.17 (–OCH_3_), Arom-C [111.59 (CH), 122.85 (CH), 123.66 (CH), 129.19 (CH), 130.59 (C), 133.68 (CH), 135.51 (C), 148.81 (C), 153.17 (C), 160.13 (C)], Thiadiazole-C [160.59 (C), 162.74 (C)], 164.73 C=O. *Anal.* Calcd. for C_18_H_15_Cl_2_N_3_O_3_S: C, 50.95; H, 3.56; N, 9.90. Found: C, 50.78; H, 3.46; N, 9.78. MS (ESI–*m/z*): 423.91 (M^+^, 100), 425.87 (M + 2, 66).

#### *N*-(5-(2,6-Dichlorobenzyl)-1,3,4-thiadiazol-2-yl)-3,5-dimethoxybenzamide (**6g**)

White solid, yield: 1.00 g (59%), m.p. 204–205 °C (DMF-EtOH, 1:1). IR (ATR, cm^−1^): 3138 (–NH–), 3032 (Ar–CH), 2959 (Aliphatic CH), 1683 (C=O), 1596 (C=N). ^1^H NMR (400 MHz, DMSO-d_6_) δ (ppm): 3.76 (s, 6H, –OCH_3_), 4.39 (s, 2H, –CH_2_), Arom-H [7.49 (d, *J* = 8.0 Hz, 2H), 7.34 (t, *J* = 8.0, 7.6 Hz, 1H), 7.04 (s, 2H), 6.71 (s, 1H)], 13.01 (bs, 1H, NH). ^13^C NMR (400 MHz, DMSO-d_6_, δ ppm): 31.76 (–CH_2_), 56.09 (–OCH_3_), 56.16 (–OCH_3_), Arom-C [107.89 (CH), 109.73 (CH), 129.17 (CH), 130.59 (CH), 133.64 (C), 135.53 (C), 146.41 (C), 160.48 (C)], Thiadiazole-C [153.18 (C), 162.75 (C)], 164.33 C=O. *Anal.* Calcd. for C_18_H_15_Cl_2_N_3_O_3_S: C, 50.95; H, 3.56; N, 9.90. Found: C, 50.79; H, 3.50; N, 9.81. MS (ESI–*m/z*): 423.91 (M^+^, 100).

#### *N*-(5-(2,6-Dichlorobenzyl)-1,3,4-thiadiazol-2-yl)-3,5-difluorobenzamide (**6h**)

White solid, yield: 1.18 g (74%), m.p. 270–271 °C (DMF-EtOH, 1:1). IR (ATR, cm^−1^): 3147 (–NH–), 3044 (Ar–CH), 2928 (Aliphatic CH), 1679 (C=O), 1595 (C=N). ^1^H NMR (400 MHz, DMSO-d_6_) δ (ppm): 4.62 (s, 2H, –CH_2_), Arom-H [7.76 (s, 2H), 7.54 (bd, 3H), 7.39 (t, 1H)], 13.16 (s, 1H, NH). ^13^C NMR (400 MHz, DMSO-d_6_, δ ppm): 31.84 (–CH_2_), Arom-C [108.80 (CH), 112.24 (CH), 112.43 (C), 129.22 (CH), 130.68 (CH), 133.51 (C), 135.51 (C), 163.80 (C)], Thiadiazole-C [161.35 (C), 161.48 (C)], 164.93 C=O. *Anal.* Calcd. for C_16_H_9_Cl_2_F_2_N_3_OS: C, 48.02; H, 2.27; N, 10.50. Found: C, 47.96; H, 2.20; N, 10.56. MS (ESI–*m/z*): 399.80 (M-1, 100), 401.83 (M + 1, 74).

#### *N*-(5-(2,6-Dichlorobenzyl)-1,3,4-thiadiazol-2-yl)-3,5-dichlorobenzamide (**6i**)

White solid, yield: 1.33 g (77%), m.p. 260–261 °C (DMF-EtOH, 1:1). IR (ATR, cm^−1^): 3147 (–NH–), 3070 (Ar–CH), 2969 (Aliphatic CH), 1663 (C=O), 1563 (C=N). ^1^H NMR (400 MHz, DMSO-d_6_) δ (ppm): 4.62 (s, 2H, –CH_2_), Arom-H [8.06 (s, 2H), 7.89 (s, 1H), 7.54 (d, *J* = 8.0 Hz, 2H),7.39 (t, *J* = 8.0, 7.6 Hz, 1H)], 13.16 (s, 1H, NH). ^13^C NMR (400 MHz, DMSO-d_6_, δ ppm): 31.87 (–CH_2_), Arom-C [127.60 (CH), 129.22 (CH), 130.69 (C), 132.50 (CH), 133.50 (C), 134.90 (C), 135.47 (C), 135.52 (C)], Thiadiazole-C [160.08 (C), 162.54 (C)], 163.93 C=O. *Anal.* Calcd. for C_16_H_9_Cl_4_N_3_OS: C, 44.37; H, 2.09; N, 9.70. Found: C, 44.22; H, 2.18; N, 9.52. MS (ESI–*m/z*): 431.79 (M-1, 76).

#### *N*-(5-(2,6-Dichlorobenzyl)-1,3,4-thiadiazol-2-yl)-4-ethylbenzamide (**6j**)

White solid, yield: 1.02 g (65%), m.p. 275–276 °C (DMF-EtOH, 1:1). IR (ATR, cm^−1^): 3184 (–NH–), 3059 (Ar–CH), 2964 (Aliphatic CH), 1662 (C=O), 1576 (C=N). ^1^H NMR (400 MHz, DMSO-d_6_) δ (ppm): 1.17 (t, 3H, –CH_3_), 2.65 (q, 2H, –CH_2_–), 4.61 (s, 2H, –CH_2_), Arom-H [7.99 (d, *J* = 8.0 Hz, 2H), 7.54 (d, *J* = 8.4 Hz, 2H), 7.36 (m, 3H)], 12.87 (s, 1H, NH). ^13^C NMR (400 MHz, DMSO-d_6_, δ ppm): 15.61 (–CH_3_), 28.58 (–CH_2_–), 31.79 (–CH_2_), Arom-C [128.47 (CH), 128.95 (CH), 129.01 (CH), 129.21 (CH), 130.62 (C), 133.66 (C), 135.52 (C), 149.83 (C)], Thiadiazole-C [159.65 (C), 160.72 (C)], 165.40 C=O. *Anal.* Calcd. for C_18_H_15_Cl_2_N_3_OS: C, 55.11; H, 3.85; N, 10.71. Found: C, 55.02; H, 3.79; N, 10.63. MS (ESI–*m/z*): 393.77 (M + 1, 93).

#### *N*-(5-(2,6-Dichlorobenzyl)-1,3,4-thiadiazol-2-yl)-4-(trifluoromethyl)benzamide (**6k**)

White solid, yield: 1.07 g (62%), m.p. 274–275 °C (DMF-EtOH, 1:1). IR (ATR, cm^−1^): 3152 (–NH–), 3041 (Ar–CH), 2943 (Aliphatic CH), 1680 (C=O), 1531 (C=N). ^1^H NMR (400 MHz, DMSO-d_6_) δ (ppm): 4.63 (s, 2H, –CH_2_), Arom-H [8.23 (d, *J* = 8.0 Hz, 2H), 7.90 (d, *J* = 8.4 Hz, 2H), 7.54 (d, *J* = 8.4 Hz, 2H), 7.39 (t, *J* = 7.6, 7.6 Hz, 1H)], 13.26 (s, 1H, NH). ^13^C NMR (400 MHz, DMSO-d_6_, δ ppm): 31.84 (–CH_2_), 122.84 (CF_3_), Arom-C [125.56 (CH), 125.99 (CH), 126.03 (CH), 129.22 (CH), 129.78 (C), 130.68 (C), 133.56 (C), 135.52 (C)], Thiadiazole-C [159.86 (C), 160.84 (C)], 165.53 C=O. *Anal.* Calcd. for C_17_H_10_Cl_2_F_3_N_3_OS: C, 47.24; H, 2.33; N, 9.72. Found: C, 47.36; H, 2.26; N, 9.65. MS (ESI–*m/z*): 431.86 (M-1, 82).

#### *N*-(5-(2,6-Dichlorobenzyl)-1,3,4-thiadiazol-2-yl)-4-cyanobenzamide (**6l**)

White solid, yield: 1.26 g (81%), m.p. 334–335 °C (DMF-EtOH, 1:1). IR (ATR, cm^−1^): 3142 (–NH–), 3094 (Ar–CH), 2921 (Aliphatic CH), 2235 (CN), 1684 (C=O), 1542 (C=N). ^1^H NMR (400 MHz, DMSO-d_6_) δ (ppm): 4.63 (s, 2H, –CH_2_), Arom-H [8.18 (d, *J* = 8.4 Hz, 2H), 8.01 (d, *J* = 8.0 Hz, 2H), 7.54 (d, *J* = 8.4 Hz, 2H), 7.38 (t, *J* = 8.0, 8.0 Hz, 1H)], 13.27 (s, 1H, NH). ^13^C NMR (400 MHz, DMSO-d_6_, δ ppm): 31.86 (–CH_2_), 118.56 (CN), Arom-C [115.40 (C), 125.54 (CH), 129.23 (CH), 129.61 (CH), 130.70 (CH), 133.03 (C), 133.53 (C), 135.52 (C)], Thiadiazole-C [159.88 (C), 160.86 (C)], 165.80 C=O. *Anal.* Calcd. for C_17_H_10_Cl_2_N_4_OS: C, 52.45; H, 2.59; N, 14.39. Found: C, 52.36; H, 2.46; N, 14.50. MS (ESI–*m/z*): 385.22 (M-4, 100).

#### *N*-(5-(2,6-Dichlorobenzyl)-1,3,4-thiadiazol-2-yl)acetamide (**6m**)

White solid, yield: 1.03 g (83%), m.p. 284–285 °C (DMF-EtOH, 1:15). IR (ATR, cm^−1^): 3158 (–NH–), 3052 (Ar–CH), 2976 (Aliphatic CH), 1698 (C=O), 1563 (C=N). ^1^H NMR (400 MHz, DMSO-d_6_) δ (ppm): 2.12 (s, 3H, –CH_3_), 4.56 (s, 2H, –CH_2_), Arom-H [7.59 (d, *J* = 7.6 Hz, 2H), 7.36 (t, *J* = 8.4, 8.0 Hz, 1H)], 13.26 (s, 1H, NH). ^13^C NMR (400 MHz, DMSO-d_6_, δ ppm): 22.77 (CH_3_), 31.75 (–CH_2_), Arom-C [129.15 (CH), 130.56 (CH), 133.63 (C), 135.48 (C)], Thiadiazole-C [159.99 (C), 160.26 (C)], 169.01 C=O. *Anal.* Calcd. for C_11_H_9_Cl_2_N_3_OS: C, 43.72; H, 3.00; N, 13.91. Found: C, 43.76; H, 3.09; N, 13.86. MS (ESI–*m/z*): 302.01 (M^+^, 85).

#### Ethyl 5-(2,6-Dichlorobenzyl)-1,3,4-thiadiazol-2-ylcarbamate (**6n**)

White solid, yield: 0.76 g (56%), m.p. 217–218 °C (DMF-EtOH, 1:5). IR (ATR, cm^−1^): 3160 (–NH–), 3022 (Ar–CH), 2982 (Aliphatic CH), 1720 (C=O), 1569 (C=N). ^1^H NMR (400 MHz, DMSO-d_6_) δ (ppm): 1.23 (t, 3H, –CH_3_), 4.17 (q, 2H, –OCH_2_–), 4.41 (s, 2H, –CH_2_), Arom-H [7.51 (d, 2H), 7.36 (t, 1H)], 12.08 (s, 1H, NH). ^13^C NMR (400 MHz, DMSO-d_6_, δ ppm): 14.65 (–CH_3_), 27.00 (–CH_2_–), 62.60 (–OCH_2_–), Arom-C [129.20 (CH), 130.37 (CH), 133.83 (C), 135.42 (C)], Thiadiazole-C [160.63 (C), 161.48 (C)], 162.49 C=O. *Anal.* Calcd. for C_12_H_11_Cl_2_N_3_O_2_S: C, 43.39; H, 3.34; N, 12.65. Found: C, 43.33; H, 3.27; N, 12.56. MS (ESI–*m/z*): 331.14 (M-1, 93).

#### *N*-(5-(2-Chloro-6-fluorobenzyl)-1,3,4-thiadiazol-2-yl)benzamide (**7a**)

White solid, yield:1.13 g (81%), m.p. 258–259 °C (DMF-EtOH, 1:2). IR (ATR, cm^−1^): 3174 (–NH–), 3054 (Ar–CH), 2977 (Aliphatic CH), 1670 (C=O), 1580 (C=N). ^1^H NMR (400 MHz, DMSO-d_6_) δ (ppm): 4.49 (s, 2H, –CH_2_), Arom-H [8.06 (d, *J* = 7.6 Hz, 2H), 7.62 (t, *J* = 6.8 Hz, 1H), 7.52 (t, *J* = 7.2 Hz, 2H), 7.41 (bs, 2H), 7.30 (bd, 1H)], 12.99 (s, 1H, NH). ^13^C NMR (400 MHz, DMSO-d_6_, δ ppm): 26.95 (–CH_2_), Arom-C [115.27 (CH), 123.79 (CH), 126.14 (C), 128.80 (CH), 129.07 (CH), 130.63 (CH), 131.92 (CH), 133.41 (C), 134.82 (C), 161.13 (C)], Thiadiazole-C [160.08 (C), 162.54 (C)], 165.55 C=O. *Anal.* Calcd. for C_16_H_11_ClFN_3_OS: C, 55.25; H, 3.19; N, 12.08. Found: C, 55.31; H, 3.21; N, 12.11. MS: *m/z*: 347.79 (M^+^, 100), 348.81 (M + 1, 32).

#### *N*-(5-(2-Chloro-6-fluorobenzyl)-1,3,4-thiadiazol-2-yl)-4-nitrobenzamide (**7b**)

White solid, yield:1.19 g (76%), m.p. 257–258 °C (DMF-EtOH, 1:3). IR (ATR, cm^−1^): 3116 (–NH–), 3039 (Ar–CH), 2924 (Aliphatic CH), 1679 (C=O), 1604 (C=N). ^1^H NMR (400 MHz, DMSO-d_6_) δ (ppm): 4.50 (s, 2H, –CH_2_), Arom-H [8.34 (d, *J* = 7.6 Hz, 2H), 8.26 (d, *J* = 8.0 Hz, 2H),7.38 (d, 2H), 7.31 (bd, 1H)], 13.39 (s, 1H, NH). ^13^C NMR (400 MHz, DMSO-d_6_, δ ppm): 27.03 (–CH_2_), Arom-C [115.28 (CH), 124.09 (CH), 126.12 (CH), 126.16 (C), 130.41 (CH), 130.70 (CH), 130.89 (C), 134.82 (C), 147.08 (C), 160.08 (C)], Thiadiazole-C [150.26 (C), 162.54 (C)], 167.10 C=O. *Anal.* Calcd. for C_16_H_10_ClFN_4_O_3_S: C, 48.92; H, 2.57; N, 14.26. Found: C, 48.88; H, 2.59; N, 14.22. MS: *m/z*: 392.90 (M^+^, 100), 394.72 (M + 2, 54).

#### *N*-(5-(2-Chloro-6-fluorobenzyl)-1,3,4-thiadiazol-2-yl)-4-(methylthio) benzamide (**7c**)

White solid, yield:1.21 g (77%), m.p. 275–276 °C (DMF-EtOH, 1:2). IR (ATR, cm^−1^): 3158 (–NH–), 3043 (Ar–CH), 2949 (Aliphatic CH), 1658 (C=O), 1591 (C=N). ^1^H NMR (400 MHz, DMSO-d_6_) δ (ppm): 2.51 (s, 3H, –CH_3_), 4.48 (s, 2H, –CH_2_), Arom-H [8.01 (d, *J* = 6.8 Hz, 2H), 7.37 (d, *J* = 6.8 Hz, 2H), 7.35 (s, 2H), 7.29 (bs, 1H)], 12.90 (s, 1H, NH). ^13^C NMR (400 MHz, DMSO-d_6_, δ ppm): 14.39 (CH_3_), 26.95 (–CH_2_), Arom-C [115.20 (CH), 123.89 (CH), 125.32 (C), 126.12 (CH), 130.63 (CH), 130.73 (CH), 134.88 (C), 139.69 (C), 145.70 (C), 161.14 (C)], Thiadiazole-C [160.09 (C), 162.54 (C)], 165.48 C=O. *Anal.* Calcd. for C_17_H_13_ClFN_3_OS_2_: C, 51.84; H, 3.33; N, 10.67. Found: C, 51.79; H, 3.36; N, 10.62. MS (ESI–*m/z*): 411.38 (M + 1, 96), 413.34 (M + 2, 48).

#### *N*-(5-(2-Chloro-6-fluorobenzyl)-1,3,4-thiadiazol-2-yl)-3,4-dichloro benzamide (**7d**)

White solid, yield:1.27 g (76%), m.p. 214–215 °C (DMF-EtOH, 1:11). IR (ATR, cm^−1^): 3092 (–NH–), 3010 (Ar–CH), 2928 (Aliphatic CH), 1674 (C=O), 1591 (C=N). ^1^H NMR (400 MHz, DMSO-d_6_) δ (ppm): 4.82 (s, 2H, –CH_2_), Arom-H [8.30 (s, 1H), 7.99 (d, *J* = 8.4 Hz, 1H), 7.79 (d, *J* = 8.4 Hz, 1H), 7.38 (d, 2H), 7.29 (t, 1H)], 13.15 (s, 1H, NH). ^13^C NMR (400 MHz, DMSO-d_6_, δ ppm): 27.00 (–CH_2_), Arom-C [115.26 (CH), 123.66 (CH), 126.11 (C), 126.14 (CH), 129.03 (CH), 130.66 (CH), 130.76 (CH), 131.39 (C), 131.99 (C), 134.82 (C), 136.17 (C), 160.08 (C)], Thiadiazole-C [159.85 (C), 162.53 (C)], 164.12 C=O. *Anal.* Calcd. for C_16_H_9_Cl_3_FN_3_OS: C, 46.12; H, 2.18; N, 10.08. Found: C, 46.17; H, 2.15; N, 10.12. MS (ESI–*m/z*): 417.60 (M + 1, 100).

#### *N*-(5-(2-Chloro-6-fluorobenzyl)-1,3,4-thiadiazol-2-yl)-3,4-difluorobenz- amide (**7e**)

White solid, yield:1.21 g (79%), m.p. 278–279 °C (DMF-EtOH, 1:10). IR (ATR, cm^−1^): 3157 (–NH–), 3063 (Ar–CH), 2979 (Aliphatic CH), 1667 (CvO), 1608 (C=N). ^1^H NMR (400 MHz, DMSO-d_6_) δ (ppm): 4.49 (s, 2H, –CH_2_), Arom-H [8.14 (t, 1H), 7.95 (bs, 1H), 7.62 (q, 1H), 7.39 (bs, 2H), 7.29 (t, 1H)], 13.09 (s, 1H, NH). ^13^C NMR (400 MHz, DMSO-d_6_, δ ppm): 26.99 (–CH_2_), Arom-C [115.26 (CH), 118.33 (CH), 123.69 (CH), 126.11 (CH), 126.71 (CH), 130.66 (C), 134.82 (CH), 148.45 (C), 150.78 (C), 151.59 (C), 154.12 (C), 160.08 (C)], Thiadiazole-C [157.63 (C), 161.78 (C)], 162.55 C=O. *Anal.* Calcd. for C_16_H_9_ClF_3_N_3_OS: C, 50.07; H, 2.36; N, 10.95. Found: C, 50.11; H, 2.33; N, 10.97. MS (ESI–*m/z*): 383.78 (M^+^, 70), 385.15 (M + 2, 100).

#### *N*-(5-(2-Chloro-6-fluorobenzyl)-1,3,4-thiadiazol-2-yl)-3,4-dimethoxy benzamide (**7f**)

White solid, yield:1.11 g (68%), m.p. 248–249 °C (DMF-EtOH, 1:4). IR (ATR, cm^−1^): 3178 (–NH–), 3088 (Ar–CH), 2940 (Aliphatic CH), 1661 (C=N), 1588 (C=N). ^1^H NMR (400 MHz, DMSO-d_6_) δ (ppm): 3.82 (s, 6H, –OCH_3_), 4.47 (s, 2H, –CH_2_), Arom-H [7.73 (s, 2H), 7.39 (s, 2H), 7.30 (d, 1H), 7.08 (d, 1H)], 12.82 (s, 1H, NH). ^13^C NMR (400 MHz, DMSO-d_6_, δ ppm): 26.89 (–CH_2_), 56.10 (–OCH_3_), 56.17 (–OCH_3_), Arom-C [111.58 (CH), 115.03 (CH), 115.25 (CH), 122.86 (CH), 123.65 (CH), 123.83 (C), 126.14 (C), 130.70 (CH), 134.82 (C), 148.82 (C), 153.17 (C), 160.08 (C)], Thiadiazole-C [160.97 (C), 162.54 (C)], 164.70 C=O. *Anal.* Calcd. for C_18_H_15_ClFN_3_O_3_S: C, 53.01; H, 3.71; N, 10.30. Found: C, 52.97; H, 3.74; N, 10.28. MS (ESI–*m/z*): 407.81 (M^+^, 100), 409.91 (M + 2, 37).

#### *N*-(5-(2-Chloro-6-fluorobenzyl)-1,3,4-thiadiazol-2-yl)-3,5-dimethoxy benzamide (**7g**)

White solid, yield: 0.93 g (57%), m.p. 252–254 °C (DMF-EtOH, 1:1). IR (ATR, cm^−1^): 3268 (–NH–), 3109 (Ar–CH), 2947 (Aliphatic CH), 1624 (C=O), 1580 (C=N). ^1^H NMR (400 MHz, DMSO-d_6_) δ (ppm): 3.76 (s, 6H, –OCH_3_), 4.39 (s, 2H, –CH_2_), Arom-H [7.75 (d, 1H), 7.34 (t, 1H), 7.04 (s, 2H), 6.71 (s, 2H)], 13.03 (bs, 1H, NH). ^13^C NMR (400 MHz, DMSO-d_6_, δ ppm): 26.93 (–CH_2_), 56.10 (–OCH_3_), 56.17 (–OCH_3_), Arom-C [111.56 (CH), 115.31 (CH), 122.86 (CH), 123.65 (CH), 126.14 (C), 130.76 (CH), 134.87 (C), 148.82 (C), 160.08 (C), 160.97 (C)], Thiadiazole-C [153.25 (C), 162.54 (C)], 164.70 C=O. *Anal.* Calcd. for C_18_H_15_ClFN_3_O_3_S: C, 53.01; H, 3.71; N, 10.30. Found: C, 53.05; H, 3.69; N, 10.29. MS (ESI–*m/z*): 409.09 (M + 1, 96).

#### *N*-(5-(2-Chloro-6-fluorobenzyl)-1,3,4-thiadiazol-2-yl)-3,5-difluoro benzamide (**7h**)

White solid, yield: 1.24 g (81%), m.p. 261–262 °C (DMF-EtOH, 1:2). IR (ATR, cm^−1^): 3154 (–NH–), 3096 (Ar–CH), 2932 (Aliphatic CH), 1677 (C=O), 1597 (C=N). ^1^H NMR (400 MHz, DMSO-d_6_) δ (ppm): 4.49 (s, 2H, –CH_2_), Arom-H [7.77 (s, 2H), 7.58 (t, 1H), 7.40 (bs, 2H), 7.30 (t, 1H)], 13.16 (s, 1H, NH). ^13^C NMR (400 MHz, DMSO-d_6_, δ ppm): 26.99 (–CH_2_), Arom-C [108.64 (CH), 112.16 (CH), 112.44 (CH), 115.28 (CH), 123.68 (C), 126.13 (CH), 130.69 (C), 134.82 (C), 160.08 (C), 163.81 (C)], Thiadiazole-C [161.35 (C), 162.54 (C)], 163.94 C=O. *Anal.* Calcd. for C_16_H_9_ClF_3_N_3_OS: C, 50.07; H, 2.36; N, 10.95. Found: C, 50.05; H, 2.38; N, 10.99. MS (ESI–*m/z*): 383.87 (M^+^, 100), 385.83 (M + 2, 53).

#### *N*-(5-(2-Chloro-6-fluorobenzyl)-1,3,4-thiadiazol-2-yl)-3,5-dichloro benzamide (**7i**)

White solid, yield:1.25 g (75%), m.p. 277–278 °C (DMF-EtOH, 1:2). IR (ATR, cm^−1^): 3140 (–NH–), 3082 (Ar–CH), 2913 (Aliphatic CH), 1677 (C=O), 1569 (C=N). ^1^H NMR (400 MHz, DMSO-d_6_) δ (ppm): 4.62 (s, 2H, –CH_2_), Arom-H [8.06 (s, 2H), 7.89 (s, 1H), 7.40 (m, 2H),7.29 (m, 1H)], 13.17 (s, 1H, NH). ^13^C NMR (400 MHz, DMSO-d_6_, δ ppm): 27.00 (–CH_2_), Arom-C [115.28 (CH), 123.64 (CH), 126.12 (C), 127.60 (CH), 130.68 (CH), 130.78 (CH), 132.51 (C), 134.81 (C), 134.87 (C), 134.91 (C)], Thiadiazole-C [160.07 (C), 162.54 (C)], 164.21 C=O. *Anal.* Calcd. for C_16_H_9_Cl_3_FN_3_OS: C, 46.12; H, 2.18; N, 10.08. Found: C, 46.09; H, 2.16; N, 10.11. MS (ESI–*m/z*): 417.10 (M^+^, 90).

#### *N*-(5-(2-Chloro-6-fluorobenzyl)-1,3,4-thiadiazol-2-yl)-4-ethylbenzamide (**7j**)

White solid, yield: 1.11 g (74%), m.p. 243–244 °C (DMF-EtOH, 1:1). IR (ATR, cm^−1^): 3182 (–NH–), 3056 (Ar–CH), 2966 (Aliphatic CH), 1662 (C=O), 1580 (C=N). ^1^H NMR (400 MHz, DMSO-d_6_) δ (ppm): 1.20 (t, 3H, –CH_3_), 2.66 (q, 2H, –CH_2_–), 4.48 (s, 2H, –CH_2_), Arom-H [7.99 (d, *J* = 8.0 Hz, 2H), 7.39 (m, 4H), 7.29 (m,1H)], 12.89 (s, 1H, NH). ^13^C NMR (400 MHz, DMSO-d_6_, δ ppm): 15.60 (–CH_3_), 26.92 (–CH_2_–), 28.57 (–CH_2_CH_3_), Arom-C [115.28 (CH), 123.63 (CH), 126.11 (C), 128.47 (CH), 128.95 (CH), 129.34 (CH), 130.62 (C), 134.87 (C), 149.84 (C), 160.09 (C)], Thiadiazole-C [161.07 (C), 162.55 (C)], 165.56 C=O. *Anal.* Calcd. for C_18_H_15_ClFN_3_OS: C, 57.52; H, 4.02; N, 11.18. Found: C, 57.49; H, 4.05; N, 11.21. MS (ESI–*m/z*): 375.02 (M-1, 91).

#### *N*-(5-(2-Chloro-6-fluorobenzyl)-1,3,4-thiadiazol-2-yl)-4-(trifluoromethyl)-benzamide (**7k**)

White solid, yield: 1.18 g (71%), m.p. 273–274 °C (DMF-EtOH, 1:1). IR (ATR, cm^−1^): 3144 (–NH–), 3052 (Ar–CH), 2933 (Aliphatic CH), 1677 (C=O), 1581 (C=N). ^1^H NMR (400 MHz, DMSO-d_6_) δ (ppm): 4.49 (s, 2H, –CH_2_), Arom-H [8.23 (d, *J* = 8.0 Hz, 2H), 7.90 (d, *J* = 8.4 Hz, 2H), 7.39 (m, 2H), 7.29 (m, 1H)], 13.27 (s, 1H, NH). ^13^C NMR (400 MHz, DMSO-d_6_, δ ppm): 26.97 (–CH_2_), 122.84 (CF_3_), Arom-C [115.27 (CH), 123.51 (CH), 125.98 (C), 126.15 (CH), 129.77 (CH), 130.66 (CH), 132.66 (C), 132.98 (C), 134.82 (C), 160.09 (C)], Thiadiazole-C [161.12 (C), 162.55 (C)], 164.96 C=O. *Anal.* Calcd. for C_17_H_10_ClF_4_N_3_OS: C, 49.11; H, 2.42; N, 10.11. Found: C, 49.07; H, 2.45; N, 10.13. MS (ESI–*m/z*): 415.90 (M^+^, 93).

#### *N*-(5-(2-Chloro-6-fluorobenzyl)-1,3,4-thiadiazol-2-yl)-4-cyanobenzamide (**7l**)

White solid, yield: 1.25 g (84%), m.p. 327–328 °C (DMF-EtOH, 1:2). IR (ATR, cm^−1^): 3149 (–NH–), 3071 (Ar–CH), 2926 (Aliphatic CH), 2243 (CN), 1679 (C=O), 1581 (C=N). ^1^H NMR (400 MHz, DMSO-d_6_) δ (ppm): 4.50 (s, 2H, –CH_2_), Arom-H [8.19 (d, *J* = 8.4 Hz, 2H), 8.01 (d, *J* = 8.4 Hz, 2H), 7.40 (m, 2H), 7.30 (m, 1H)], 13.25 (s, 1H, NH). ^13^C NMR (400 MHz, DMSO-d_6_, δ ppm): 27.02 (–CH_2_), 118.56 (CN), Arom-C [115.07 (CH), 115.30 (C), 126.14 (CH), 126.17 (CH), 129.62 (CH), 130.69 (CH), 130.79 (CH), 133.04 (C), 134.82 (C), 160.08 (C)], Thiadiazole-C [154.73 (C), 162.54 (C)], 165.79 C=O. *Anal.* Calcd. for C_17_H_10_ClFN_4_OS: C, 54.77; H, 2.70; N, 15.03. Found: C, 54.78; H, 2.68; N, 15.07. MS (ESI–*m/z*): 372.97 (M^+^, 92).

#### *N*-(5-(2-Chloro-6-fluorobenzyl)-1,3,4-thiadiazol-2-yl)acetamide (**7m**)

White solid, yield: 0.99 g (87%), m.p. 262–263 °C (DMF-EtOH, 1:8). IR (ATR, cm^−1^): 3158 (–NH–), 3038 (Ar–CH), 2908 (Aliphatic CH), 1699 (C=O), 1558 (C=N). ^1^H NMR (400 MHz, DMSO-d_6_) δ (ppm): 2.13 (s, 3H, –CH_3_), 4.44 (s, 2H, –CH_2_), Arom-H [7.38 (m, 2H), 7.28 (m, 1H)], 12.45 (s, 1H, NH). ^13^C NMR (400 MHz, DMSO-d_6_, δ ppm): 22.78 (CH_3_), 26.88 (–CH_2_), Arom-C [115.23 (CH), 123.79 (CH), 126.11 (C), 130.69 (CH), 134.84 (C), 160.07 (C)], Thiadiazole-C [159.08 (C), 162.51 (C)], 169.03 C=O. *Anal.* Calcd. for C_11_H_9_ClFN_3_OS: C, 46.24; H, 3.17; N, 14.71. Found: C, 46.26; H, 3.14; N, 14.69. MS (ESI–*m/z*): 286.11 (M + 1, 95).

#### Ethyl 5-(2-Chloro-6-fluorobenzyl)-1,3,4-thiadiazol-2-ylcarbamate (**7n**)

White solid, yield: 0.80 g (63%), m.p. 192–193 °C (DMF-EtOH, 1:5). IR (ATR, cm^−1^): 3160 (–NH–), 3037 (Ar–CH), 2982 (Aliphatic CH), 1720 (C=O), 1569 (C=N). ^1^H NMR (400 MHz, DMSO-d_6_) δ (ppm): 1.22 (t, 3H, –CH_3_), 4.16 (q, 2H, –OCH_2_–), 4.41 (s, 2H, –CH_2_), Arom-H [7.36 (m, 2H), 7.25 (m, 1H)], 12.09 (s, 1H, NH). ^13^C NMR (400 MHz, DMSO-d_6_, δ ppm): 14.65 (–CH_3_), 26.96 (–CH_2_–), 62.61 (–OCH_2_–), Arom-C [115.18 (CH), 123.53 (CH), 126.06 (C), 130.55 (CH), 134.77 (C), 160.03 (C)], Thiadiazole-C [154.34 (C), 161.47 (C)], 162.49 CvO. *Anal.* Calcd. for C_12_H_11_ClFN_3_O_2_S: C, 45.65; H, 3.51; N, 13.31. Found: C, 45.56; H, 3.48; N, 13.20. MS (ESI–*m/z*): 315.16 (M^+^, 96).

### Crystallographic analysis

The X-ray fraction data of the compound **7n** examined in this study was collected using the MoK_α_ ray at 293(2)K degree using a ‘Bruker APEX-II CCD diffractometer. The structures of the crystals were solved using direct methods in ShelXT [43] software. During the process, in order to determine the positions of the atoms, except for hydrogen, the refinement procedure was conducted using the ShelXL [44] software [45] that used the full-matrix least-squares method. After the structure solution and refinement procedures were finished, olex2 and MERCURY software were used in molecular drawings and calculations.

### Biological activity studies

#### Fungi culture

*Monilia fructigena, Fusarium oxysporum f*. sp*. lycopersici* and *Alternaria solani* plant pathogens were used for the tests. The pathogens were grown on a PDA (potato dextrose agar) medium at 22 ± 2 °C for about 7 days.

##### In vitro antifungal activity

Antifungal activity studies were determined using disk diffusion method [46]. The compounds were dissolved in dimethyl sulphoxide (DMSO). In the laminar flow cabin, Whatman no. 1 sterile filter paper discs (6 mm) were impregnated with 50 μl of the compounds (corresponding to 500 and 1000 μg/ml of compounds) and allowed to dry at room temperature (for 4 h) [47]. Then the compound impregnated paper discs were placed in a PDA medium in 90 mm sterile petri plates. Mycelium discs (5 mm diameter) of 7-day-old culture of test fungi were inoculated to the centre of the Petri plate. All fungi were incubated at 22 ± 2 °C. There is a 25 mm distance between the mycelium discs and paper discs. The obtained inhibition zones were recorded. As negative control, only DMSO was impregnated to discs. In the positive control, 80% thiram (3000 μg/ml) was used against the test fungi at the recommended dose. All antifungal activity values were determined by measuring inhibition zone distance between pathogen and paper disc [24].

Percent inhibition was calculated according to the following formula:

$${\text{\% Inhibition}} = {\text{Inhibition zone in treatment}}/{\text{Control*}} \times 1 0 0$$

*Control: Inhibition zone of positive control.

#### Lethal doses (LD_50_), minimum fungicidal concentration (MFC) and minimum inhibitory concentration (MIC)

Minimum inhibitory concentration (MIC) and Minimum fungicidal concentration (MFC) of the compounds were tested by the twofold serial dilution method. The test compound was dissolved in DMSO to obtain 1000 µg/ml stock solutions. For the MIC and MFC assay, each compound was prepared in the concentrations of 1000, 500, 250, 125, 62.5 and 31.25 µg/ml. Fifty microliter of concentrations of the compound was transferred on to paper disc. Then the same methods as described in the in vitro antifungal activity studies were applied. The MIC was defined as the lowest test concentration that allowed no detectable mycelium growth. The MFC was defined as the lowest test concentration that allows no mycelium growth of the organism on agar [48]. In addition, LD_50_ values were calculated. Six different doses used in the calculation of the MIC were calculated using the results of the inhibition zones. Lethal doses (LD) estimates for LD_50_ were determined with Polo Plus (LeOra software).

## Computational methods

JSME [49] molecular editor was used to generate the structures of the ligands and Open Babel v2.4.1 [50] was used for conversion of file formats and optimization of chemical structures.

2D Similarity search was conducted by Tanimoto similarity equation to extract compounds similar to our structures using a similarity threshold of 90% against NCBI׳s PubChem database consisting of 96,470,035 compounds [51–53].

The amino acid sequences were retrieved from the Universal Protein Resource (UniProt). NCBI BLAST server was used to find similar protein sequences that corresponds to our target organisms [31–33].

Homology modelling studies were performed through the Automated Comparative Protein Modelling Server (SWISS-MODEL) [34]. Structural validation of the model was done via RAMPAGE and ERRAT servers [35, 36].

Molecular dynamics simulation study was performed using GROMACS v5.1.4 [54–56]. The protonation states of residues were assigned at pH 7 in the PDB2PQR web server using PROPKA [57]. All the systems were solvated using SPC water model in a rectangular box. CHARMM36 force field was used to generate protein topology, whereas CHARMM General Force Field (CGenFF) [58, 59] was used in ligand parametrization. Na^+^ or Cl^−^ ions were used to neutralize the system. In order to remove the steric clashes in the system, steepest energy minimization was performed [60]. Long range electrostatic interactions were calculated by PME method [61]. The time step was kept at 2 fs during the simulation. Then the equilibration simulation of 1 ns was carried out under NVT and NPT conditions. Finally, a molecular dynamic simulation of 50 ns for each system was performed. All the graphs were generated using Xmgrace. LeDock was used as a molecular docking tool. LePro was used for the preparation of the receptor structures. All the heteroatoms were deleted from the structures and the hydrogens were added by LePro [62, 63].

Schrodinger’s QikProp [64] module was used to calculate some molecular descriptors such as molecular weight, total solvent accessible surface area (SASA), logarithm of octanol–water partition coefficient (QPlogPo/w), brain/blood partition coefficient (QPlogBB), apparent MDCK cell permeability (QPPMDCK), percent human oral absorption, polar surface area (PSA) and violations to the Lipinski’s rule of five [41].

## Additional file

**Additional file 1.** Experimental details and NMR spectra. **Tables S1–S3.** X-ray data of compound **7n**. **Figure S1.** The crystal structure of compound **7n**. **Figure S2.** Packaging of the compound **7n** over b-axis.** Figure S3.** The Radius of gyration (Rg) during 50 ns of MD simulation of STE/STE20/YSK protein kinase homology model. **Figures S4–S63.** ^1^H and ^13^C NMR spectra of all the compounds.
