# Supplementary material for: An integrated approach towards the development of novel antifungal agents containing thiadiazole: synthesis and a combined similarity search, homology modelling, molecular dynamics and molecular docking study
Source: Chem Cent J. 2018 Nov 23;12:121. doi: 10.1186/s13065-018-0485-3 (PMC6768040; doi:10.1186/s13065-018-0485-3)
Supplement: Supplementary file 1 — Additional file 1. Experimental details and NMR spectra. Tables S1–S3. X-ray data of compound 7n. Figure S1. The crystal structure of compound 7n. Figure S2. Packaging of the compound 7n over b-axis. Figure S3. The Radius of gyration (Rg) during 50 ns of MD simulation of STE/STE20/YSK protein kinase homology model. Figures S4–S63. 1H and 13C NMR spectra of all the compounds. [file 13065_2018_485_MOESM1_ESM.docx]

**Additional Material**

**for**

**An Integrated Approach Towards the Development of Novel Antifungal Agents Containing Thiadiazole: Synthesis and a Combined Similarity Search, Homology Modeling, Molecular Dynamics and Molecular Docking Study**

**Mustafa Er^1^*,** **Abdulati Miftah Abounakhla^1^, Hakan Tahtaci^1^, Ali Hasin Bawah^1^,**

**Süleyman Selim Çınaroğlu^2^, Abdurrahman Onaran^3^, Abdulilah Ece^4^***

^1^Department of Chemistry, Faculty of Science, Karabuk University, 78050 Karabuk, Turkey.

^2^Department of Medical Biotechnology, Institute of Health Sciences, Acıbadem Mehmet Ali Aydınlar University, İstanbul 34752, Turkey.

^3^Department of Plant Protection, Faculty of Agriculture, Gaziosmanpasa University 60250, Tokat, Turkey.

^4^Department of Pharmaceutical Chemistry, Faculty of Pharmacy, Biruni University, 34010, Istanbul, Turkey.

**Table of Contents**

All X-Ray Data of Compound **7n……………………………………………………...........S2-S5**

^1^H NMR and ^13^C NMR Spectra Copies of All Compounds**………………………………...S6-S36**


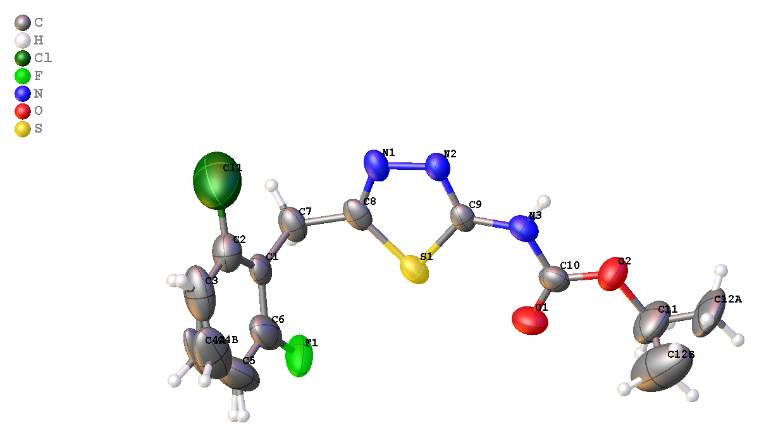


**Figure S1.** The crystal structure of compound **7n**.

**Table S1.** Data collection and refinement values of the compound **7n**.

| **Chemical Formula** | **C_12_H_11_ClFN_3_O_2_S** |
| --- | --- |
| Molecular Weight | 314.74 |
| Temperature/K | 293(2) |
| Crystal System | monoclinic |
| Space Group | C2/c |
| a/Å | 27.493(3) |
| b/Å | 4.9214(4) |
| c/Å | 23.539(3) |
| α/° | 90 |
| β/° | 115.002(4) |
| γ/° | 90 |
| Unit Cell Volume /Å^3^ | 2886.6(5) |
| Z (Number of Molecules in the Unit Cell) | 8 |
| ρ_calc_ (Calculated Density)g/cm^3^ | 1.448 |
| μ(Linear Absorption Coefficient) /mm^‑1^ | 0.424 |
| F(000) | 1288.0 |
| Crystal Dimensions/mm^3^ | 0.2 × 0.15 × 0.06 |
| Radiation | MoKα (λ = 0.71073) |
| 2Θ Data collection interval /° | 5.986 to 49.992 |
| Index range | -32 ≤ h ≤ 32, -5 ≤ k ≤ 5, -27 ≤ l ≤ 27 |
| Collected Reflection | 18341 |
| Independent reflection | 2467 [R_int_ = 0.0891, R_sigma_ = 0.0649] |
| Reflection/Parameter | 2467/42/202 |
| S (Goodness-of-fit) | 1.251 |
| Final R value [I>=2σ (I)] | R_1_ = 0.1385, wR_2_ = 0.2935 |
| Final R value [Total data] | R_1_ = 0.1732, wR_2_ = 0.3061 |
| ∆σmax, ∆σmin / e Å^-3^ | 0.79/-0.60 |

**Table S2.** Hydrogen bond geometry of the compound **7n** (Å, °)

| **D-H···A** | **D-H** | **H···A** | **D···A** | **D-H···A** |
| --- | --- | --- | --- | --- |
| N3- H3**···**N2^i^ | 0.86 | 2.04 | 2.89(10) | 167 |

Symmetry Code: i = 3/2-x,-1/2-y,1-z

**Table S3.** Bond lengths and bond angles (°) for compound **7n**.

| **Atom** | **Atom** | **Length (Å)** | **Atom** | **Atom** | **Length (Å)** |
| --- | --- | --- | --- | --- | --- |
| S1 | C9 | 1.727(8) | C1 | C7 | 1.499(11) |
| S1 | C8 | 1.741(9) | C1 | C2 | 1.352(13) |
| Cl1 | C2 | 1.622(12) | C1 | C6 | 1.398(13) |
| F1 | C6 | 1.494(14) | C8 | C7 | 1.488(11) |
| O2 | C10 | 1.316(10) | C2 | C3 | 1.387(16) |
| O2 | C11 | 1.459(12) | C6 | C5 | 1.384(18) |
| N3 | C9 | 1.361(10) | C3 | C4B | 1.34(3) |
| N3 | C10 | 1.362(11) | C3 | C4A | 1.35(3) |
| O1 | C10 | 1.206(10) | C5 | C4B | 1.36(3) |
| N2 | N1 | 1.374(9) | C5 | C4A | 1.36(3) |
| N2 | C9 | 1.289(10) | C11 | C12A | 1.37(2) |
| N1 | C8 | 1.281(10) | C11 | C12B | 1.38(2) |

| **Atom** | **Atom** | **Atom** | **Angle/˚** | **Atom** | **Atom** | **Atom** | **Angle/˚** |
| --- | --- | --- | --- | --- | --- | --- | --- |
| C9 | S1 | C8 | 86.1(4) | C7 | C8 | S1 | 123.0(7) |
| C10 | O2 | C11 | 115.9(8) | C8 | C7 | C1 | 112.6(7) |
| C9 | N3 | C10 | 124.8(7) | C1 | C2 | Cl1 | 120.3(8) |
| C9 | N2 | N1 | 112.3(7) | C1 | C2 | C3 | 124.1(12) |
| C8 | N1 | N2 | 113.2(7) | C3 | C2 | Cl1 | 115.6(11) |
| N3 | C9 | S1 | 123.6(6) | C1 | C6 | F1 | 121.0(9) |
| N2 | C9 | S1 | 114.5(6) | C5 | C6 | F1 | 118.8(11) |
| N2 | C9 | N3 | 121.9(7) | C5 | C6 | C1 | 120.1(13) |
| O2 | C10 | N3 | 110.0(8) | C4B | C3 | C2 | 116.2(15) |
| O1 | C10 | O2 | 126.8(8) | C4A | C3 | C2 | 116.9(16) |
| O1 | C10 | N3 | 123.2(8) | C4B | C5 | C6 | 118.5(16) |
| C2 | C1 | C7 | 123.1(9) | C4A | C5 | C6 | 119.7(16) |
| C2 | C1 | C6 | 116.6(9) | C3 | C4B | C5 | 122(3) |
| C6 | C1 | C7 | 120.2(9) | C12A | C11 | O2 | 106.1(13) |
| N1 | C8 | S1 | 113.8(6) | C12B | C11 | O2 | 111(2) |


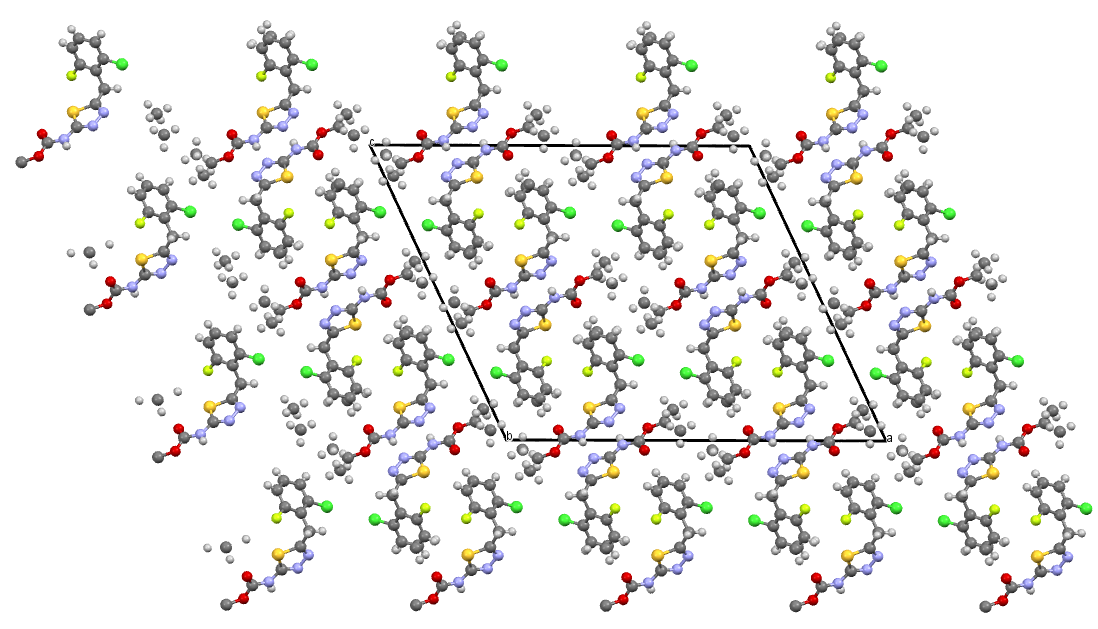


**Figure S2.** Packaging of the compound **7n** over b-axis.





**Figure S3.** The Radius of gyration (Rg) during 50 ns of MD simulation of STE/STE20/YSK protein kinase homology model.


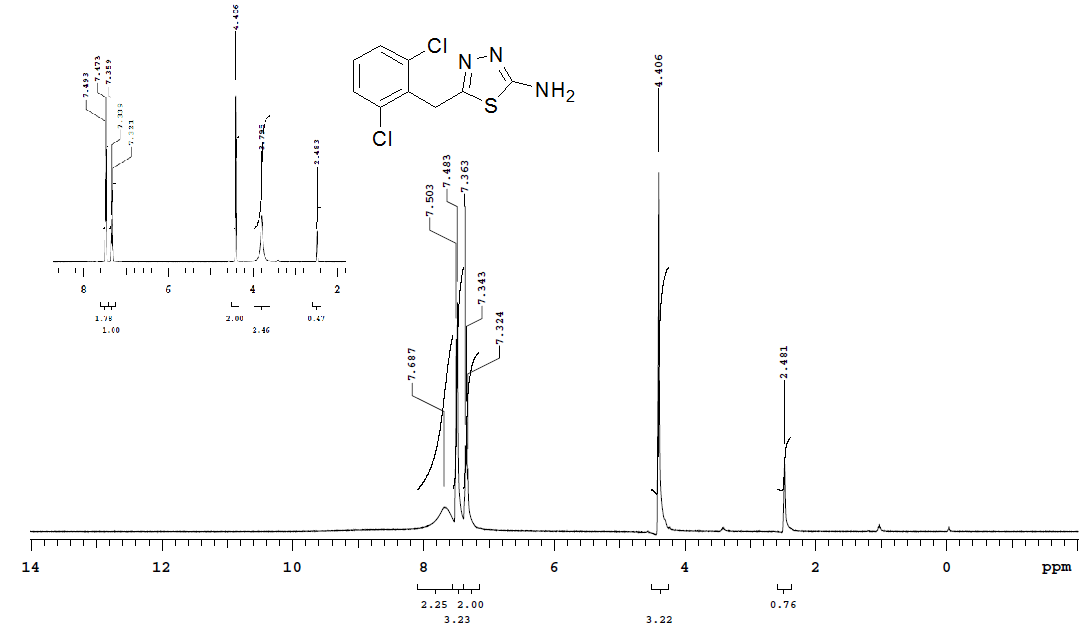


**Figure S4**. ^1^H NMR spectrum of compound **3** (DMSO-d_6_).


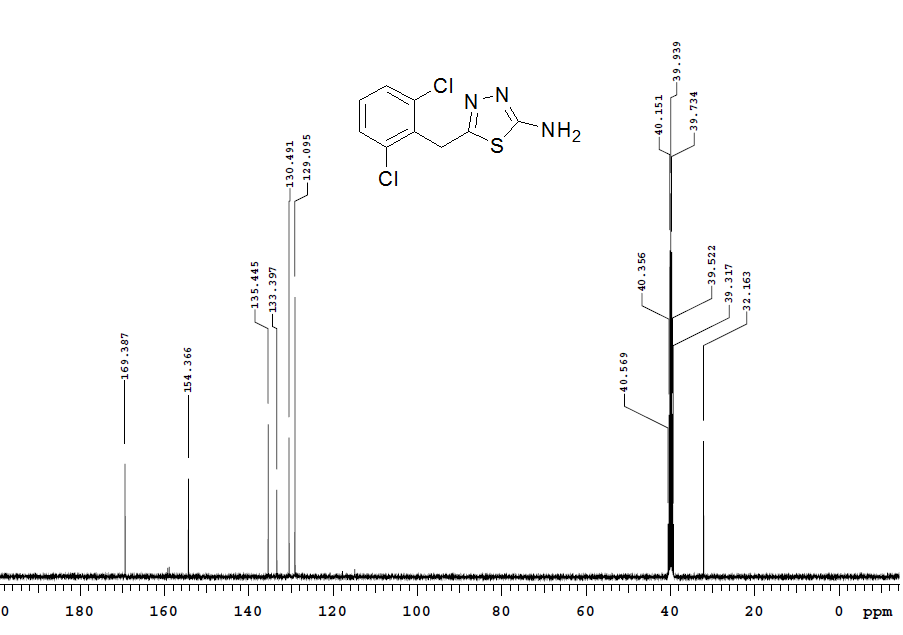


**Figure S5**. ^13^C NMR spectrum of compound **3** (DMSO-d_6_).

**
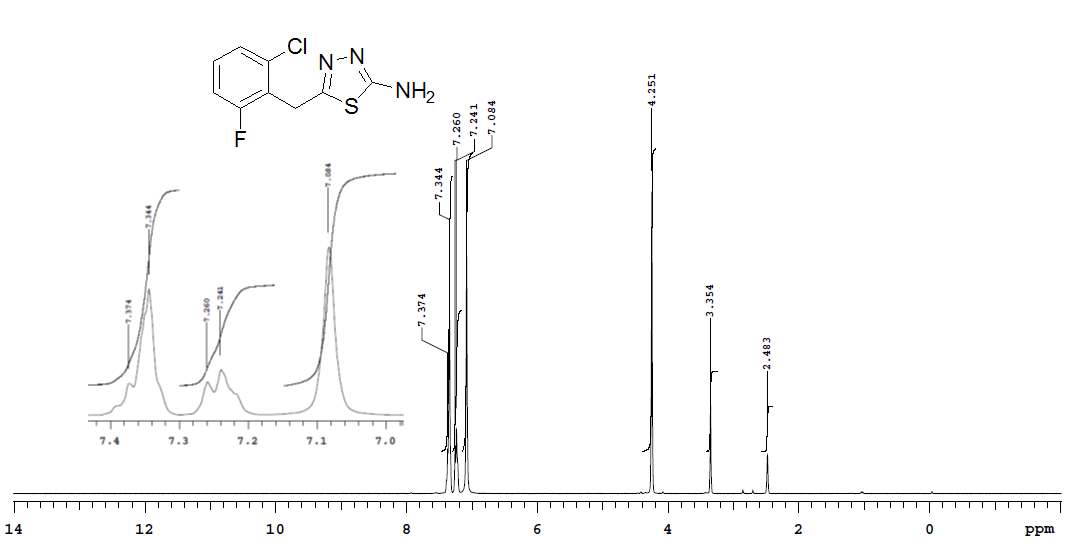
**

**Figure S6**. ^1^H NMR spectrum of compound **4** (DMSO-d_6_).

**
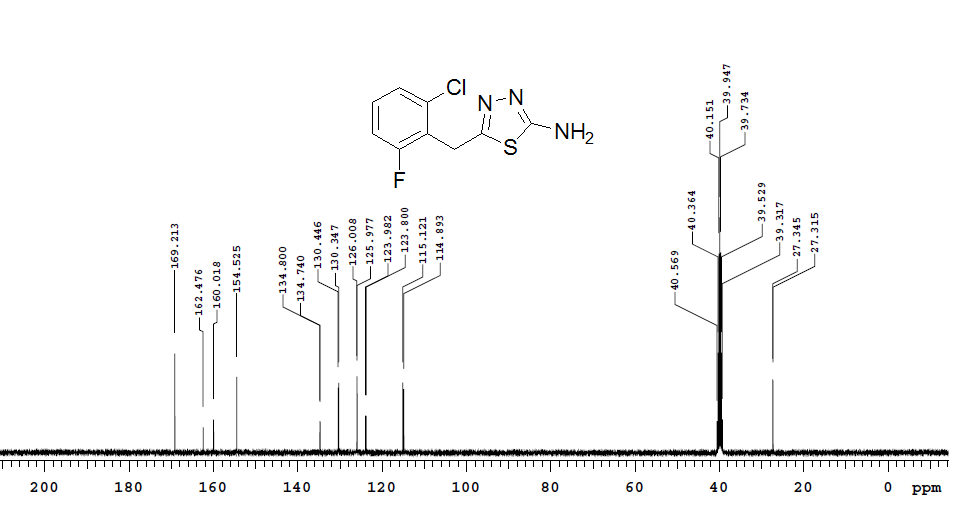
**

**Figure S7.** ^13^C NMR spectrum of compound **4** (DMSO-d_6_).


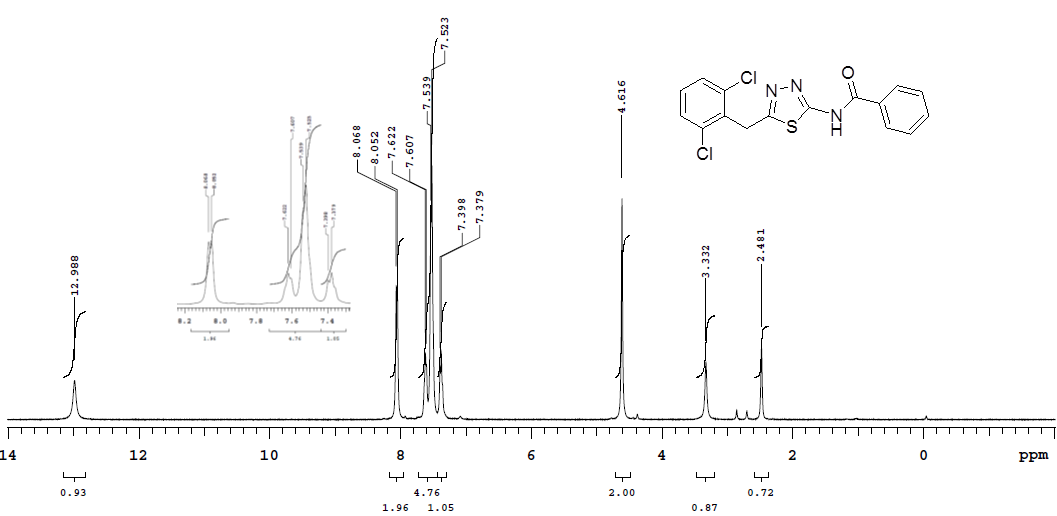


**Figure S8**. ^1^H NMR spectrum of compound **6a** (DMSO-d_6_).


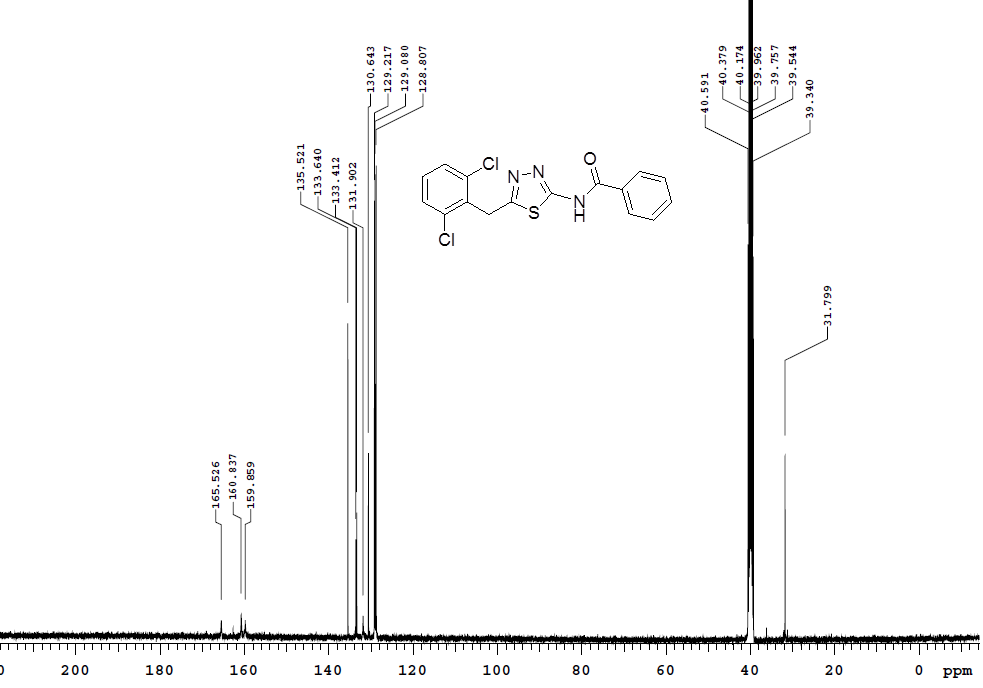


**Figure S9**. ^13^C NMR spectrum of compound **6a** (DMSO-d_6_).


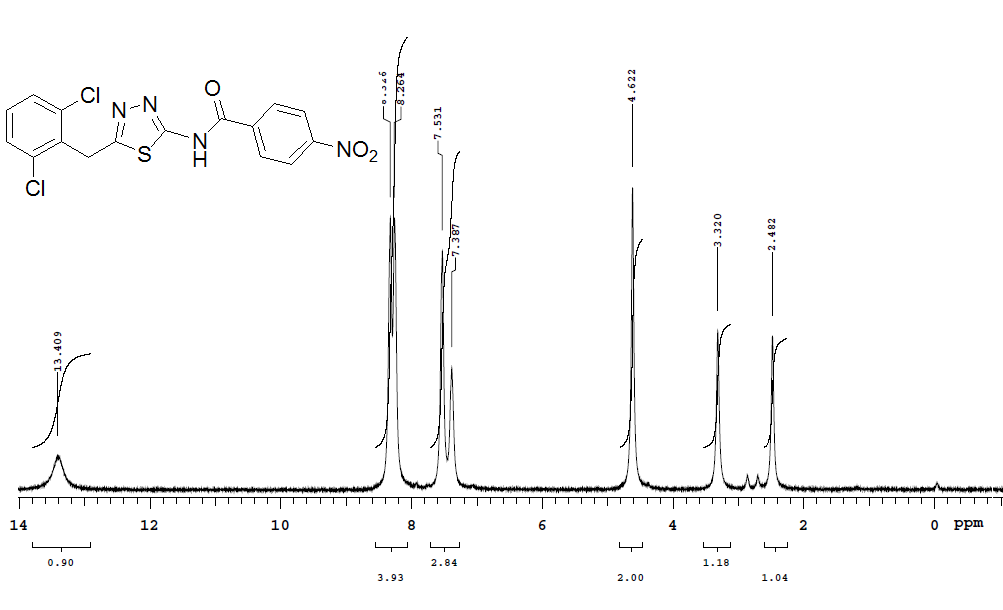


**Figure S10**. ^1^H NMR spectrum of compound **6b** (DMSO-d_6_).


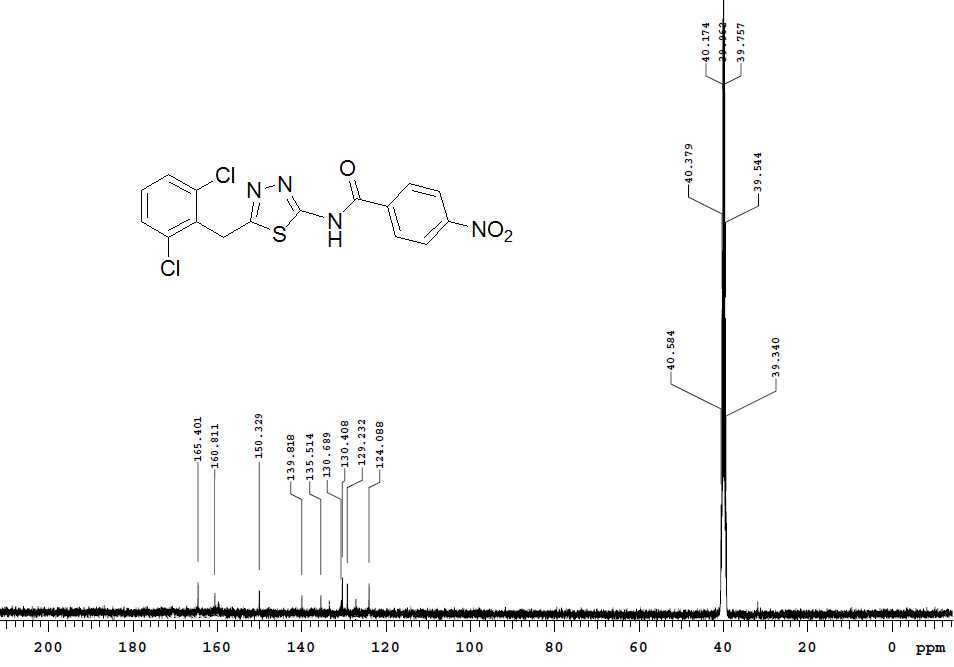


**Figure S11**. ^13^C NMR spectrum of compound **6b** (DMSO-d_6_).


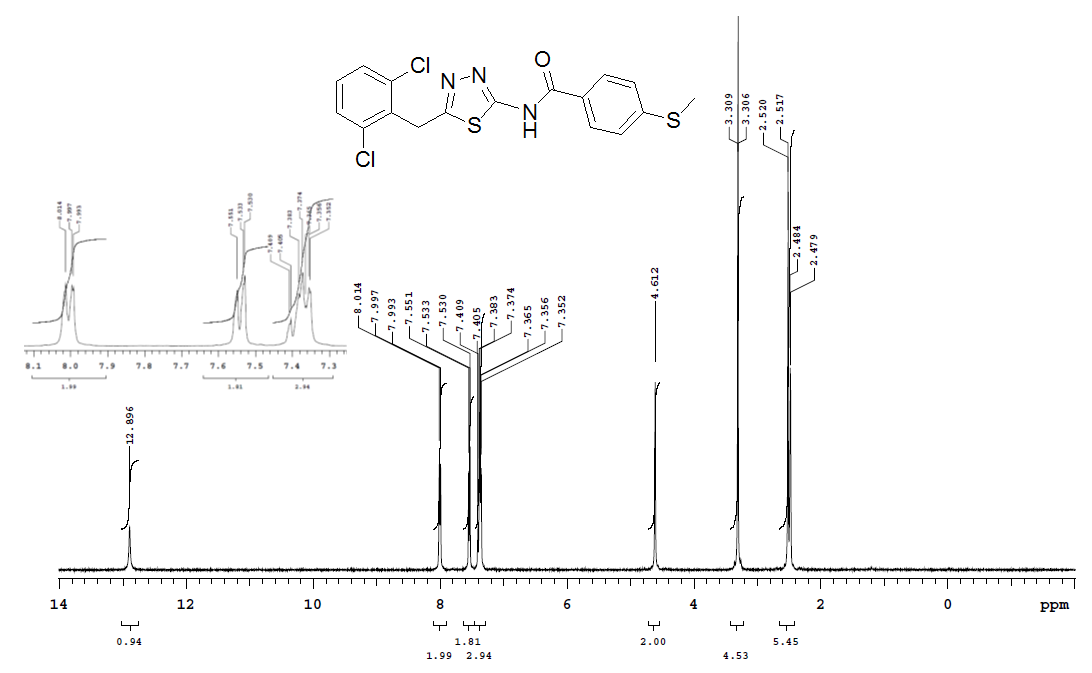


**Figure S12**. ^1^H NMR spectrum of compound **6c** (DMSO-d_6_).


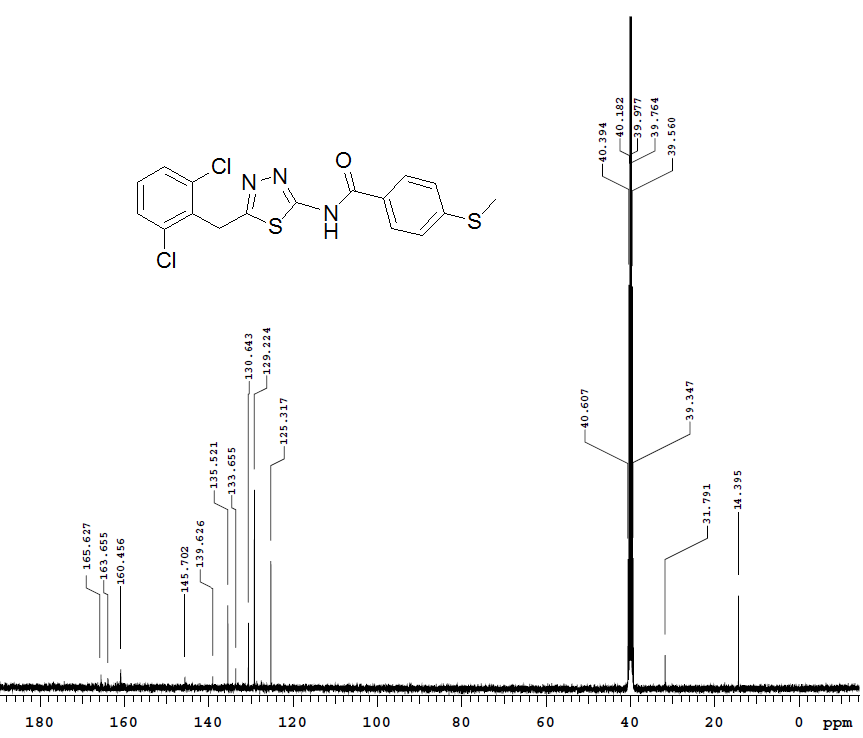


**Figure S13**. ^13^C NMR spectrum of compound **6c** (DMSO-d_6_).


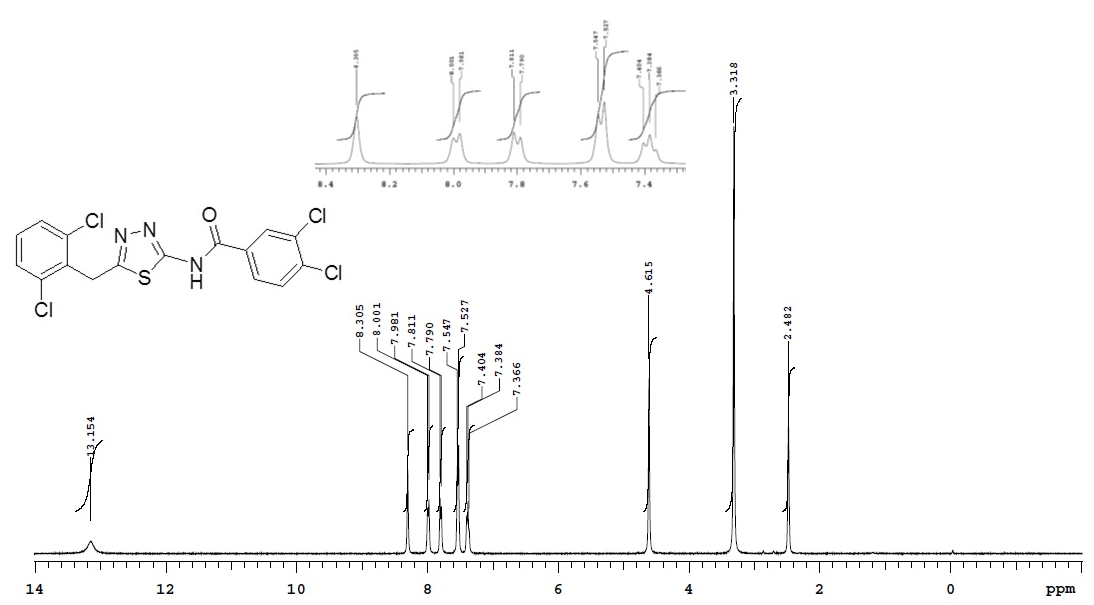


**Figure S14**. ^1^H NMR spectrum of compound **6d** (DMSO-d_6_).


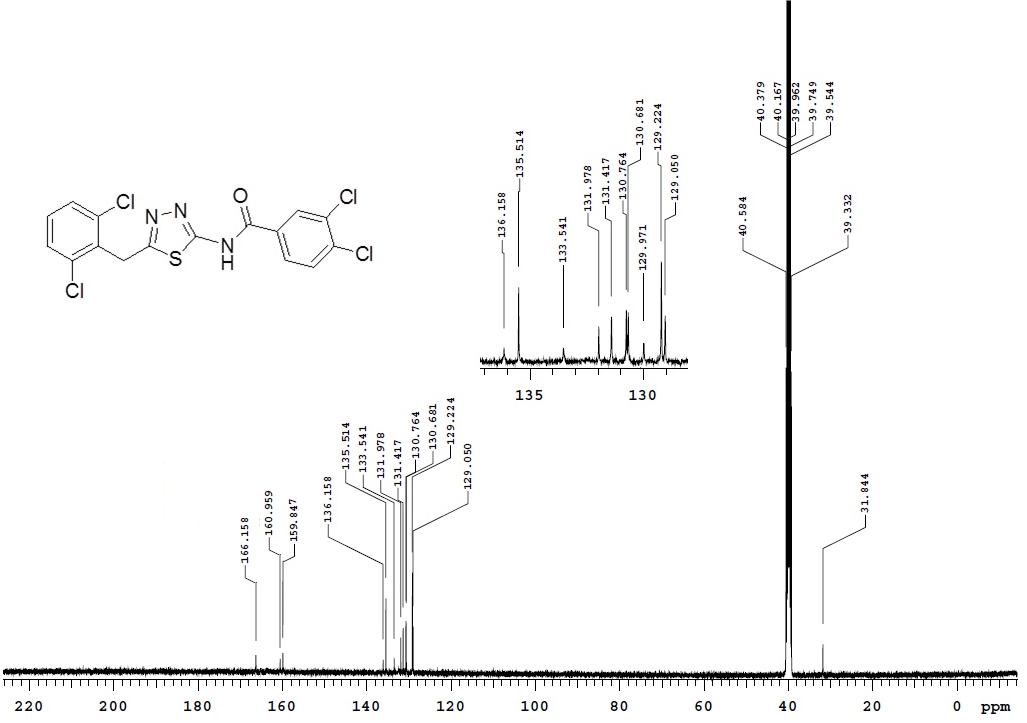


**Figure S15**. ^13^C NMR spectrum of compound **6d** (DMSO-d_6_).


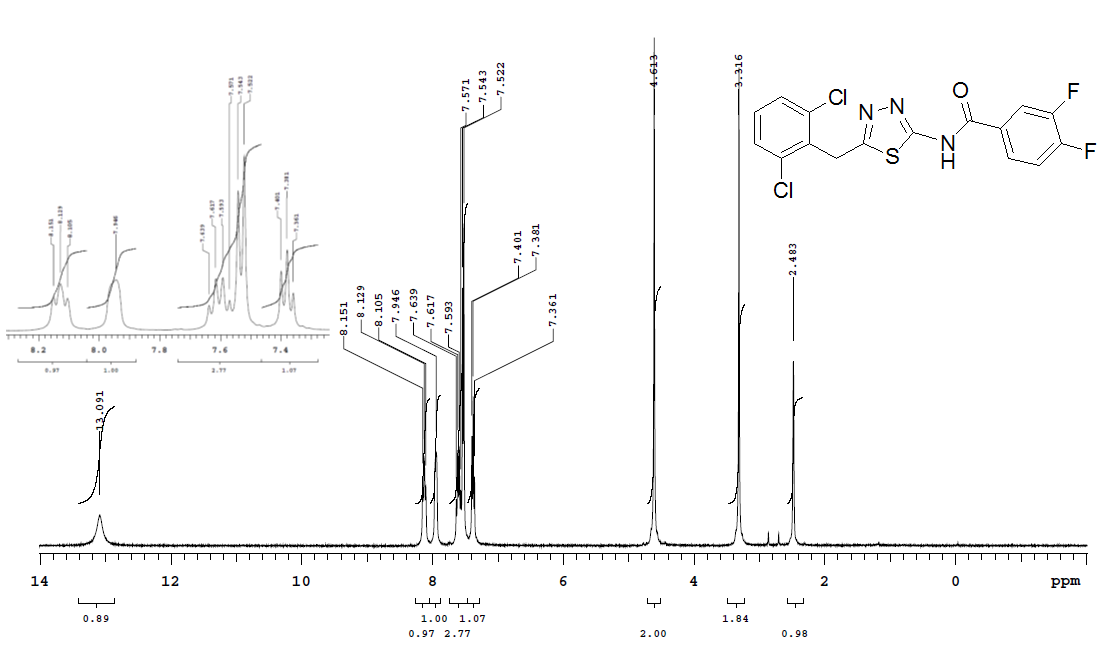


**Figure S16**. ^1^H NMR spectrum of compound **6e** (DMSO-d_6_).


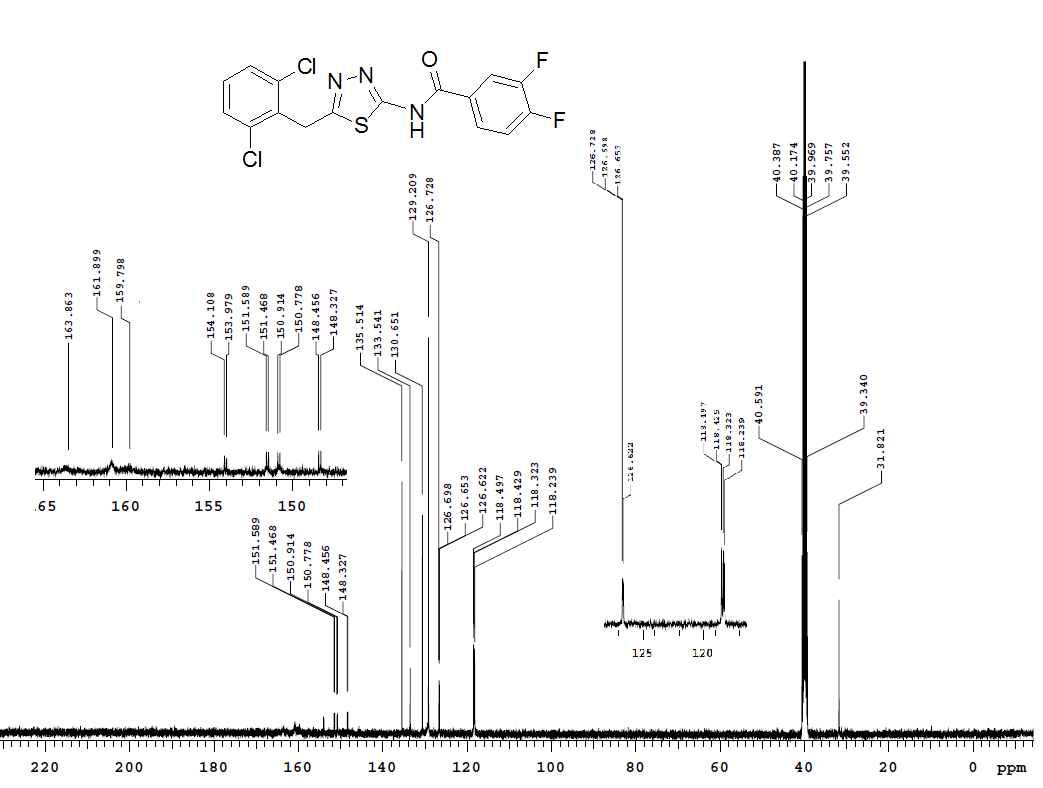


**Figure S17**. ^13^C NMR spectrum of compound **6e** (DMSO-d_6_).


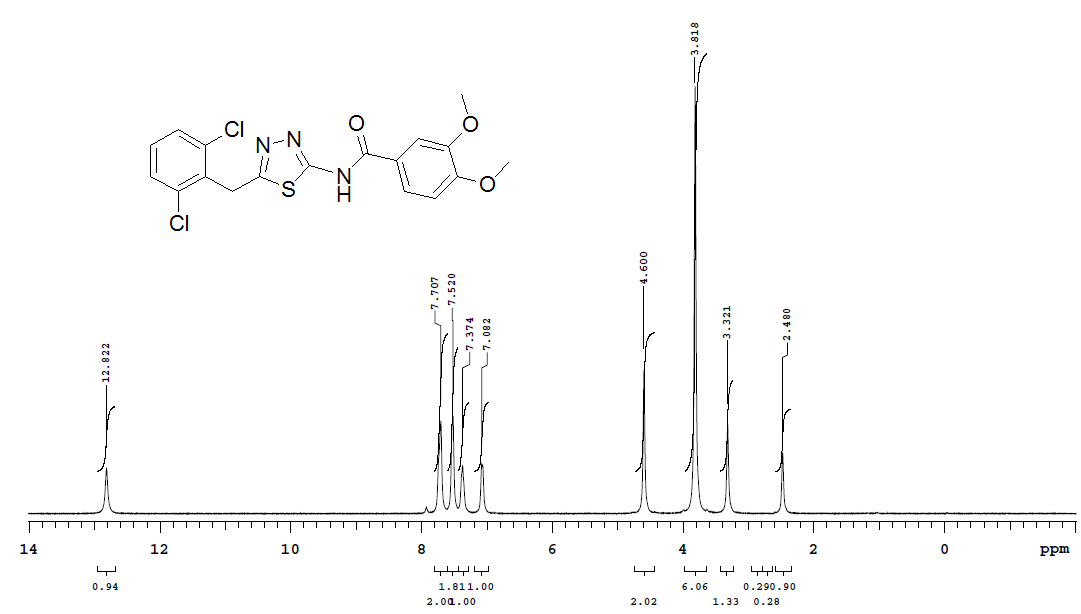


**Figure S18**. ^1^H NMR spectrum of compound **6f** (DMSO-d_6_).


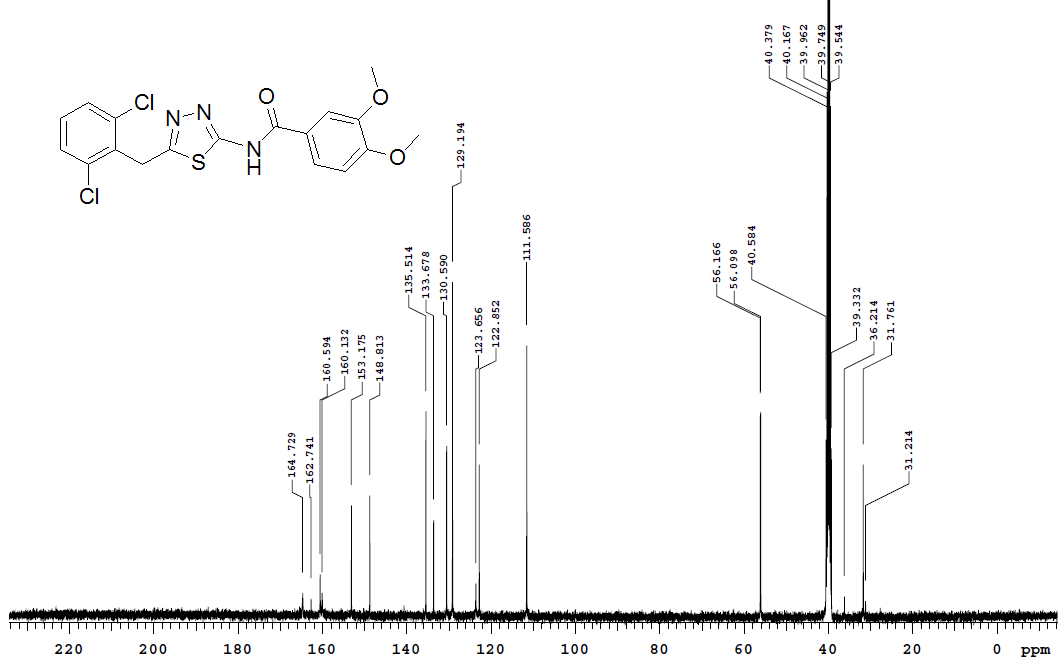


**Figure S19**. ^13^C NMR spectrum of compound **6f** (DMSO-d_6_).


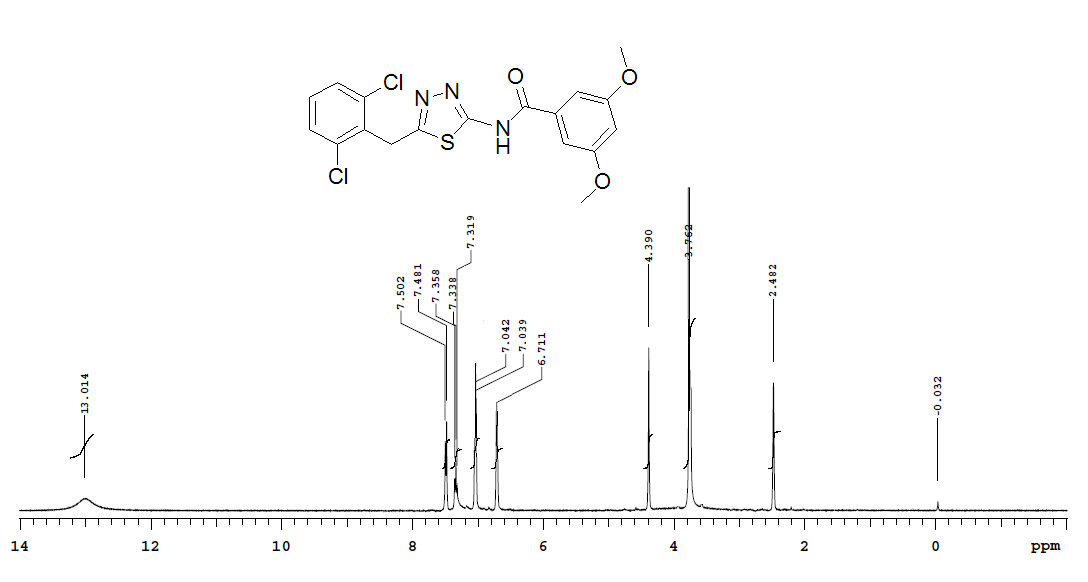


**Figure S20**. ^1^H NMR spectrum of compound **6g** (DMSO-d_6_).


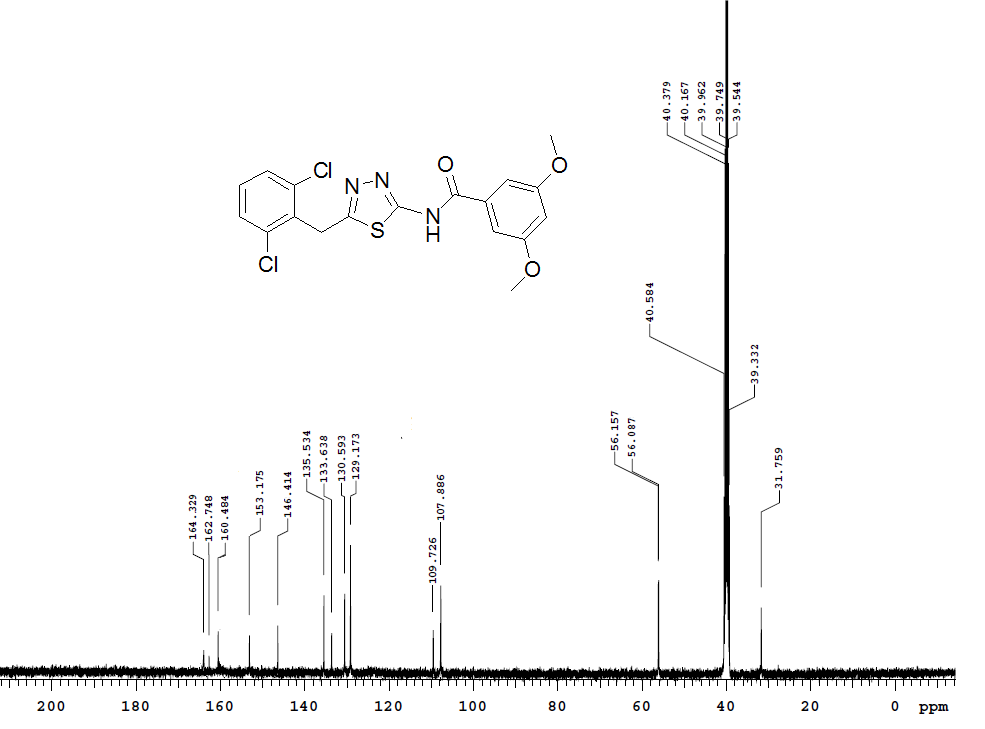


**Figure S21**. ^13^C NMR spectrum of compound **6g** (DMSO-d_6_).


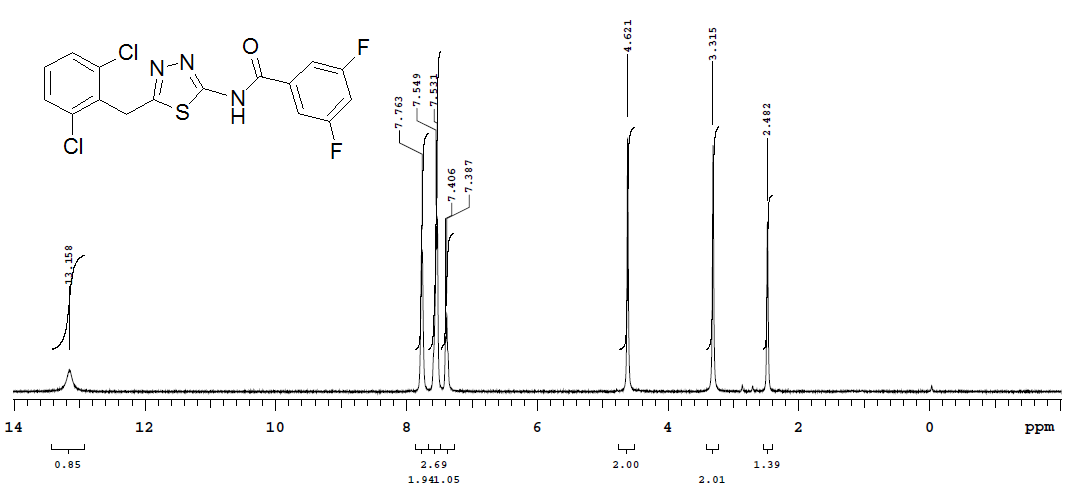


**Figure S22**. ^1^H NMR spectrum of compound **6h** (DMSO-d_6_).


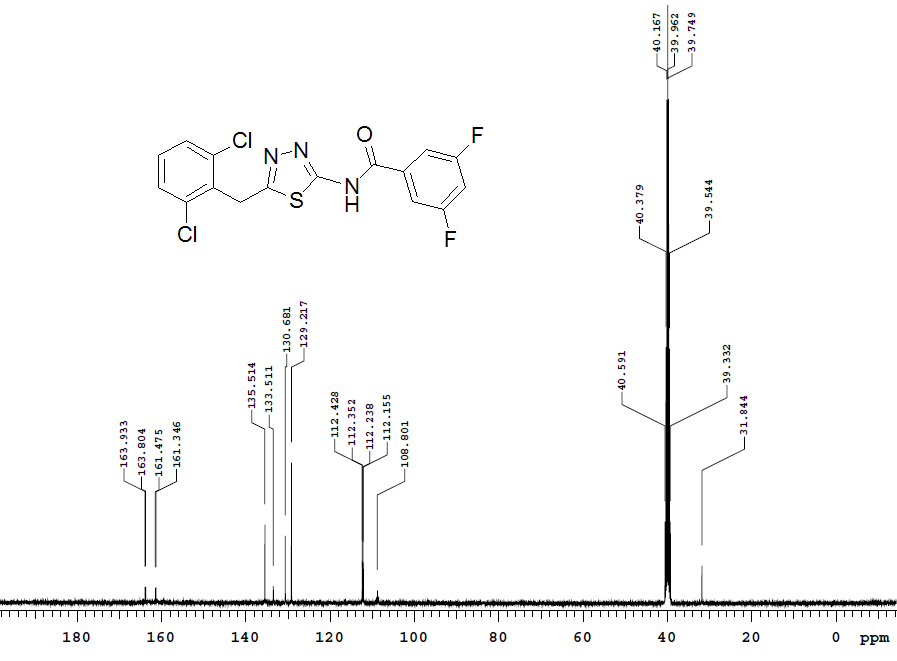


**Figure S23**. ^13^C NMR spectrum of compound **6h** (DMSO-d_6_).


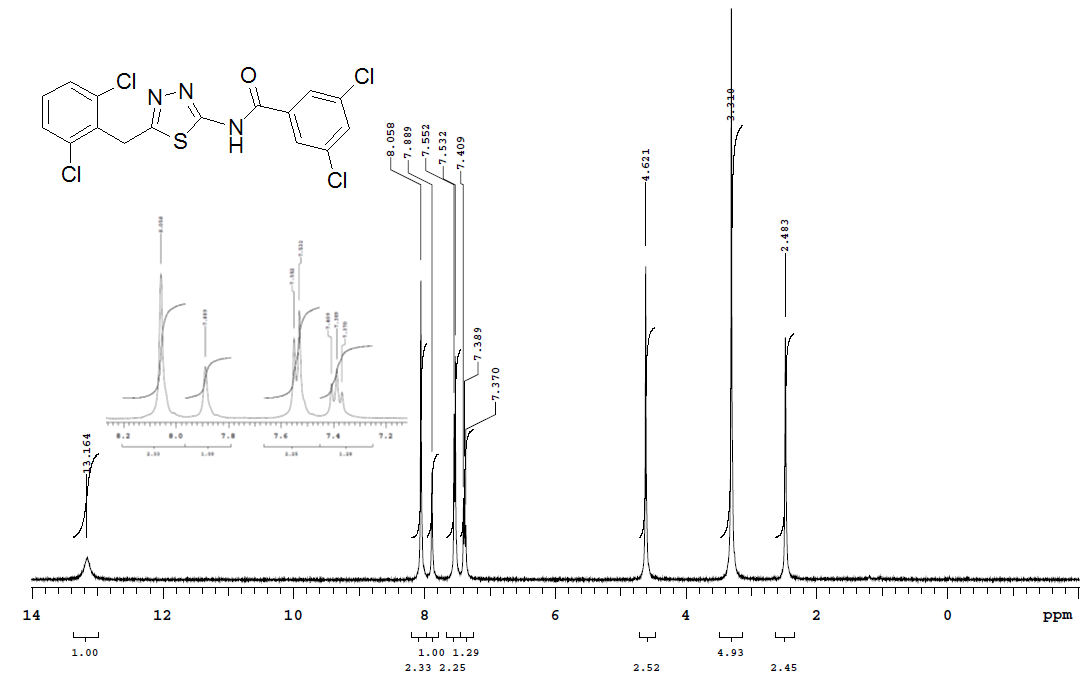


**Figure S24**. ^1^H NMR spectrum of compound **6i** (DMSO-d_6_).


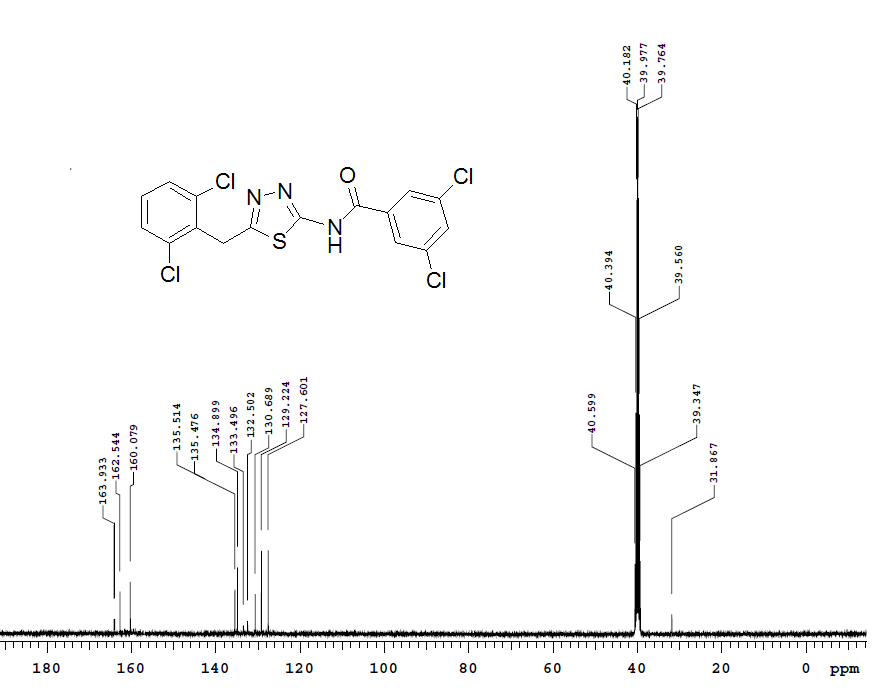


**Figure S25**. ^13^C NMR spectrum of compound **6i** (DMSO-d_6_).


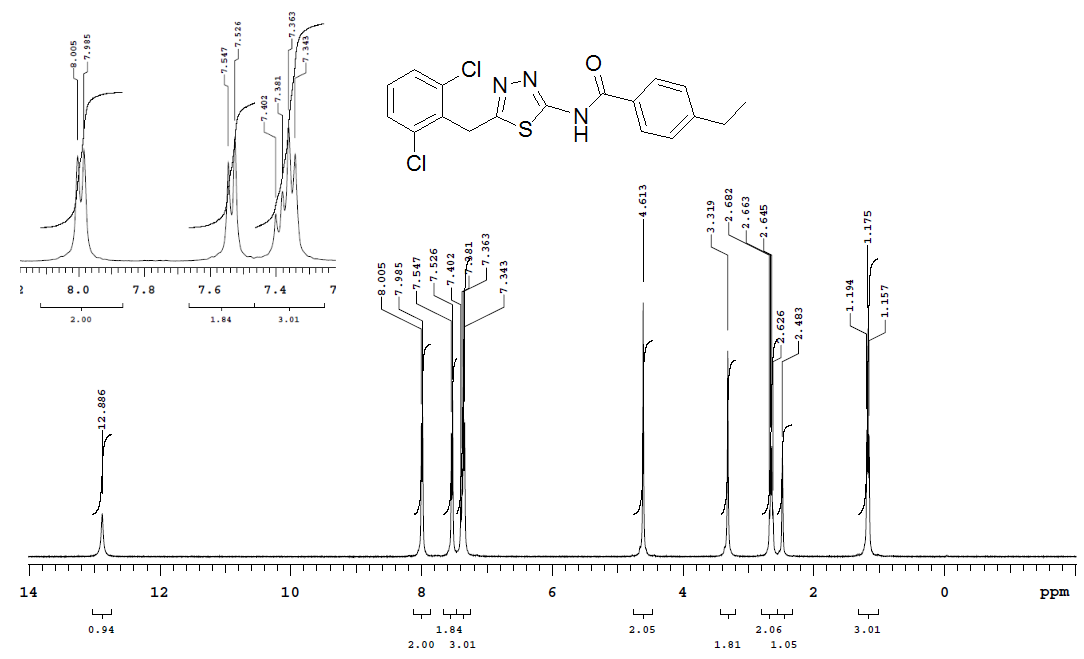


**Figure S26**. ^1^H NMR spectrum of compound **6j** (DMSO-d_6_).


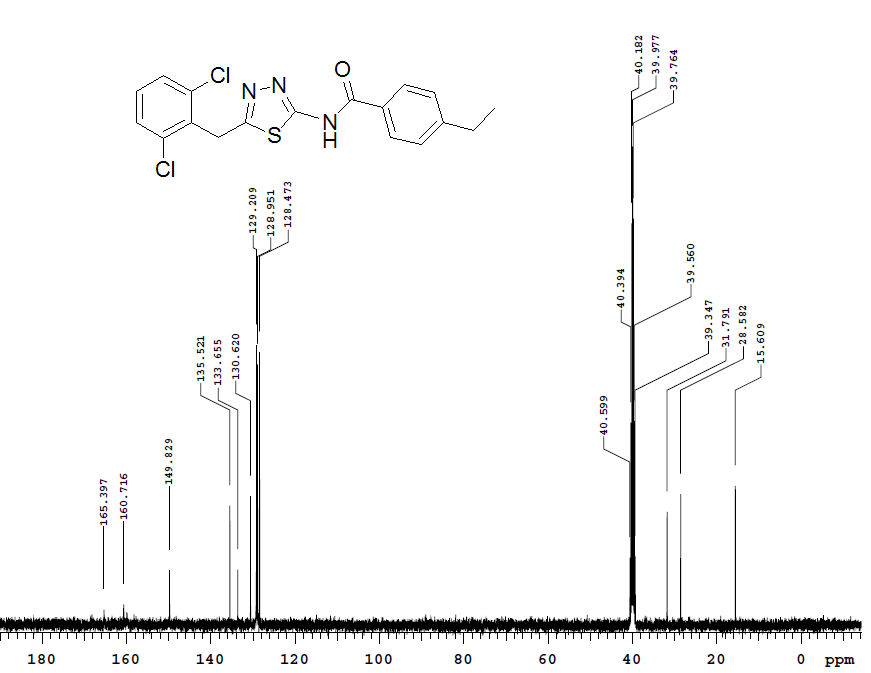


**Figure S27**. ^13^C NMR spectrum of compound **6j** (DMSO-d_6_).


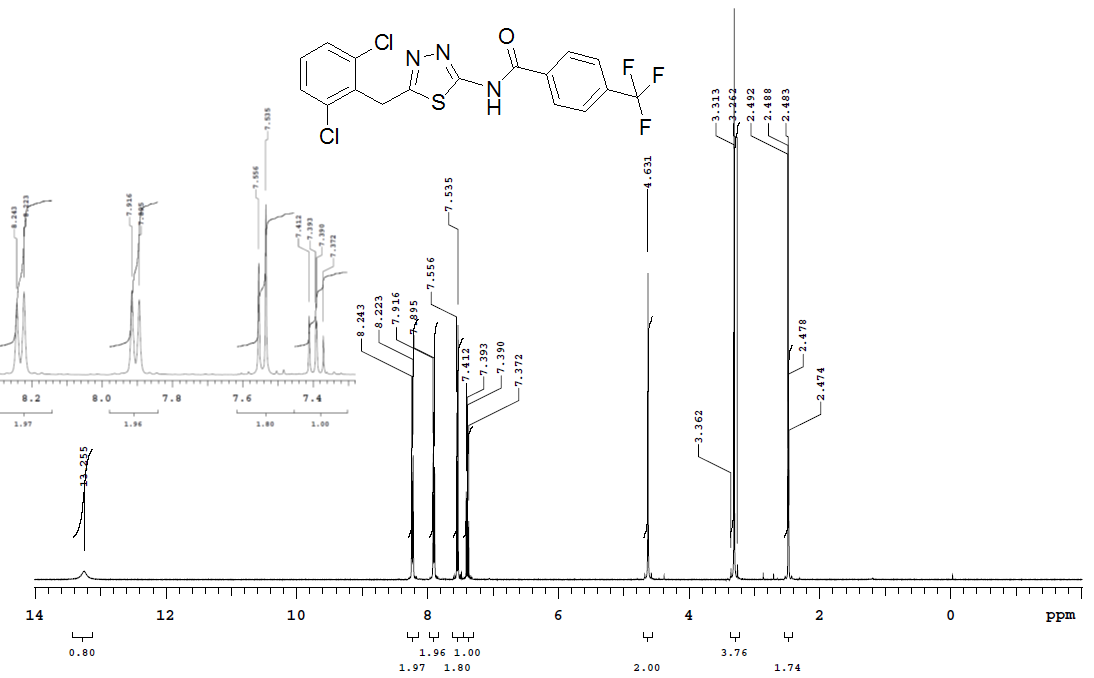


**Figure S28**. ^1^H NMR spectrum of compound **6k** (DMSO-d_6_).


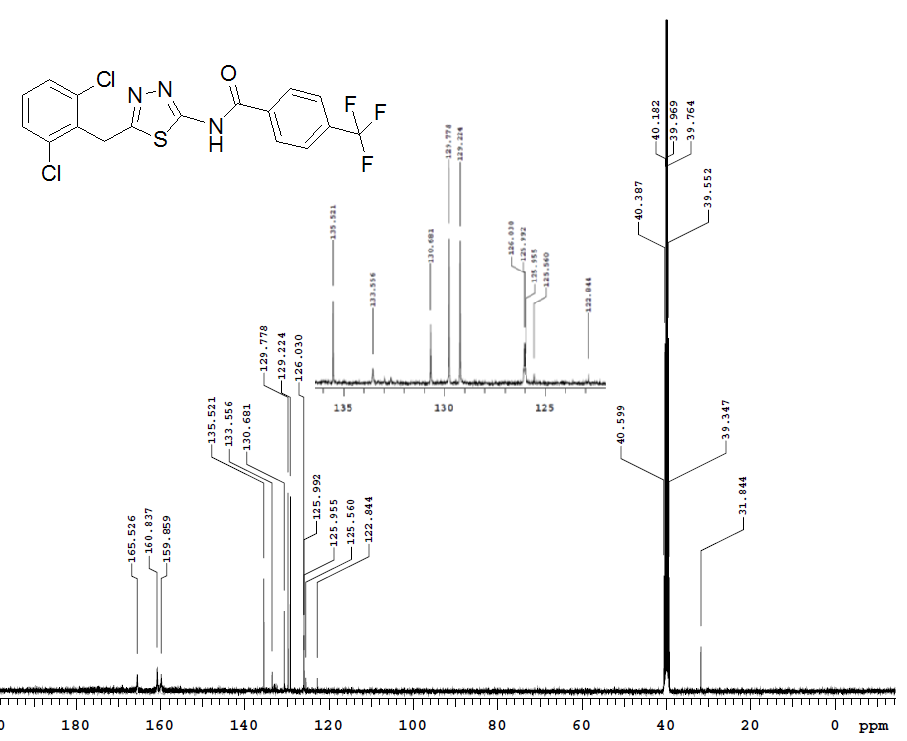


**Figure S29**. ^13^C NMR spectrum of compound **6k** (DMSO-d_6_).

**
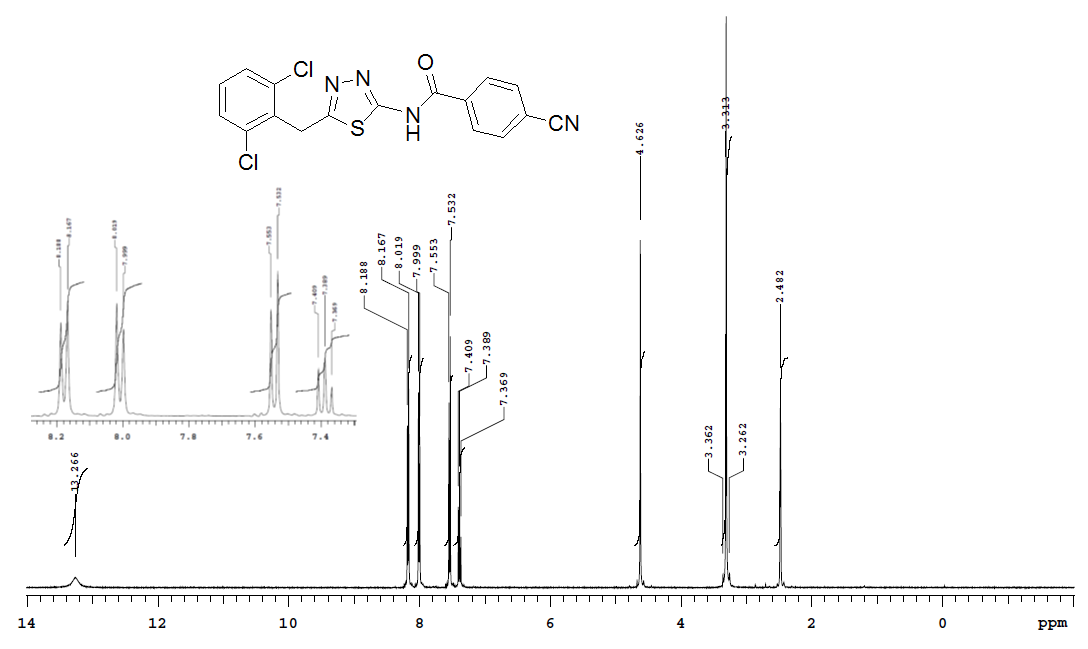
**

**Figure S30**. ^1^H NMR spectrum of compound **6l** (DMSO-d_6_).

**
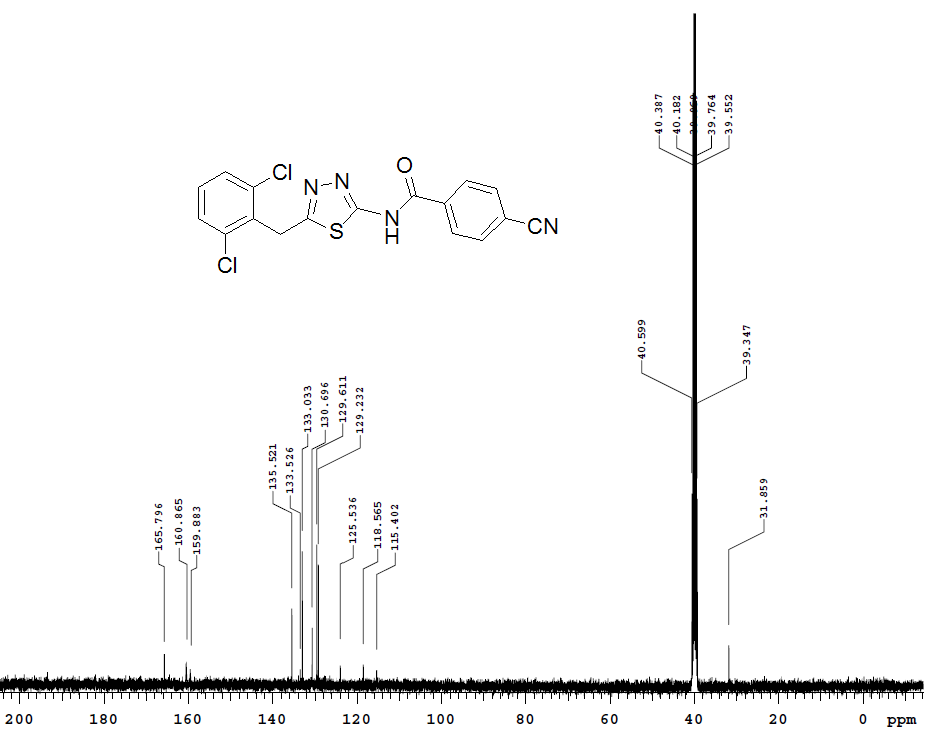
**

**Figure S31**. ^13^C NMR spectrum of compound **6l** (DMSO-d_6_).


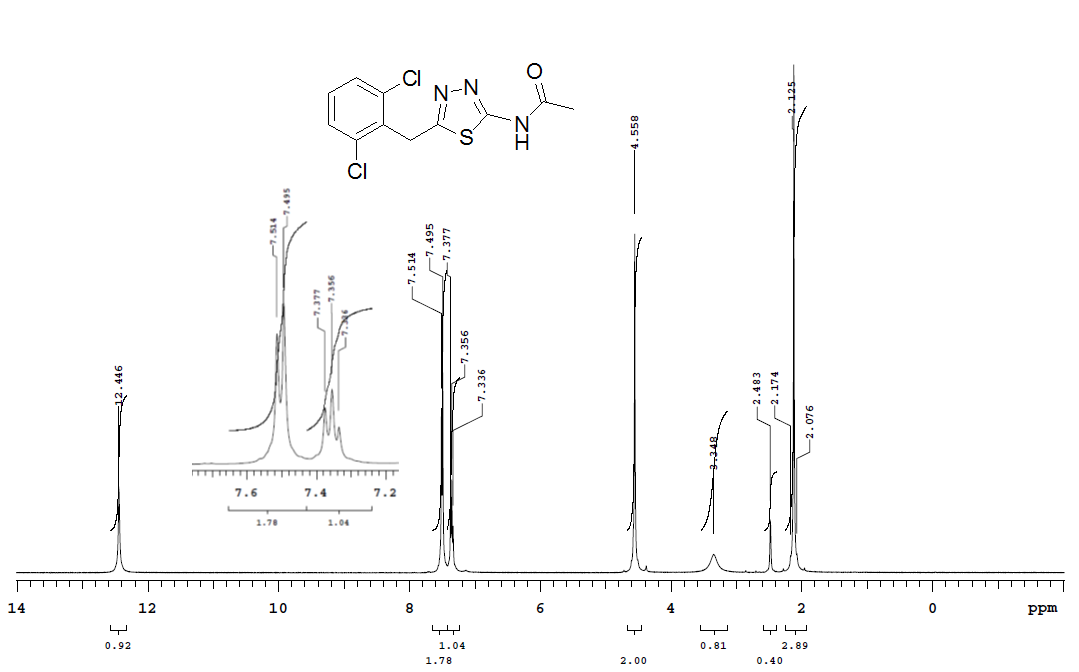


**Figure S32**. ^1^H NMR spectrum of compound **6m** (DMSO-d_6_).


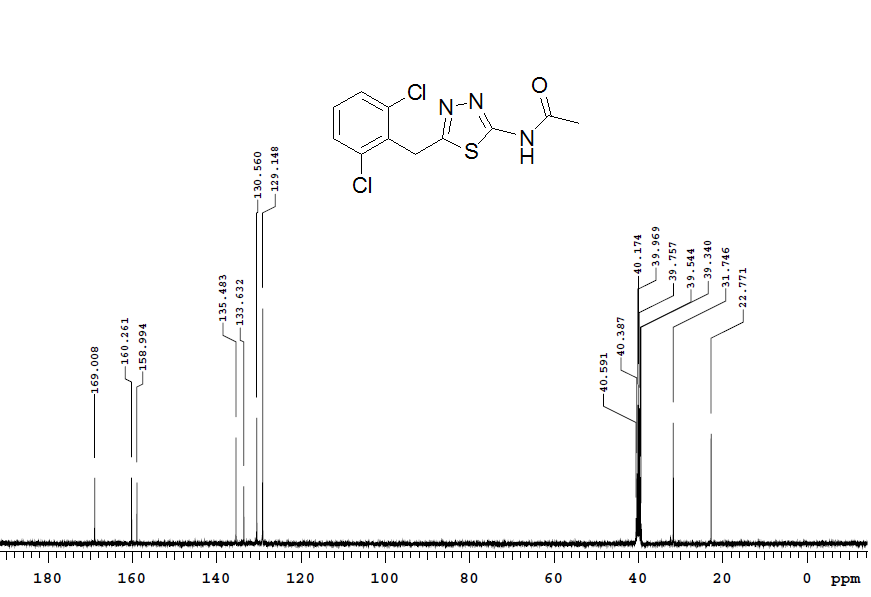


**Figure S33**. ^13^C NMR spectrum of compound **6m** (DMSO-d_6_).


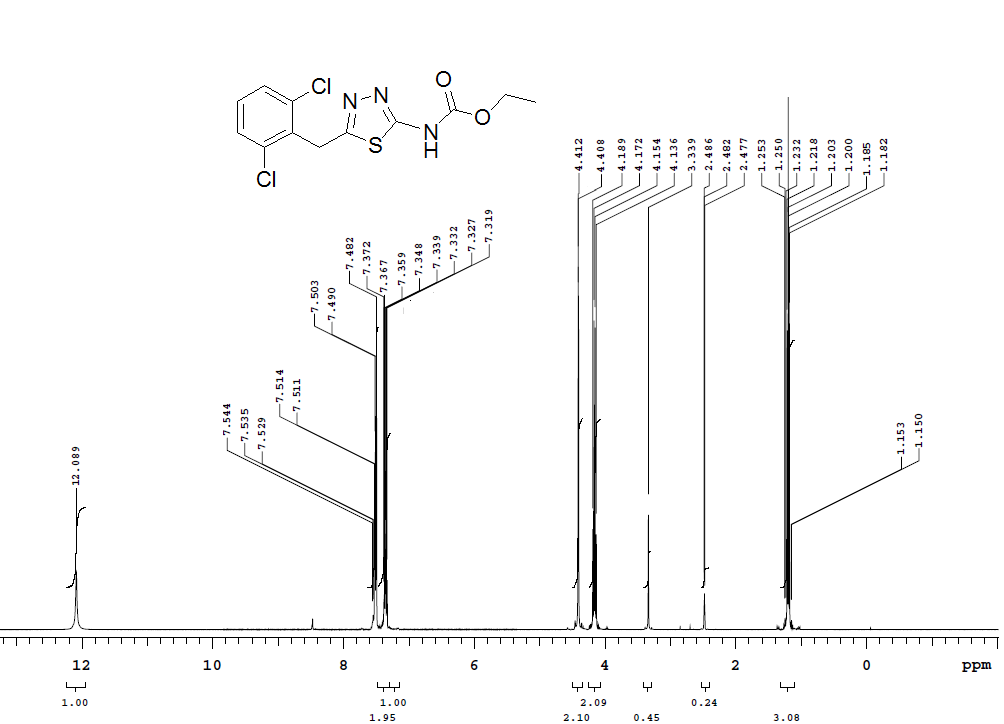


**Figure S34**. ^1^H NMR spectrum of compound **6n** (DMSO-d_6_).


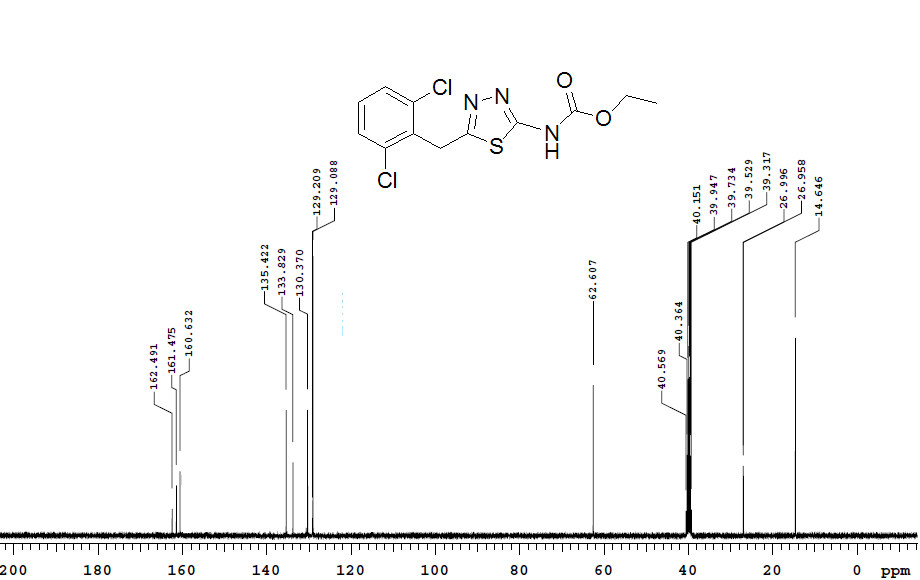


**Figure S35**. ^13^C NMR spectrum of compound **6n** (DMSO-d_6_).

**
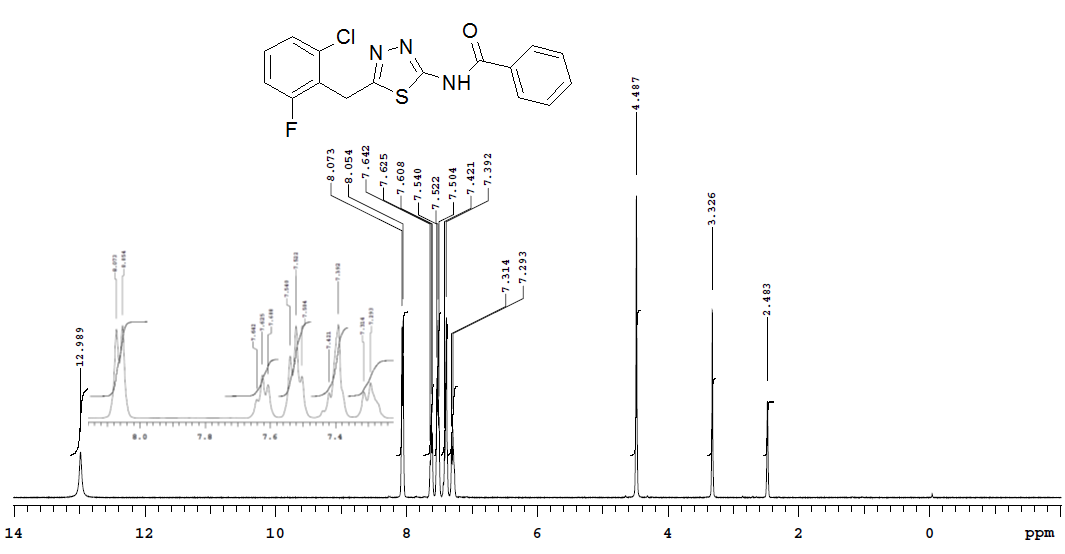
**

**Figure S36**. ^1^H NMR spectrum of compound **7a** (DMSO-d_6_).

**
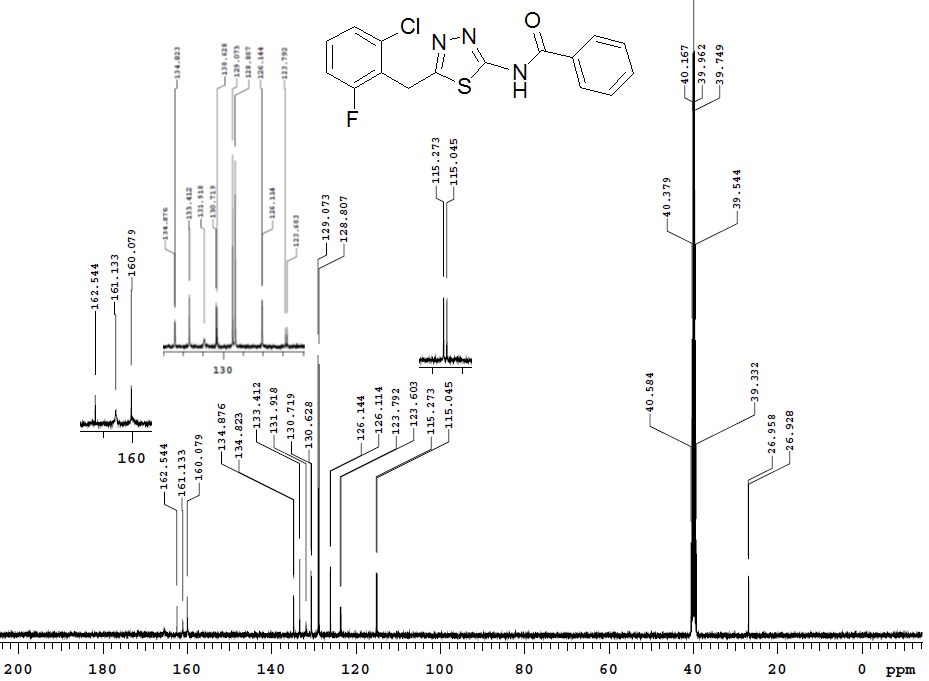
**

**Figure S37**. ^13^C NMR spectrum of compound **7a** (DMSO-d_6_).

**
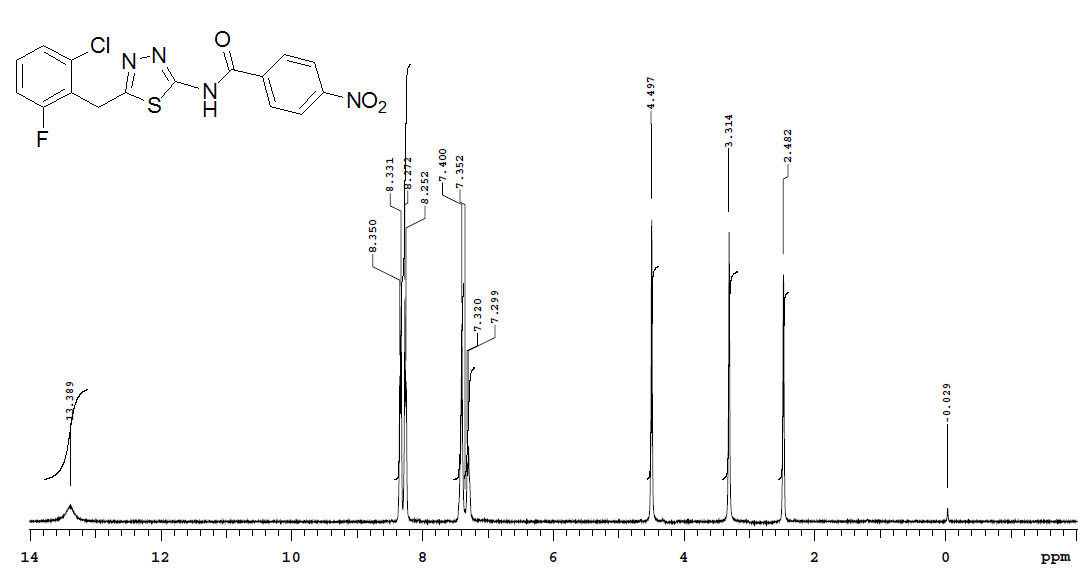
**

**Figure S38**. ^1^H NMR spectrum of compound **7b** (DMSO-d_6_).

**
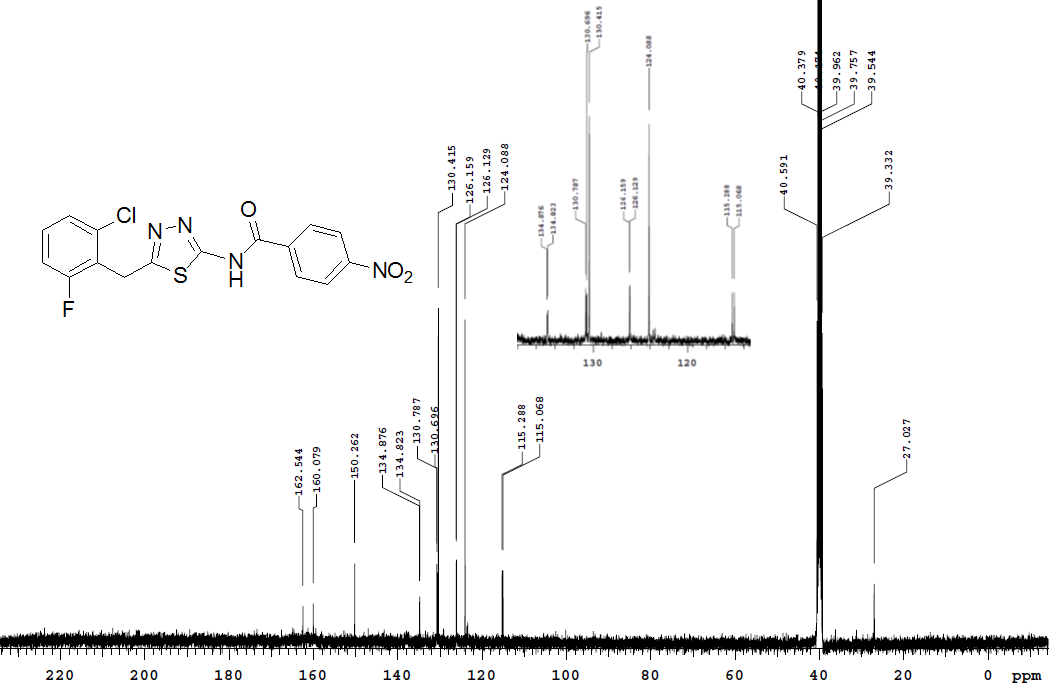
**

**Figure S39**. ^13^C NMR spectrum of compound **7b** (DMSO-d_6_).


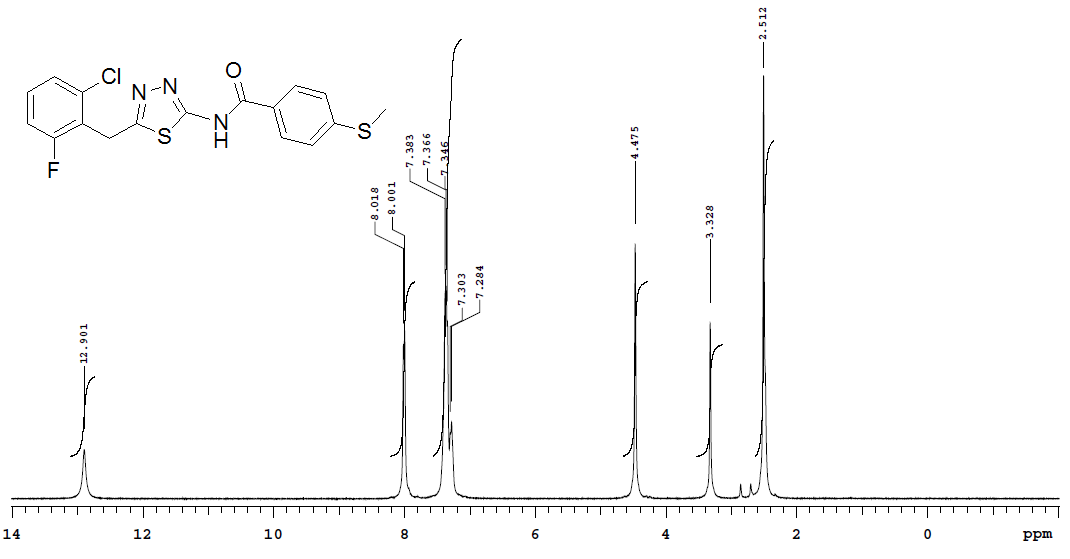


**Figure S40**. ^1^H NMR spectrum of compound **7c** (DMSO-d_6_).

**
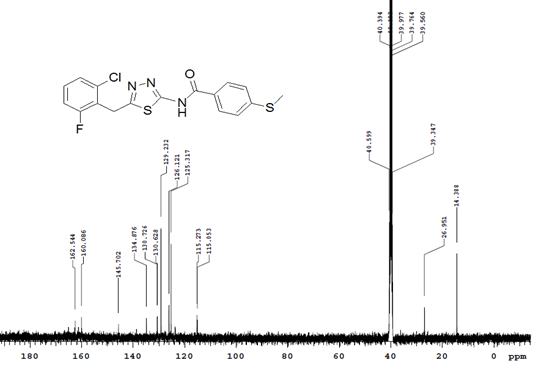
**

**Figure S41**. ^13^C NMR spectrum of compound **7c** (DMSO-d_6_).

**
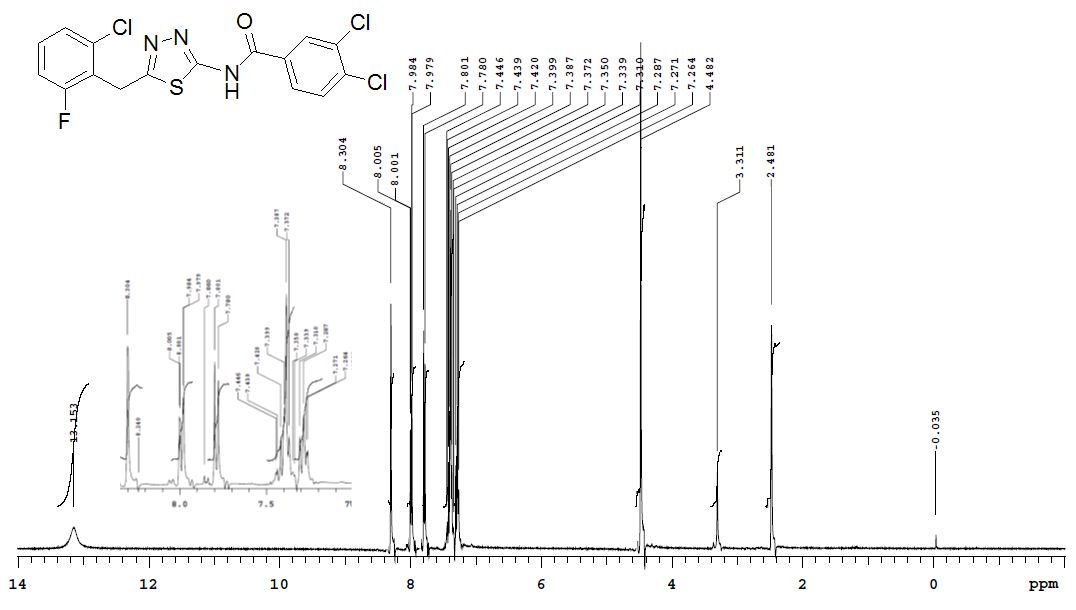
**

**Figure 42**. ^1^H NMR spectrum of compound **7d** (DMSO-d_6_).

**
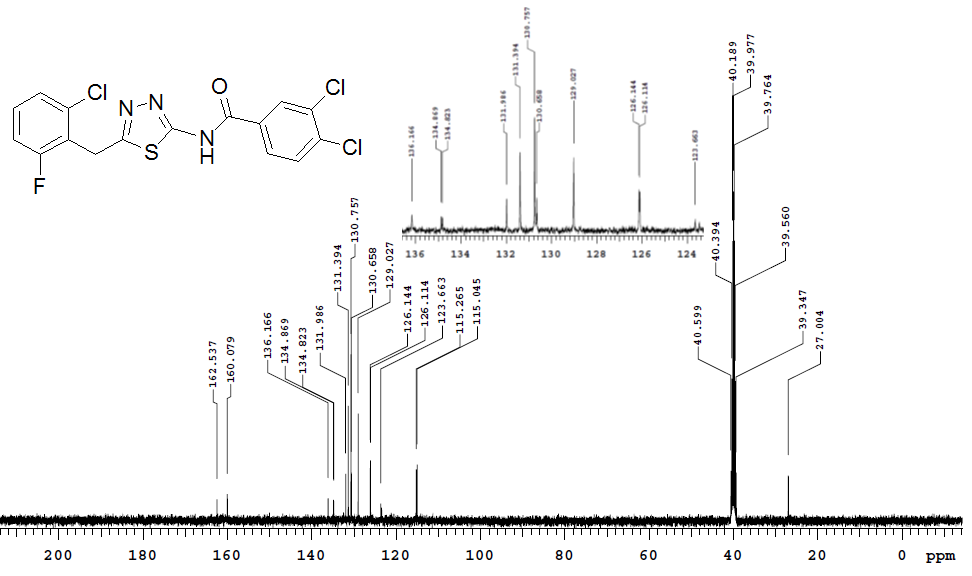
**

**Figure S43**. ^13^C NMR spectrum of compound **7d** (DMSO-d_6_).

**
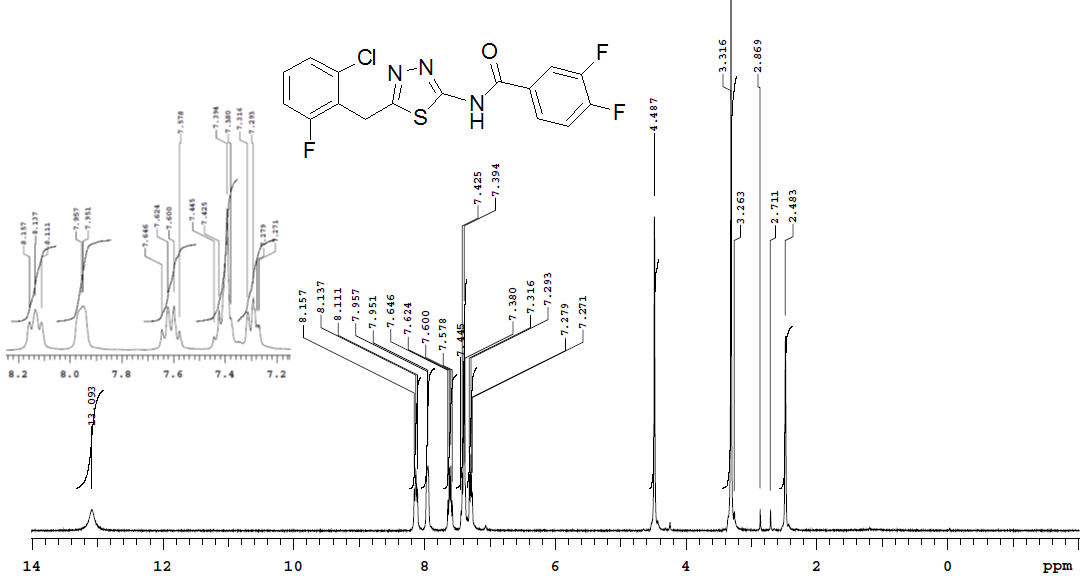
**

**Figure S44**. ^1^H NMR spectrum of compound **7e** (DMSO-d_6_).

**
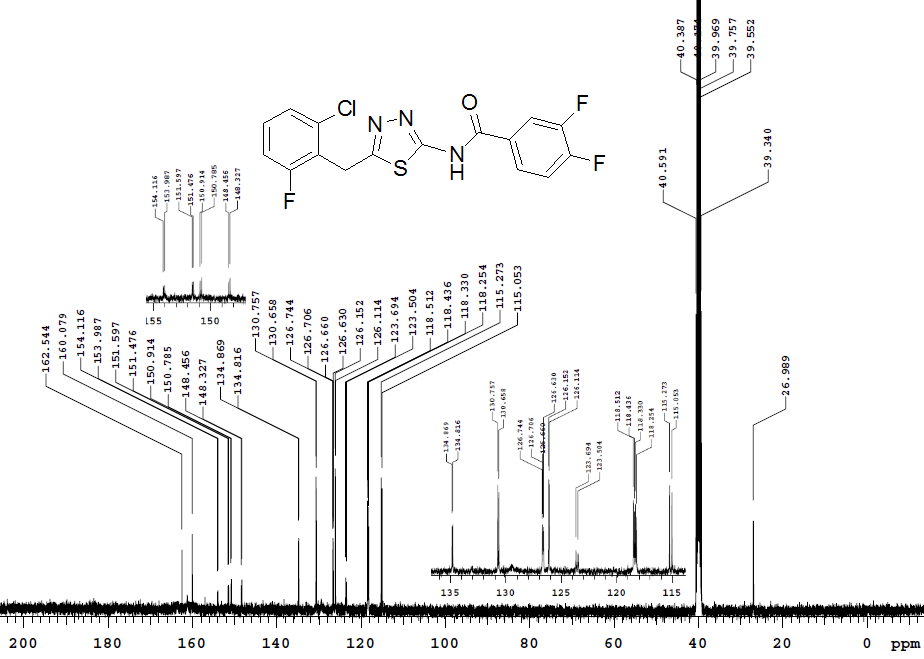
**

**Figure S45**. ^13^C NMR spectrum of compound **7e** (DMSO-d_6_).

**
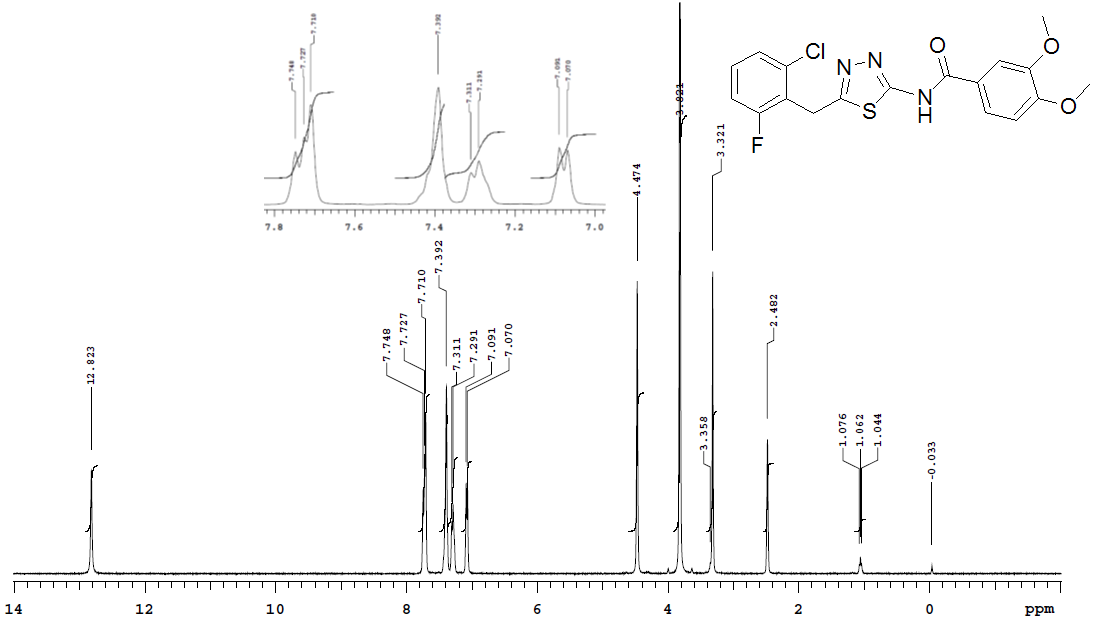
**

**Figure S46**. ^1^H NMR spectrum of compound **7f** (DMSO-d_6_).

**
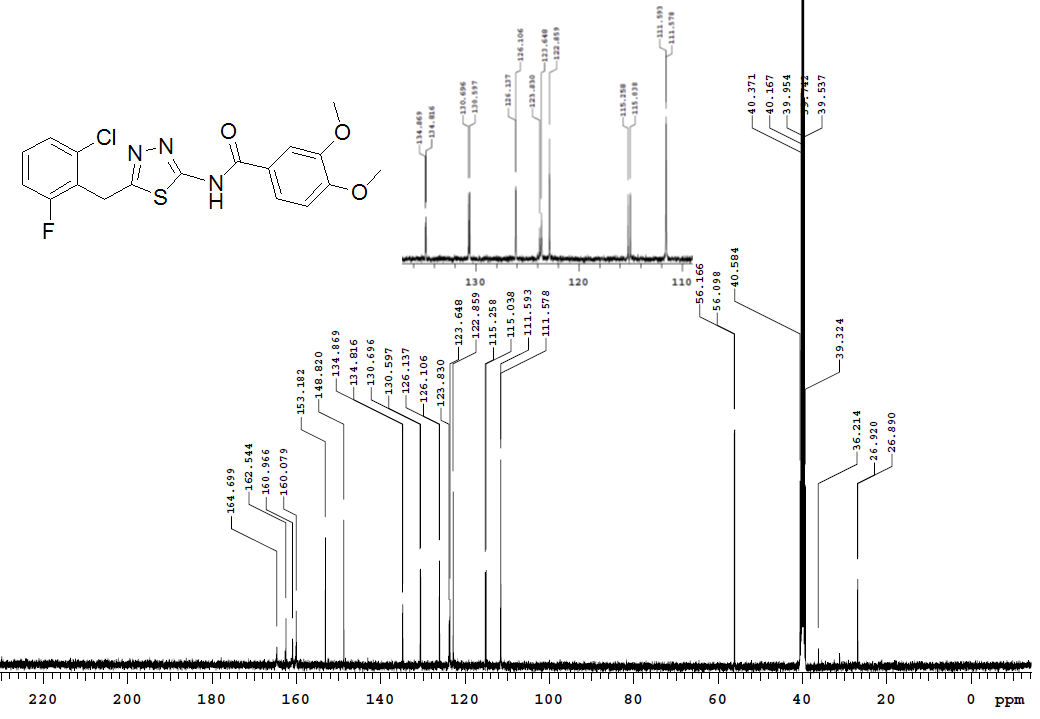
**

**Figure S47**. ^13^C NMR spectrum of compound **7f** (DMSO-d_6_).

**
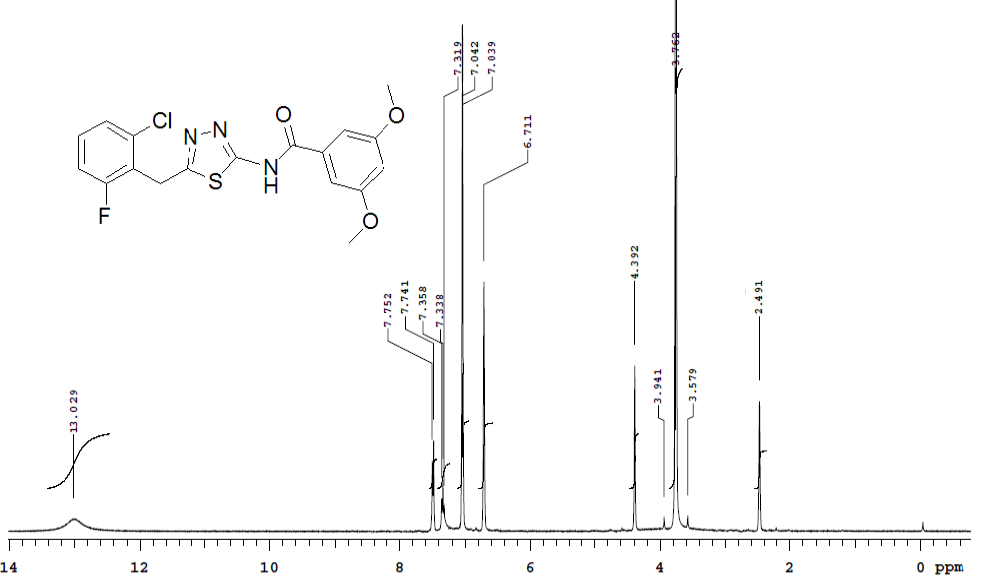
**

**Figure S48**. ^1^H NMR spectrum of compound **7g** (DMSO-d_6_).


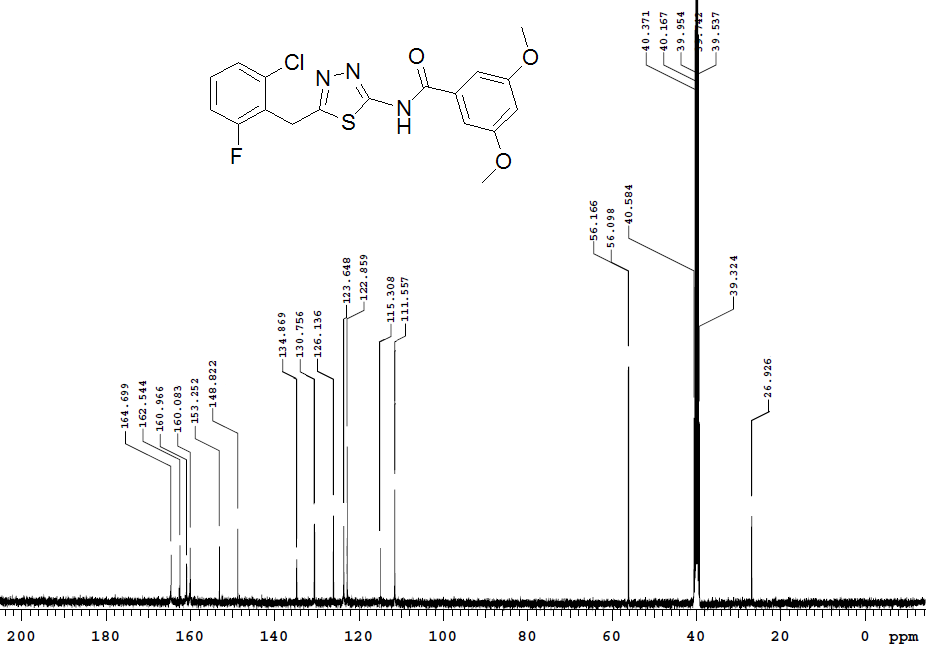


**Figure S49**. ^13^C NMR spectrum of compound **7g** (DMSO-d_6_).

**
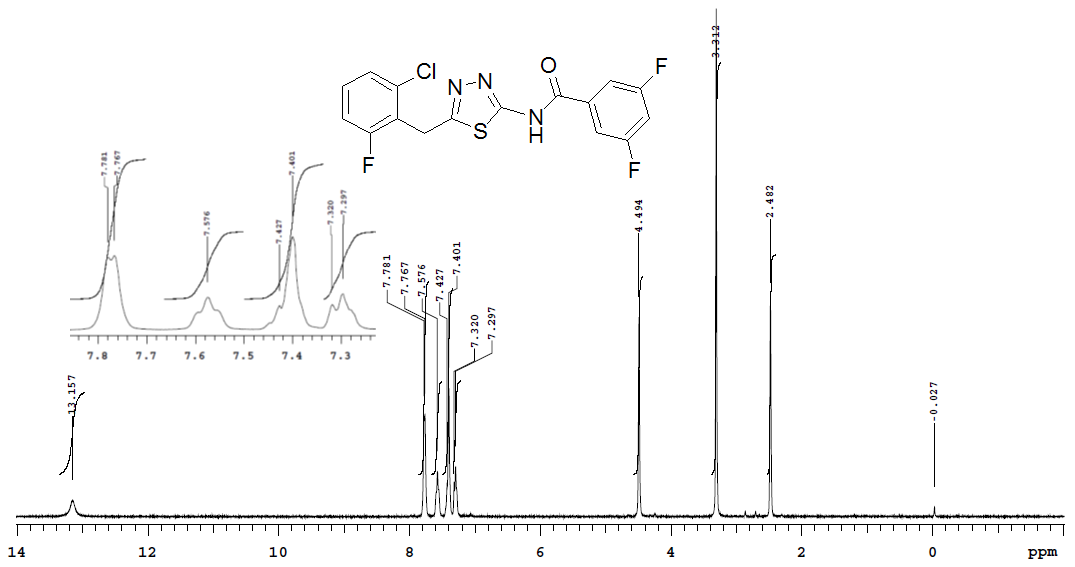
**

**Figure S50**. ^1^H NMR spectrum of compound **7h** (DMSO-d_6_).


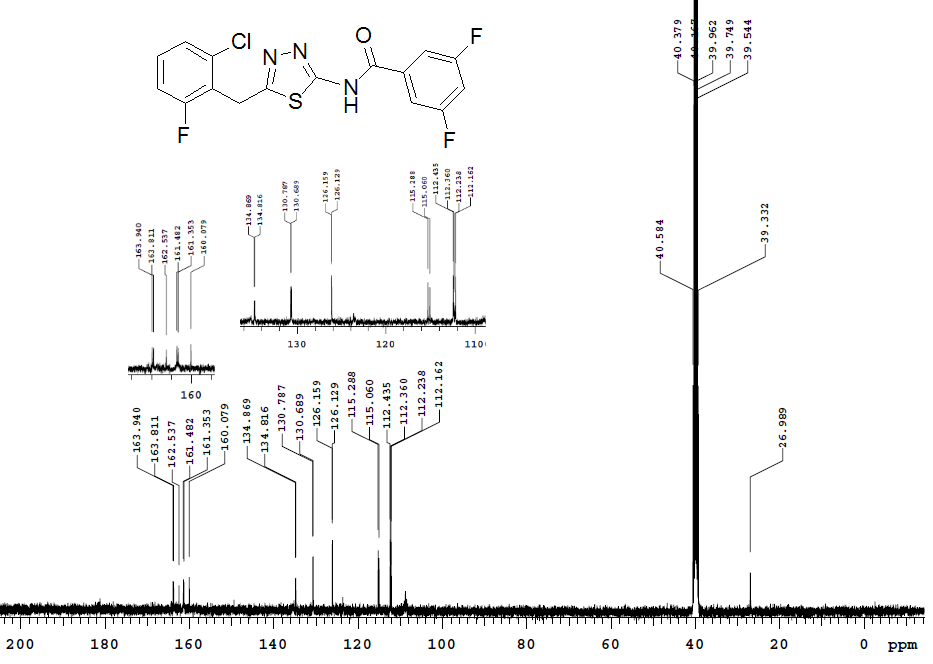


**Figure S51**. ^13^C NMR spectrum of compound **7h** (DMSO-d_6_).


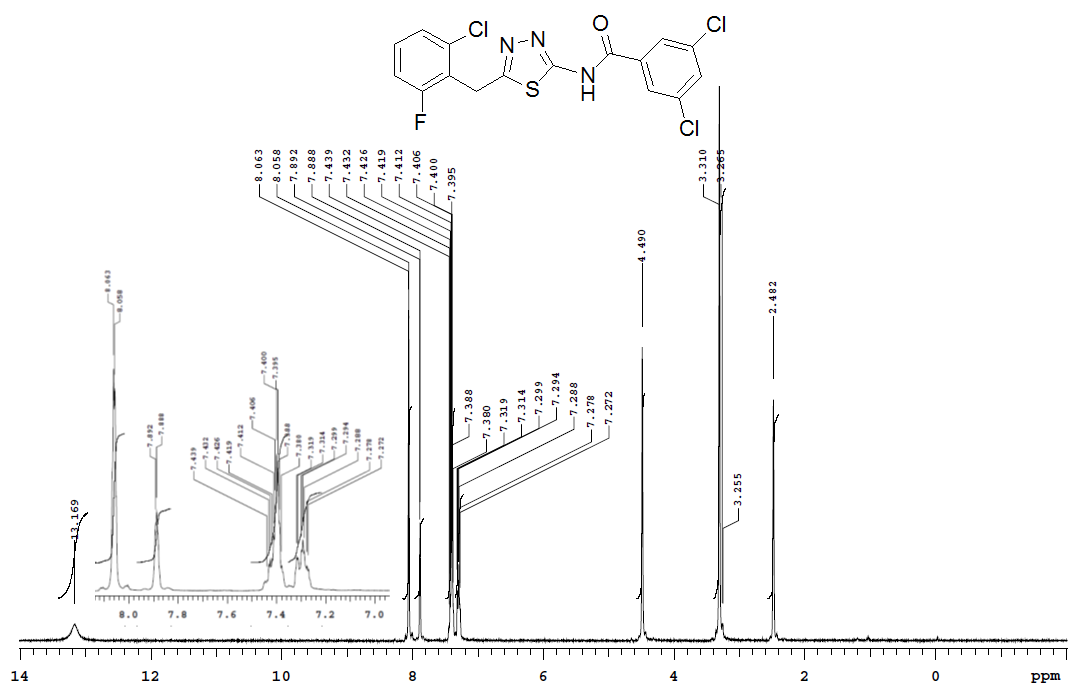


**Figure S52**. ^1^H NMR spectrum of compound **7i** (DMSO-d_6_).


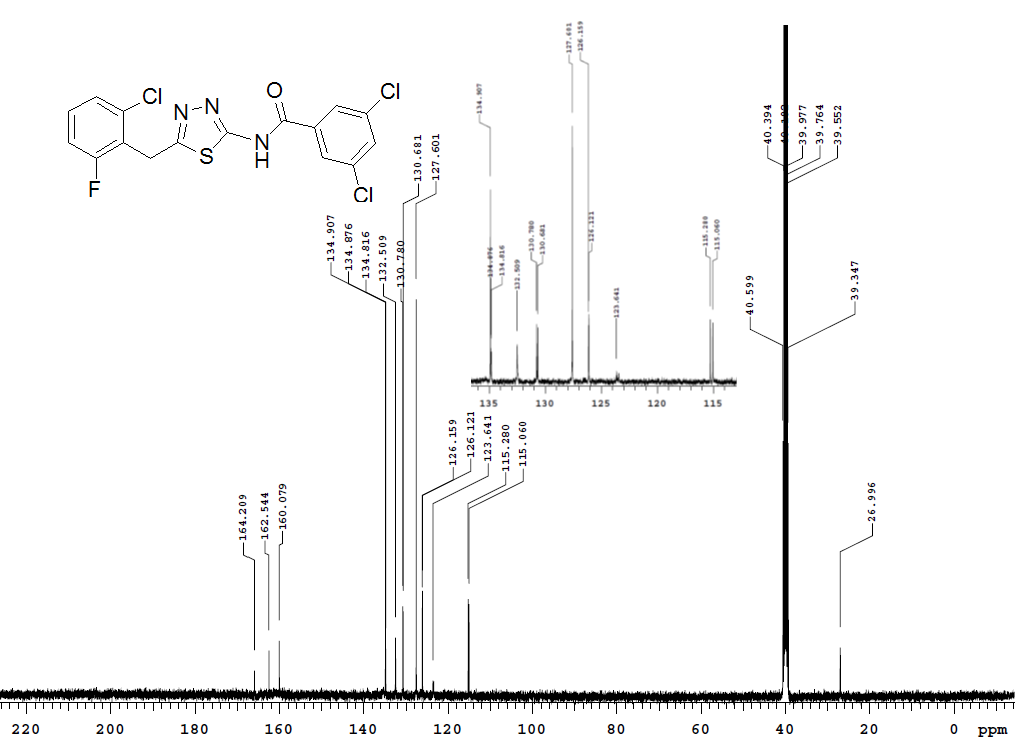


**Figure S53**. ^13^C NMR spectrum of compound **7i** (DMSO-d_6_).


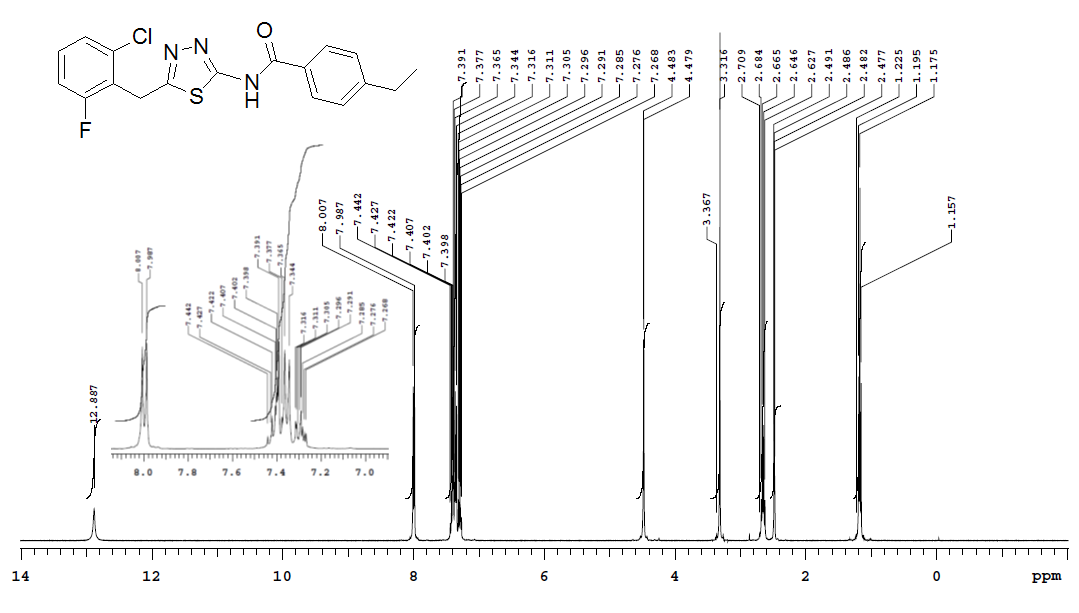


**Figure S54**. ^1^H NMR spectrum of compound **7j** (DMSO-d_6_).

**
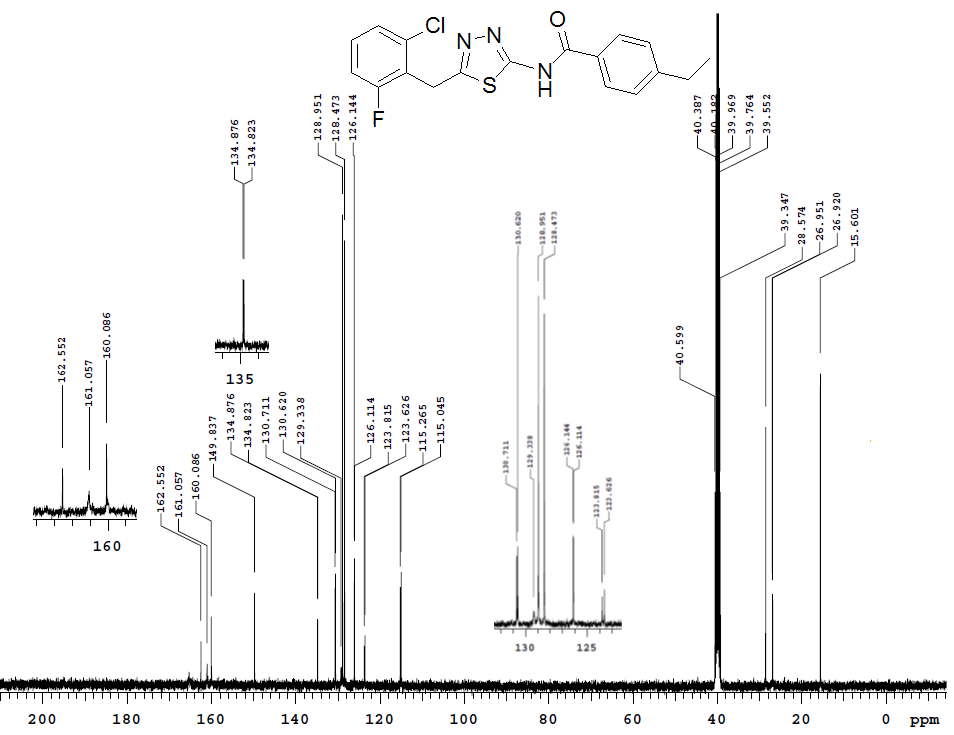
**

**Figure S55**. ^13^C NMR spectrum of compound **7j** (DMSO-d_6_).


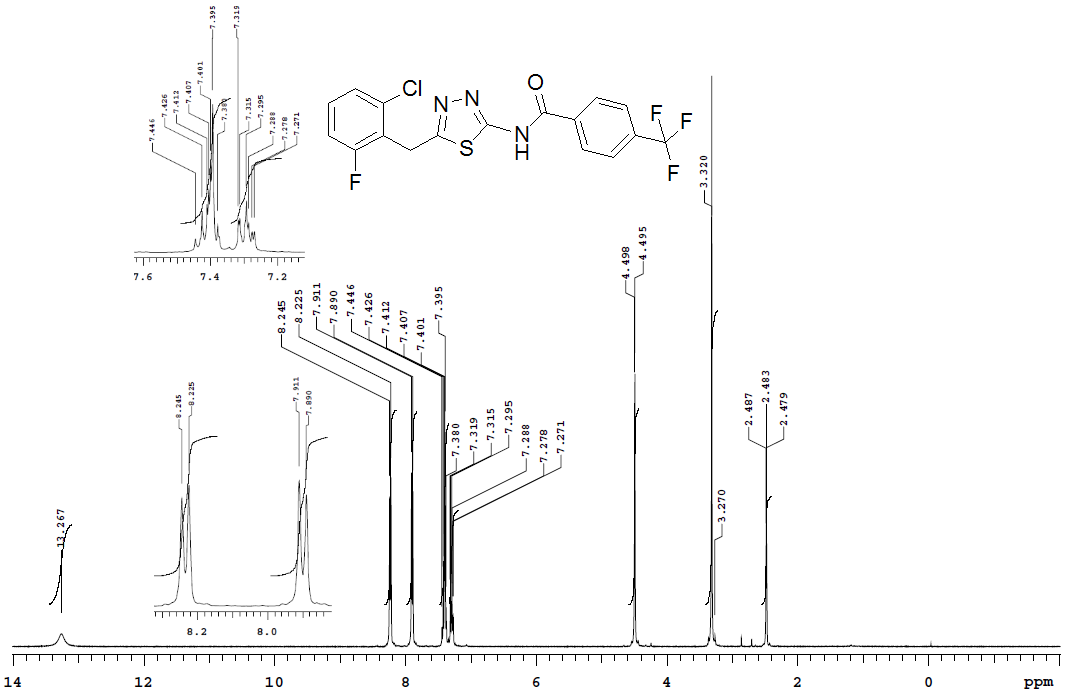


**Figure S56**. ^1^H NMR spectrum of compound **7k** (DMSO-d_6_).

**
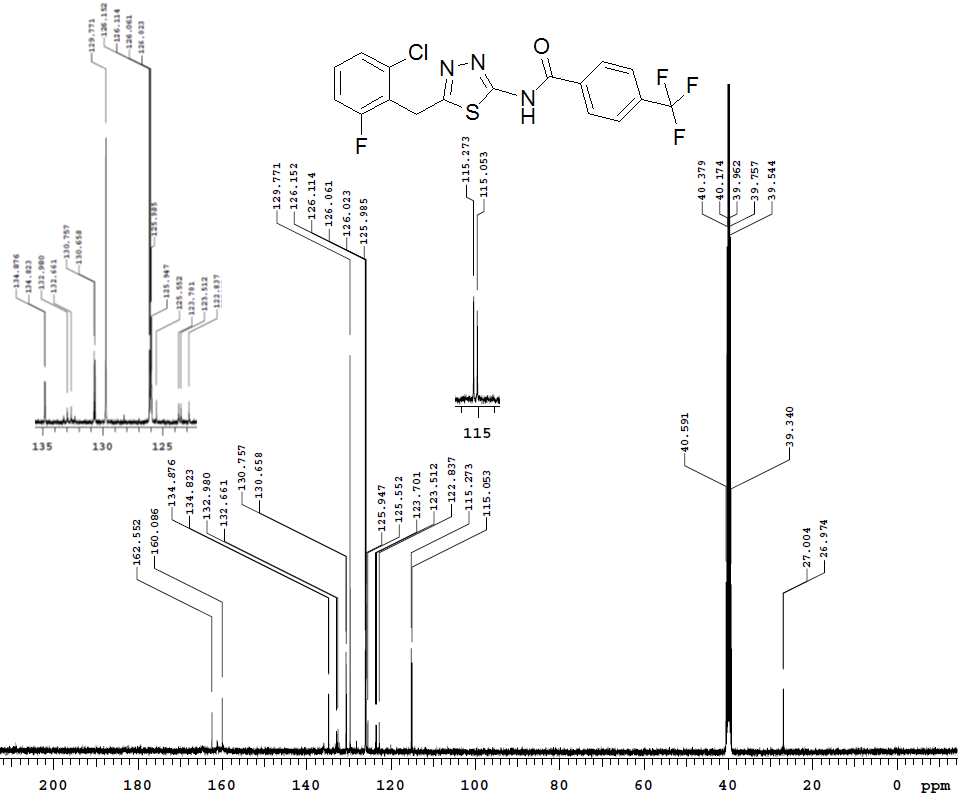
**

**Figure S57**. ^13^C NMR spectrum of compound **7k** (DMSO-d_6_).

**
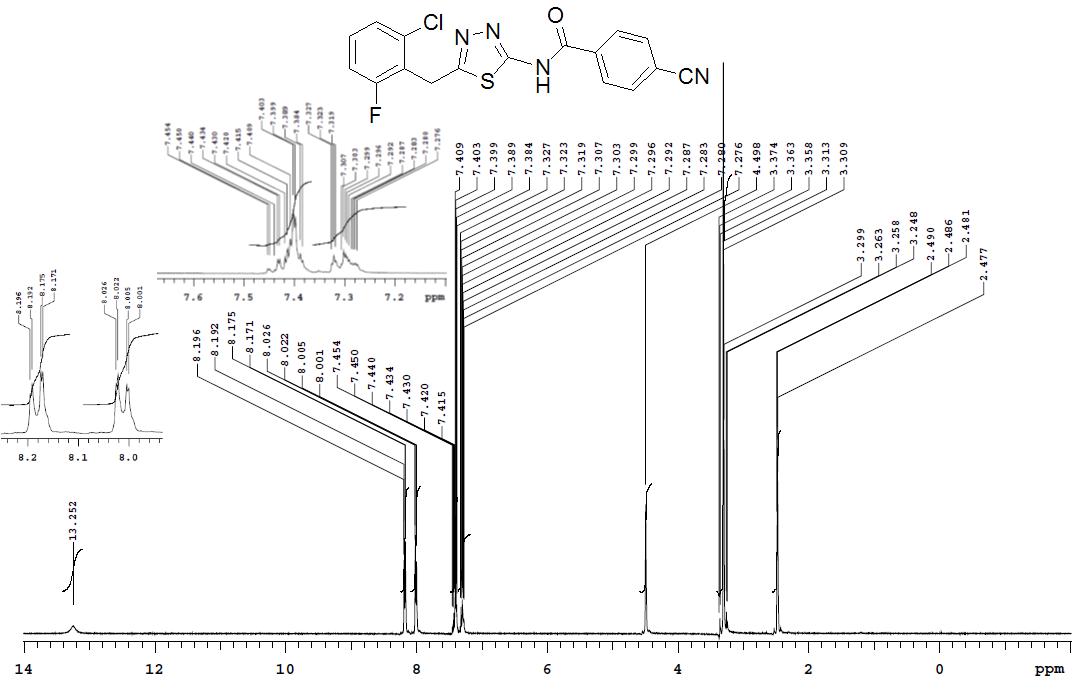
**

**Figure S58**. ^1^H NMR spectrum of compound **7l** (DMSO-d_6_).

**
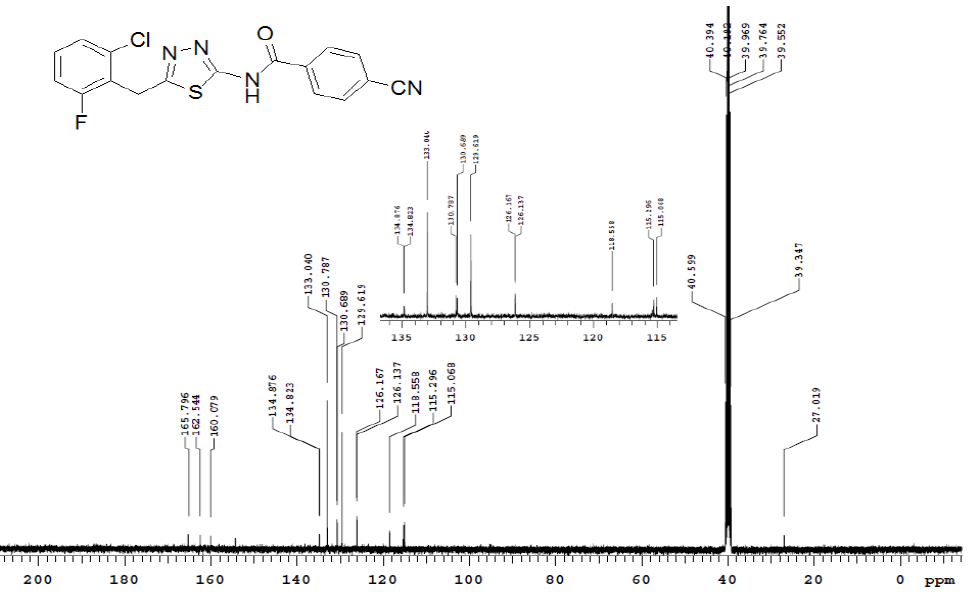
**

**Figure S59**. ^13^C NMR spectrum of compound **7l** (DMSO-d_6_).

**
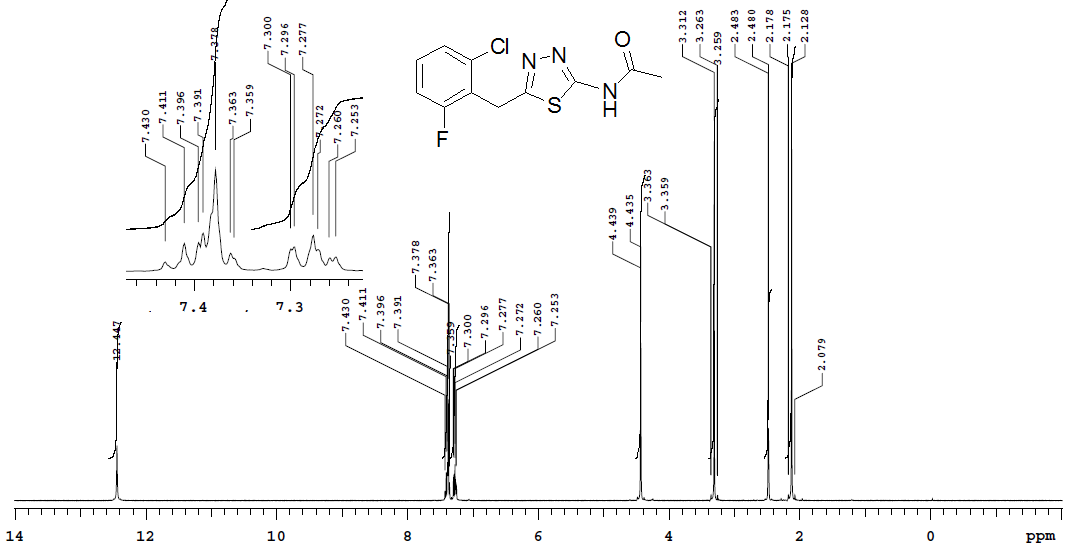
**

**Figure S60**. ^1^H NMR spectrum of compound **7m** (DMSO-d_6_).

**
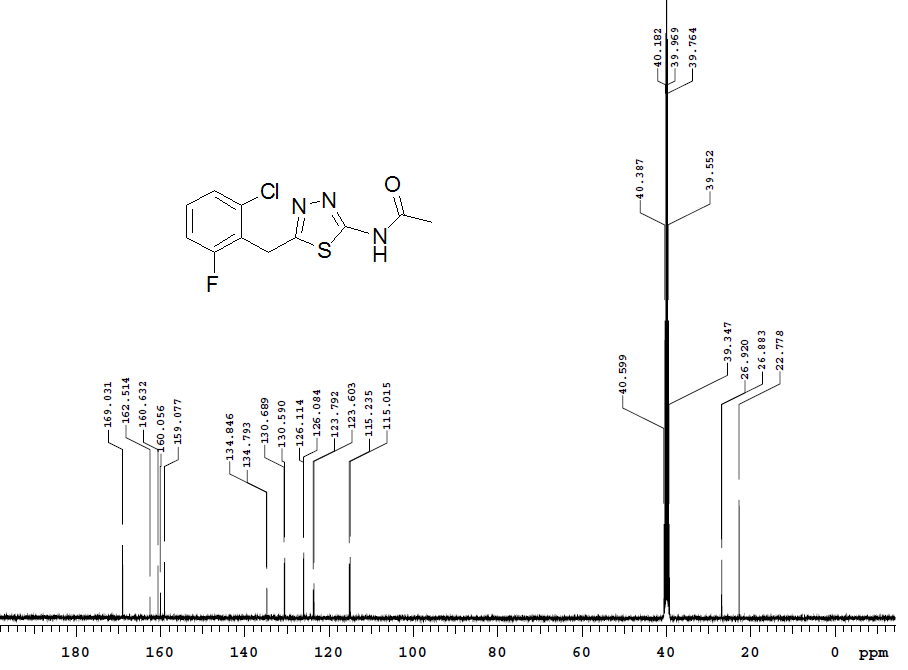
**

**Figure S61**. ^13^C NMR spectrum of compound **7m** (DMSO-d_6_).


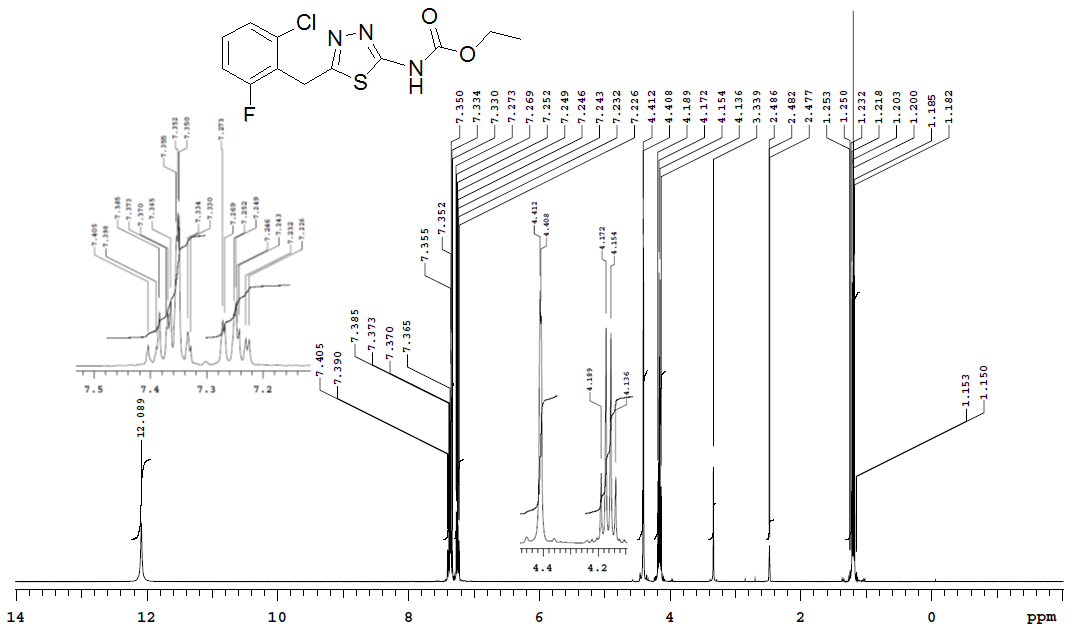


**Figure S62**. ^1^H NMR spectrum of compound **7n** (DMSO-d_6_).

**
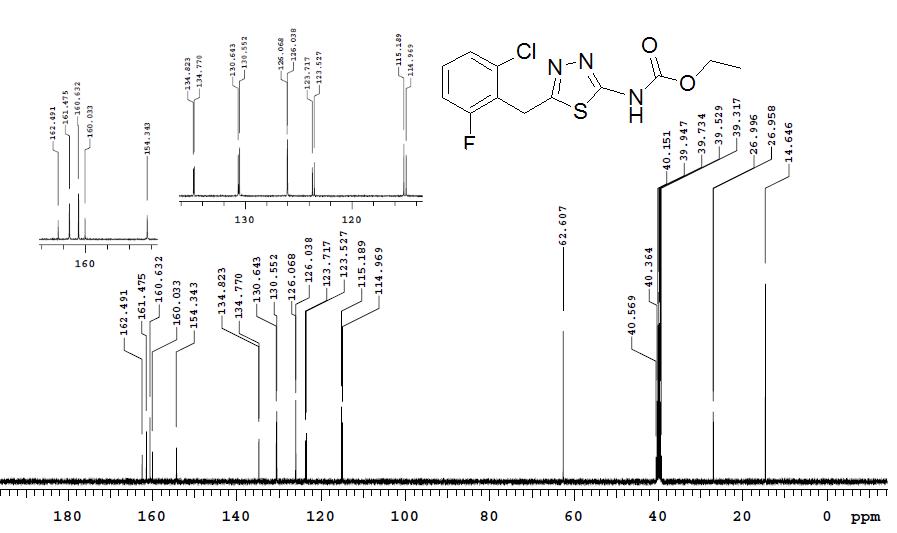
**

**Figure S63**. ^13^C NMR spectrum of compound **7n** (DMSO-d_6_).
